# Shaping the Innate Immune Response Through Post-Transcriptional Regulation of Gene Expression Mediated by RNA-Binding Proteins

**DOI:** 10.3389/fimmu.2021.796012

**Published:** 2022-01-11

**Authors:** Anissa Guillemin, Anuj Kumar, Mélanie Wencker, Emiliano P. Ricci

**Affiliations:** ^1^ LBMC, Laboratoire de Biologie et Modelisation de la Cellule, Université de Lyon, ENS de Lyon, Universite Claude Bernard Lyon 1, CNRS, UMR 5239, INSERM, U1293, Lyon, France; ^2^ CRCL, Centre de Recherche en Cancérologie de Lyon, INSERM U1052, CNRS UMR 5286, Lyon, France; ^3^ CIRI, Centre International de Recherche en Infectiologie, Université de Lyon, ENS de Lyon, CNRS, UMR 5308, INSERM, Lyon, France

**Keywords:** innate immunity, RNA-binding proteins, RNA, virus, immune cells, post-transcriptional regulation, inflammation, pathogen

## Abstract

Innate immunity is the frontline of defense against infections and tissue damage. It is a fast and semi-specific response involving a myriad of processes essential for protecting the organism. These reactions promote the clearance of danger by activating, among others, an inflammatory response, the complement cascade and by recruiting the adaptive immunity. Any disequilibrium in this functional balance can lead to either inflammation-mediated tissue damage or defense inefficiency. A dynamic and coordinated gene expression program lies at the heart of the innate immune response. This expression program varies depending on the cell-type and the specific danger signal encountered by the cell and involves multiple layers of regulation. While these are achieved mainly *via* transcriptional control of gene expression, numerous post-transcriptional regulatory pathways involving RNA-binding proteins (RBPs) and other effectors play a critical role in its fine-tuning. Alternative splicing, translational control and mRNA stability have been shown to be tightly regulated during the innate immune response and participate in modulating gene expression in a global or gene specific manner. More recently, microRNAs assisting RBPs and post-transcriptional modification of RNA bases are also emerging as essential players of the innate immune process. In this review, we highlight the numerous roles played by specific RNA-binding effectors in mediating post-transcriptional control of gene expression to shape innate immunity.

## 1 Introduction

Host’s defense mechanisms form a complex interplay between molecular and cellular actors and require a plethora of processes to detect and eliminate pathogens or damage, such as loss of tissue integrity, irritants, or cancer. While infection or damage generally occur at barrier sites (at the interface between internal and external milieu), local cells are prone to rapidly sense any tissue dysregulation and to send signals of danger that initiate the innate arm of immune responses. Local cellular sensors include epithelial cells, stromal cells and fibroblasts, whose role will be essential when being the initial target of infection (in case of strictly intracellular pathogens) or injury (1–4). In addition, resident immune cells, such as macrophages and dendritic cells (DC), if not directly affected, will be able to detect microbial components within the environment, or capture damaged cellular material leading to their subsequent activation (5). Sensing of damage leads to a cascade of events that generate a local inflammation, thus allowing the recruitment of circulating innate cells (*e.g.* neutrophils or circulating monocytes/macrophages), phagocytosis of infected/damaged cells and antigen presentation to T- and B- lymphocytes, the adaptive arm of immune response. In that regard, innate immune responses are crucial for the generation of a robust, antigen-specific adaptive response, and the maintenance of memory (6, 7).

**Figure 1:**
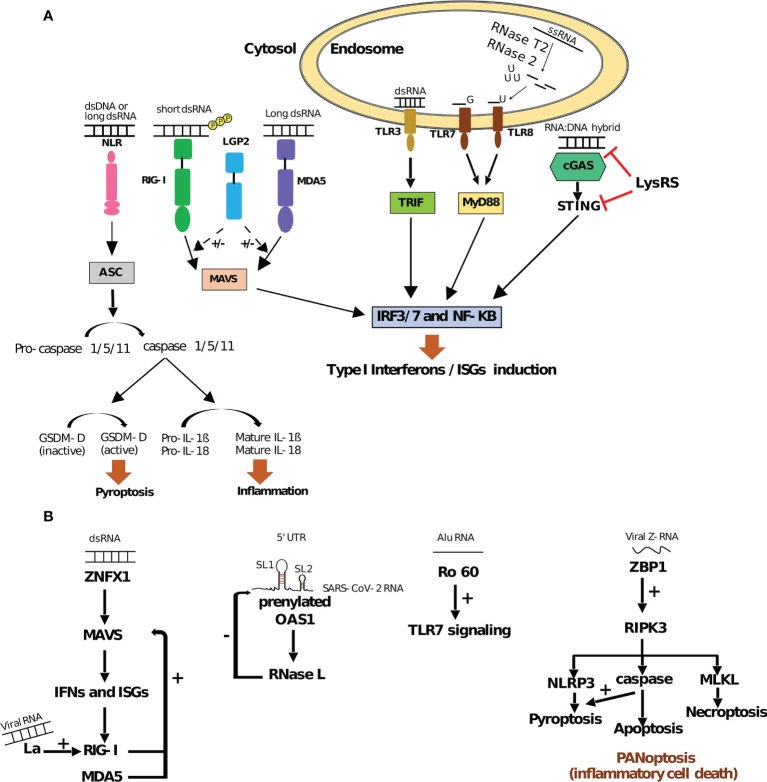
RNA binding PRRs and their regulation **(A)** Innate immune signaling pathways triggered by RNA binding PRRs. Interaction of endosomal TLRs (TLR3, TLR7 and TLR8), cytosolic RLRs (RIG-I and MDA5) and cGAS with different RNA substrates activates IRF3/7 and NF*κ*kB pathway in TRIF/MyD88, MAVS and STING dependent manner, respectively. This activation leads to an induction of ISGs (IFN stimulated genes) as a part of the type I IFN response. On the other hand, NLRs (*e.g.* NLPRP1/3) promote apoptosis speck-like protein (ASC)/caspase-mediated signaling. Caspase-dependent cleavage of Gasdermin-D (GSDM-D) and maturation of pro-IL-1*β* and pro-IL-18 lead to pyroptosis and inflammation, respectively. All these pathways contribute to developing cellular innate immunity and counteracting the effect of different pathogens. While LGP2 can positively (+) and negatively (–) regulate RIG-I and MDA5 pathways, LysRS inhibits the cGAS-STING pathway (9, 64, 75–77). **(B)** RNA sensing by non-PRR RBPs. Upon dsRNA binding, ZNFX1 driven type I IFN response *via* MAVS leads to the expression of ISGs: RIG-I and MDA5, which autoregulate their expression by a positive feedback loop. In addition, La, an autoantigen, could promote RIG-I induction upon viral RNA binding. Sensing of dsRNA structures containing stem-loops 1 and 2 (SL1 and SL2) within SARS-CoV-2 5’UTR by prenylated OAS1 promotes RNase L mediated degradation of the viral RNA and hence displays negative effect (–) on SARS-CoV-2. Ro60 interaction with Alu RNA elicits innate immunity by positively regulating TLR7 signaling. ZBP1 interaction with viral or host Z-RNA activates NLRP3 signaling by positively regulating RIPK3, which encourages the NLPRP3 pathway directly or *via* caspases. These pathways (along with MLKL mediated necroptosis) lead to PANoptosis or inflammatory cell death (78–85).

At the molecular level, innate responses thus start with the recognition of danger. This is allowed by the expression of a set of receptors, called Pattern Recognition Receptors (PRRs), each being specific for classes of molecules known as Pathogen Associated Molecular Patterns (PAMPs) or Damage Associated Molecular Pattern (DAMPs) (8). Essentially all cells express a set of PRRs, the most common families being Toll-like receptors (TLRs), RIG-I-like receptors (RLRs), NOD-like receptors (NLRs) or cGAS (9). PRRs can be discriminated according to their specificity (*e.g.* double-stranded (ds) or single-stranded (ss) RNA, dsDNA, peptides…), or their location (*e.g.* cytosol, mitochondria, extracellular domains). Upon ligand binding, each family of PRRs recruits a specific adaptor such as myeloid differentiation primary response 88 (MyD88), mitochondrial antiviral signaling protein (MAVS) or stimulator of interferon genes (STING) for TLRs, RLRs or cGAS, respectively (**Figure 1A**). This initiates a signaling cascade, mostly involving IRF3/7, MAPK and NF*κ*B pathways, ultimately leading to the expression of type I and III IFN (10, 11) and/or the secretion of cytokines, that orchestrate the various events happening during the inflammation process (12, 13), or chemokines, that will attract immune cells to the affected tissue. Depending on many parameters such as the nature of the activating signals or the timing, cytokines produced are either pro-inflammatory (*e.g.* TNF-*α*, IL-6, IL-1*β*, *etc*…), or anti-inflammatory (*e.g.* IL-4, IL-10, IL-13, IFN-*α*, *etc*…) (14, 15), even though this dichotomy has been shown to be realistically less simple (16).

Altogether, innate immunity plays an essential role during immune responses: guardian of tissue integrity, local cells act as a communication platform to detect, alert and, at least partially, eliminate infection or damage. However, the complex underlying program of innate responses has to be tightly regulated to avoid any hazardous effect. Indeed, overexpression of inflammatory components can damage the host (*e.g.* intolerance, auto-immunity), whereas their under-expression leads to an inefficient defense strategy (17, 18). This highlights the necessity of a rapid and efficient innate response, balanced with appropriate transcriptional and post-transcriptional regulations that finely tune the underlying gene expression program. While transcriptional regulation clearly plays a major role during inflammation and has been widely studied as a model for cell stimulation (19, 20), post-transcriptional control more recently emerged as indispensable to properly tune the innate response (21–23).

RNA-Binding Proteins (RBPs) are key effectors of post-transcriptional regulatory processes (24–26) by targeting specific sequences, structures or post-transcriptional chemical modifications occurring on RNA bases, such as N^6^-methyladenosine (m^6^A). Altogether, RBPs act at all steps of the life of RNAs: pre-RNA processing (5’ RNA capping, splicing, polyadenylation and base editing), mRNA transport, ribosome biogenesis, translation, and finally RNA decay (27–29). In line with this, recent system-wide analyses have revealed the importance of the RNA binding proteome (RBPome) during viral infections (30, 31) and activation of cells of the innate immune system (32). More recently, the global analysis of RNA–protein interactome has shown that a third of the RBPome is remodeled upon SARS-COV-2 infection in human cells, highlighting the importance of targeting RBPs for therapeutic strategies against COVID-19 (33, 34).

Here, we review the multiple roles played by RBPs in shaping the innate immune response, from pathogen and danger detection to the regulation of signal transduction and effector functions.

## 2 Technical Approaches to Study RBPs

Advances in mass-spectrometry and high-throughput sequencing have substantially increased the list of cellular proteins which are known to interact with RNAs and allowed, for many of them, to identify their precise RNA binding sites. Depending on the biological questions to be addressed, RBP’s studies can be performed *via* RNA-centric or protein-centric methods (35). One way to capture protein/RNA interactions is through cross-linking, which creates a covalent bond between the RNA molecule and its associated protein. Different RNA-Protein cross-linking methods exist, each with its own benefits and limitations. In order to identify proteins that interact directly with RNA, irradiation of cells with ultraviolet (UV) light (254nm) is the most commonly used approach, since it irreversibly cross-links amino acid residues to nucleic-acids, without inducing protein-protein covalent bonds. Additionally, the covalent bond created between the amino-acids and RNA bases can provide information related to precise binding site at single-nucleotide resolution (36). However, the efficiency of UV-crosslinking can differ depending on the nature of the amino acids involved in the interaction with RNA bases (37). Furthermore, RBPs that bind to dsRNA structures have been shown to cross-link poorly when exposed to UV radiation due to low accessibility of the RNA bases to the amino acids residues involved in RNA binding (38, 39). Finally, the overall efficiency of RNA/protein cross-linking induced by 254nm UV light is relatively low (5%) (40, 41) and UV light does not penetrate complex tissues or liquid cultures very efficiently, thus limiting its use to cell monolayers or requiring dissociation of the tissue prior to UV irradiation (42). Some of the drawbacks of classical 254nm UV cross-linking can be overcome by the use of photoreactive ribonucleoside analogs 4-thiouridine (4SU), or 6-thioguanosine (6-SG). These ribonucleoside analogs are fed to cultured cells, incorporate into nascent transcripts and allow more efficient cross-linking of RNA to proteins following irradiation with long-wavelength UV light (365nm) that also penetrates samples more efficiently than the commonly used short-wavelength UVs (28). Moreover, this method, known as Photoactivatable-Ribonucleoside-Enhanced Crosslinking (PAR-CL), is also able to score the transitions from thymidine (T) to cytidine (C) that occur at cross-linking sites upon reverse-transcription, which are used to identify precisely the RBP binding sites (43). Methylene blue can be an efficient alternative to UV irradiation when studying dsRBPs. It intercalates between RNA bases of a dsRNA structure and allow efficient dsRBP/RNA crosslinking through visible light, thus overcoming the poor cross-linking activity of UV (38, 44). Formaldehyde can also be used to cross-link single-strand and double-strand RBPs to their RNA targets (39, 45–48). It also has the advantage to be reversible upon incubation of samples at high temperature, which can facilitate RNA recovery and downstream processing such as reverse-transcription. However, formaldehyde cross-linking is not restricted to RNA/proteins but also generates covalent bonds between proteins. As a consequence, ribonucleoprotein complexes can be cross-linked together, therefore making it impossible to discriminate between direct RBPs and non-RBPs that interact indirectly with RNA. Nevertheless, this drawback has been useful in determining the target sites of closely related RNP complexes such as different sub-types of the exon-junction complex (49).

Upon cross-linking, RNA-centric protocols rely on the purification of poly(A) mRNAs (corresponding to protein coding RNAs) (50), total RNA (that gives information on all RNAs including mRNAs and non-coding RNAs such as ribosomal RNA and microRNAs among other) (51), or specific RNA species (52). The RNA purification step is then followed by the identification and quantification of RBPs that were bound to the purified RNAs by mass spectrometry or western-blotting (53). Variations of these protocols combined with purification of poly(A) mRNAs with partial protease treatment have also enabled the characterization of the protein domains that are involved in RNA recognition (54). Altogether, these protocols have allowed the unbiased identification of RBPs in a wide-variety of organisms and biological contexts, but also of new types of RNA recognition domains within RBPs (55, 56). They have also been used to characterize the dynamics of RBP binding to RNA during a wide range of physiological and pathological processes (31, 32). For instance, comparison of the RBPome between resting and LPS-stimulated macrophages uncovered 91 RBPs not previously annotated to interact with RNA. Among identified RBPs, many displayed changes in their RNA-binding capacity upon LPS-stimulation (32). These include the HSP90 co-chaperone P23, which interacts with the mRNA coding for Kinesin 15 (KIF15) (32, 57). Upon macrophage activation, P23 binding to Kif15 mRNA decreases, leading to mRNA destabilization and down-regulation of KIF15 protein abundance, which stimulates macrophage migration (57).

In contrast to RNA-centric approaches, protein centric methods rely on the immuno-purification of a specific RNA-binding protein upon cross-linking (also known as cross-linking immuno-purification or CLIP) followed by RNA-sequencing in order to identify the RNA species bound to the RBP of interest. One can access to the precise RNA sequence where the RBP is binding by coupling immuno-purification with a RNase treatment and small-RNA sequencing (58). Several protocols exist, relying on UV or formaldehyde cross-linking, that allow either the characterization of the RNA-binding sites of isolated RBPs or protein complexes with RNA-binding capacity. Among these protocols, those relying on short and long wave UV crosslinking [such as iCLIP (59), eCLIP (60) and PAR-CLIP (43)] are largely the most frequently used in the literature thanks to their single-nucleotide resolution, availability of commercial kits for sample preparation and development of many data analysis pipelines [some with intuitive graphic user interfaces (61)] that facilitate processing of the sequencing results for non-experts. Furthermore, UV-based CLIP-seq protocols [specifically eCLIP (60)] concentrate most of the efforts made by the Encyclopedia of DNA Elements (ENCODE) project to provide large-scale characterization of the RNA binding sites of hundreds of RBPs in a robust and reproducible manner, providing the scientific community with homogeneous datasets that can be compared across different RBPs and cell types (62). More recently, a CLIP-seq protocol using short laser pulses of UV-light through different time lengths has been able to uncover the *in vitro* kinetics of binding and dissociation of the RBP DAZL to a thousands of RNA targets (e.g. Thbs1 transcript) in a transcriptome-wide manner (63). Once applied to other RBPs, such a protocol could greatly improve our understanding of the biological roles of RBPs and the regulation of their binding and functional activity on their target RNAs.

Altogether, RNA-centric and protein-centric protocols have greatly participated in our understanding of RBPs and their functional role in a myriad of different cellular processes including the innate immune response.

## 3 RNA Sensing by Canonical PRRs and Other RBPs

While RNAs are essential components of cellular functions, they can also generate protective immune responses as it has been largely described following infection with RNA viruses (*e.g.* Influenza, SARS, Hepatitis, Measles, *etc.*). Although sensing of foreign RNA leads to a substantial and rapid antiviral response, it must be tightly controlled in order to avoid hazardous effects [for extensive review, see (64)]. Similarly, inappropriate host RNA recognition can also occur during several processes such as apoptosis (65) or cancer progression (66), leading to uncontrolled immune responses and/or auto-immunity. The capacity to discriminate between host and foreign RNAs is therefore essential to avoid unsuitable immune responses. PRRs that are specific for RNA components are well-known actors of innate immunity, especially in response to viral infections. They represent, as such, prototypic RBPs. However, several non-canonical PRR RBPs have been recently shown to participate in the sensing and/or regulation of RNA sensing and to have a significant role in modulating innate immunity, as examined below.

### 3.1 RNA Sensing by Canonical PRRs

Foreign RNAs can display specific features that distinguish them from endogenous RNAs and allow them to trigger an immune response through sensing by PRRs. These features can be linked to their structure, nucleotide composition and chemical modifications, or their subcellular location.

TLR3 was the first PRR described to interact with foreign RNA (67). Among all TLRs, TLR3, TLR7 and TLR8 are able to recognize RNA substrates under different forms: TLR3 can recognize long dsRNAs, as well as short dsRNAs and structured ssRNAs (>35bp) (68, 69), while TLR7 and TLR8 rely on leucine-rich repeats to detect GU-rich ssRNA species (70) but also by-products of ssRNA degradation including endogenous microRNAs and exogenous siRNAs (71–73). TLR3/7/8 are transmembrane proteins localized within endosomal compartments. RNA sensing through these TLRs therefore requires uptake and endocytosis of extracellular RNAs which generally occurs during viral infection or phagocytosis of necrotic/apoptotic cells (67, 74). This allows a spatial compartmentalization of RNA-binding to TLRs, to avoid any activation by host cell RNAs. Upon activation of TLRs, Toll/IL-1 receptor (TIR) domain-containing adaptor inducing interferon-*β* (TRIF)-dependent pathway is activated, leading to the phosphorylation of transcription factors such as IFN regulatory factor 3 (IRF3) and IRF7 and their subsequent nuclear translocation. IRF3/7 thus turn on the antiviral response in infected host cells, through specific transactivation of type I IFN genes (see **Figure 1A**). In addition, both TLR7 and TLR8 contain two binding sites that synergize to induce TLR dimerization and activation. One binding site is for small ligands (including guanosine for TLR7 and uridine for TLR8) and one for short oligonucleotides (73, 86). For TLR8, the generation of those specific ligands requires both the endolysosomal endonucleases RNase T2 and RNase 2, each cleaving ssRNA upstream and downstream of uridines, respectively, to generate free uridine and short RNA oligonucleotides (71, 87). For TLR7, the source of guanosine and short nucleotides required for its activation are less well-characterized. RNAse T2 is seemingly required for activation of TLR7 in macrophages (88) and crystallography studies suggest that successive U-containing ssRNA sequences are required for full binding to TLR7 (89). Studies in macrophages moreover suggested that accumulation of nucleosides in the lysosomal compartment upon inactivation of the lysosomal transmembrane protein SLC29A3 (a nucleoside transporter from lysosomes to the cytoplasm) leads to TLR7 activation following phagocytosis of necrotic cells (90). Interestingly, in monocytes, macrophages, and dendritic cells, the accumulation of nucleosides within lysosomes is responsible for inflammatory disorders like histiocytosis, further emphasizing the importance of a tight control of RNA mediated TLR activation (91).

Unlike TLRs, whose expression depends on TLR subtype and cellular subset, retinoic acid-inducible gene I (RIG-I)-like receptors (RLRs) are expressed in most cell types. Located in the cytosol, members of the RLR family include RIG-I, the melanoma differentiation-associated protein 5 (MDA5) and the laboratory of genetics and physiology 2 (LGP2) factors. All three RLRs share a central ATP-dependent helicase domain and a carboxy-terminal domain (CTD), both domains displaying RNA-binding activity. RIG-I and MDA5, but not LGP2, have two additional caspase activation and recruitment domains (CARDs) that are essential for downstream signal transduction through MAVS, upon RNA binding. RIG-I recognizes short dsRNA structures bearing a triphosphate or diphosphate group at their 5’ end (5’-PPP or 5’-PP) (92–94) and lacking a methyl group at the 2’-O position of the 5’ terminal nucleotide (95). These structural features, present in many viral RNAs, but generally absent in endogenous cytosolic RNAs, allow RIG-I to discriminate between self and non-self RNAs. MDA5 on the other hand preferentially binds long dsRNAs molecules (including the synthetic poly(I:C) molecule), as shown by several reports (96–98).

However as opposed to RIG-I, the molecular determinants of MDA5 binding to foreign RNAs are less well understood. The presence of higher order RNA structures (combination of single-stranded and double-stranded RNA structures) appears to be important (97), as well as AU-rich sequences, although not specifically under the form of RNA duplex structures (99). Upon recognition of its target RNA, MDA5 oligomerizes into filaments in a cooperative manner and the CARDs domains allow the nucleation of MAVS leading to its activation (100). Interestingly, the ATP hydrolysis activity of MDA5 is stimulated by dsRNA binding and favors the dissociation of MDA5 at a rate that is inversely proportional to the length of the dsRNA substrate. This could explain the length requirement for dsRNAs to trigger MDA5-dependent signaling (101). Finally, the third member of the RLR family, LGP2, displays RNA-binding activity but lacks the CARDs domains required for downstream signal transduction. LGP2 recently emerged as both a positive and a negative regulator of RIG-I and MDA5 activities [for a recent review, see (75)], as supported by the fact that LGP2 deficient mice display disparate susceptibility to infection with RNA viruses (see **Figure 1A**) (102).

Finally, inflammasome-forming nucleotide-binding domain leucine-rich repeat (NLR) proteins are a group of cytosolic PRRs that assemble inflammasome in response to PAMP and DAMPs. Briefly, inflammatory ligand recognition by NLR leads to the recruitment of the apoptosis speck-like protein (ASC) adaptor, allowing the activation of caspase-1. Caspase-1 dependent cleavage of both Gasdermin-D and pro-IL-1*β* or pro-IL-18, further induces cell death by pyroptosis or inflammation, respectively [**Figure 1A**, for a review, see (103)]. NLRs may rely on other PRRs and RBPs to trigger inflammasome activation and, for some of them, direct ligands remain to be characterized. For example, NLRP1 (nucleotide-binding domain leucine-rich repeat protein 1), was one of the first inflammasome-forming PRRs to be identified but its role in pathogen defense and its direct ligands were poorly understood. However, a recent report has shown that human NLRP1 can bind both dsDNA and dsRNA through its leucine-rich repeat domain, but only long dsRNAs [including poly(I:C)] are able to trigger NLRP1 activation (104). Interestingly, in several cell lines and primary cells tested (including primary human epidermal keratinocytes and immortalized human HBEC3-KT bronchial epithelial cells), the sensing of poly(I:C) or infection with Semliki Forest Virus (a positive-strand RNA virus) and its effect on cell viability are fully dependent on NLRP1 expression thus suggesting a non-redundant role of this NLR in sensing dsRNA and triggering activation of the inflammasome (104).

### 3.2 RNA Sensing and Regulation of PRR Activity by Non-PRR RBPs

PRRs are receptors of host defense mechanism that identify pathogens by sensing specific patterns (9). In addition to the layer that relies on canonical RNA-binding PRRs, numerous RBPs, that are not necessarily specific to immune cells, can interact with a large variety of RNAs in order to directly trigger an innate immune response or modulate the activity of canonical PRRs (see **Figure 1B**).

ZNFX1 (zinc finger NFX1-type containing 1) is a member of the helicase superfamily 1 (SF1) localized in the outer membrane of the mitochondria. Similar to RIG-I and MDA5, ZNFX1 can bind viral dsRNAs and interacts with MAVS, in order to promote IFN expression and IFN-stimulated genes (ISGs) (see **Figure 1B**) (78). However, ZNFX1 appears as an early sensor of dsRNA: as opposed to RIG-I and MDA5 whose expression and mitochondrial translocation is induced only following viral infection, ZNFX1 is constitutively localized within mitochondria and its expression, further increased by viral infection, reaches a peak much earlier than RIG-I and MDA5. ZNFX1 has further been shown to enhance the expression of RLRs, therefore priming the subsequent antiviral defense (78). As a consequence, mice deficient for ZNF1X show increased susceptibility to viral infection (78) and those results have been recently confirmed in human with biallelic ZNFX1 deficiencies (105). Thus, ZNFX1 is seen as an early sensor for viral RNAs able to trigger a rapid antiviral response in two different manners: directly through IFN signaling pathway and indirectly through RLRs activation. Interestingly, deficiencies in ZNFX1 are also associated with uncontrolled inflammation following viral infection, in both humans and mice. Although the underlying mechanisms will need further investigation, it highlights the importance of a timely tune innate response, allowing proper elimination of viral spread, while preventing over inflammation. Another recent study involving an ISGs expression screening, has revealed that OAS1 (2’-5’-Oligoadenylate Synthetase 1), a dsRNA sensor, is able to inhibit SARS-CoV-2 through the action of RNase L (see **Figure 1B**) (80). Indeed, by performing iCLIP against OAS1, infection by SARS-CoV-2 was shown to enhance the RNA-binding activity of OAS1, which interacts primarily with highly structured host RNAs (*e.g.* snoRNAs, lncRNAs, intronic regions of mRNAs) as well as with specific stem loops (SL1 and SL2) located withing the first 54 nucleotides of the 5’UTR of all positive-sense SARS-CoV-2 RNAs (80, 106). Importantly, the sensing of SARS-CoV-2 RNAs is dependent on the C-terminal prenylation of OAS1 (addition of hydrophobic molecules) (80), that leads at its translocation to membranous viral replicative organelles (107). The binding of SARS-CoV-2 dsRNAs by OAS1 activates RNase L, which in turn initiates the cleavage of viral and host RNAs harboring single-stranded UpU and UpA motifs (108).

In addition to dsRNA sensing in the cytoplasm, RBPs that are non-canonical PRRs can also act as sensors within the nucleus and potentiate robust immune responses. For instance, a nuclear matrix protein hnRNP U (also known as SAFA for scaffold attachment factor A) directly binds nuclear viral RNAs from DNA and RNA viruses (109). The infection of mouse Bone Marrow-Derived macrophages (BMDMs) by HSV-1, a DNA virus, or by Vesicular Stomatitis Virus (VSV), a ssRNA virus, induced their recognition by hnRNP U in the nucleus. This sensing triggers its oligomerization, inducing its interaction with chromatin remodelers such as the DNA topoisomerase 1 (TOP1) and SWI/SNF-related matrix-associated actin-dependent regulator of chromatin subfamily A member 5 (SMARCA5). This further activates distal and proximal enhancers of type I IFN and other host defense genes (*e.g.* Oasl1, IL-15, CXCL10, Irf1), therefore promoting robust antiviral responses (109). Thus, with its dual function as a viral RNA sensor and a transcriptional regulator, hnRNP U in the nucleus is an advantage against virus evasion strategy.

More recently, the role of the DEAD-box (Asp-Glu-Ala-Asp motif) or DEAH-box (Asp-Glu-Ala-His motif) helicase proteins during innate immune sensing has emerged in the literature [for a more detailed review, see (110)], with promising contribution in treatment of infectious diseases. They are categorized into two groups, based on their activity: (i) those which directly act as RNA sensors, independently of PRRs such as DDX1 or DHX9, and (ii) those which act as co-sensors of RLRs and NLRs, thus improving their activation such as DDX3 or DDX60. For instance, the first category involves proteins able to bind dsRNA. This is exemplified by DDX1, DHX9 and DHX33 that have been shown to directly interact with poly(I:C) or dsRNA from viruses such as Influenza A or reovirus, in myeloid DCs (111, 112). Thus, sensing by DDX1, DHX9 or DHX33 directly induces the production of IFN-*α*/*β* and/or pro-inflammatory cytokines responses, *via* MAVS (DHX9, DHX33) or TRIF (DDX1), independently of canonical PRRs. In addition, DHX9 (in murine intestinal cells) and DHX33 (in THP-1 macrophages cell line) are able to sense RNA and trigger NLRP9 and NLRP3 inflammasome, respectively (113, 114). The second category includes co-sensors such as DDX3 or DDX60. While DDX3 can directly associate with poly(I:C), it also form a complex with MAVS and RLRs to potentiate type I IFN responses, following stimulation (115). Similarly DDX60 interacts with RLRs to promote their downstream signaling (116).

Similarly, viral RNAs sensing are also modulated by several autoantigens such as La/SS-B (La). La has been recently shown to directly bind RIG-I once bound to a viral dsRNA (79), resulting in strengthened interaction between RIG-I and its RNA ligand and eventually empowered RIG-I-mediated type I and type III IFN production. In addition, La also promotes the activation of MAVS, a mitochondrial-associated adaptor downstream of RIG-I (79) (**Figure 1B**). Indeed, RNA recognition results in RIG-I exposing its activator CARD domains, which in turn binds the CARD domain of MAVS (117). The activation of MAVS leads to the formation of a complex with two other proteins (CARD9 and BCL-10) that eventually turn on the NF*κ*B signaling pathway (118). Therefore, upon infection, La reinforces the activation of MAVS through RIG-I and empowers the immune response. By contrast, Ro60, another viral RNA binding autoantigen, is known to negatively regulate the inflammatory response by buffering the recognition of viral RNAs by RNA sensors. Ro60 is a component of a ribonucleoprotein complex that targets misfolded cellular RNAs (potentially foreign RNAs) for destruction, thus slowing down their detection by immune RNA sensors and delaying the alarm signal (81).

In addition to directly sensing or potentiating detection of viral RNAs, RBPs also modulate immune responses through the direct recognition of endogenous RNAs (119–122). This is for example the case for Alu transposable elements (TEs), which account for over 10% of the human genome (123, 124). Alu elements belong to the Short interspersed nuclear element (SINE) family of transposable elements (125). They are primate specific repeat sequences of around 280bp long, that can be found in intergenic regions but also embedded within introns and exons of protein coding genes and expressed together with the gene in which they are integrated. Functional Alu elements can be transcribed from their own promoter by RNA polymerase III and depend on the reverse transcriptase activity of the ORFp2 protein from LINE-1 elements to reverse transcribe and integrate into new genomic loci (125). Transcribed Alu elements can bind several cellular RBPs such as Ro60, which negatively regulates their abundance (81). In Hela cells, infection with adenovirus type 5 and herpes simplex virus type 1 have been described to activate RNA polymerase III-dependent transcription of Alu TEs (122, 126). Alu TE are also strongly transcribed upon exposure to type I IFN and pro-inflammatory cytokines, both from their own promoter and as part of induced gene transcripts with embedded Alu elements (127). Interestingly, the high abundance of Alu transcripts induced upon IFN exposure has been shown to saturate Ro60 and allow their recognition by TLR7, to further amplify the IFN response through classical signaling pathways (MAPK, NF*κ*B) (81, 128) (**Figure 1B**). In line with this, auto-antibodies bound to Ro60-Alu RNA/protein complexes have been detected in the blood of Sjögren’s Syndrome patients and have been proposed to mediate TLR7-dependent signaling in B cells (upon endosomal uptake), leading to their aberrant activation and production of inflammatory cytokines (81). Ro60 can therefore play a dual role in restricting Alu abundance to limit their recognition by PRRs under physiological conditions, while also being responsible for inducing a pathological innate immune response in the context of an autoimmune disease.

In addition to Alu RNAs transcribed from their own promoter, Alu elements embedded within mRNAs can act as scaffolds for the recruitment of RBPs in the context of innate immunity. Many cellular transcripts harbor two or more embedded Alu copies in inverted orientation (Alu inverted repeat, AIR) mostly located in their 3’UTRs (129). AIRs can base-pair with each other and the closer the inverted Alu sequences are, the more frequently they tend to base-pair with each other (39). The resulting dsRNA structure has been shown to form Z-RNA (a left handed dsRNA helix with Z-conformation) (130) that is recognized by the Z*α* domain of ADAR1 (131) and Z-DNA binding protein 1 (ZBP1) (83) with different outcomes. ZBP1 is a sensor for viral as well as endogenous Z-RNAs, promoting NLRP3 inflammasome and pyroptosis *via* Receptor Interacting Protein Kinase 3 (RIPK3) (83–85). More recently, ZBP1 has also been shown to induce PANoptosis, a form of inflammatory cells death, through a large multi-protein complex named AIM2 PANoptosome (**Figure 1B**) (82). By contrast, ADAR1 appears more like a guardian of homeostasis, by limiting inflammation. Indeed, ADAR1 binding to Z-RNA mediates adenosine to inosine conversion (also known as A-to-I editing) within the dsRNA structure (132). A-to-I editing disrupts the continuity in the dsRNA structure avoiding its recognition by the dsRNA sensors MDA5 and PKR. Interestingly, gain of function mutations in MDA5 associated with human immune disorders such as Aicardi-Goutières syndrome, have been shown to render MDA5 more tolerant to the irregular AIRs dsRNA structures generated upon ADAR1 A-to-I editing and inducing an aberrant antiviral response (133). Similarly, a recent study has shown that ADAR1 could act as a negative regulator of ZBP1 mediated PANoptosis (134). Those results suggest that the specificity of MDA5 and ZBP1 in recognizing its RNA substrates is under strong selection to maintain a trade-off between efficient pathogen recognition and self-tolerance.

Another strategy to regulate immune responses through PRRs sensing is to physically mask host RNAs with RBPs. For instance, under physiological context, a host-derived RNA called 5S ribosomal RNA pseudogene 141 (RNA5SP141) is present in the nucleus. When RNA5SP141 translocates into the cytoplasm, it recruits different RBPs such as ribosomal protein L5 (RPL5) and mitochondrial ribosomal protein L18 (MRPL18), avoiding unwanted activation of the immune system by PRRs. Some DNA viruses [*e.g.* herpes simplex virus 1 (HSV-1), Epstein-Barr virus (EBV) and influenza A virus (IAV)] have been shown to disrupt the nucleus membrane and to induce a global downregulation of host proteins’ synthesis. This leads to increased availability of RNA5SP141 in the cytoplasm and its unmasking from the RBPs (135). RIG-I thus recognizes the RBP-free RNA5SP141, inducing type I IFN stimulation and antiviral immunity. This study shows how RBPs protect host RNAs from PRR recognition and highlights a mechanism used by the host to induce PRR activation during infection by DNA viruses.

Finally, dsRNA or ssRNA are not the only molecules recognized by RBPs. RNAs can base-pair with DNA during different physiological processes (*e.g.* DNA transcription, DNA replication, dsDNA break repairs) or as intermediates of replication for certain viruses and endogenous retroelements (*e.g.* retroviruses, hepadnaviruses and LINE-1 retroelements). Viral RNA : DNA hybrids have been shown in mouse bone marrow-derived conventional DCs (cDCs) and human Peripheral Blood Mononuclear Cells (PBMCs), but not in BMDMs, to be recognized by TLR9, leading to the production of pro-inflammatory cytokines, such as IL-6 and IFN, in a Myd88-dependent manner (136). Similarly, the cyclic GMP-AMP synthase (cGAS), classically seen as a DNA sensor that activates inflammatory response *via* its adaptor STING (137) can also detect RNA : DNA hybrids and induce STING activation leading to IFN-*β* and ISG expression (138). However, this property appears highly regulated, probably to prevent over-inflammation following infection, or unwanted immune responses during biological processes involving RNA : DNA hybrid intermediates. Indeed, the Lysyl tRNA synthetase (LysRS), a component of the cytosolic multi-tRNA synthetase complex (MSC) involved in mRNA translation (139–141), has recently been shown to directly interact with RNA : DNA hybrids and compete with cGAS to delay STING activation and downstream type I IFN response (142). In addition to competing with cGAS, binding of LysRS to RNA : DNA hybrids leads to LysRS-dependent production of diadenosine tetraphosphate (Ap4A), which is able to directly bind STING and prevent its interaction with 2’3’-cyclic GMP-AMP (cGAMP) and decreases downstream production of type I IFN (see **Figure 1A**) (142).

Altogether, RBPs are important players in the direct recognition and/or modulation of the recognition of foreign RNAs and in triggering a robust innate response against a wide-range of pathogens.

## 4 Role of RBPs in RNA Processing During Innate Immunity

### 4.1 Mechanisms of Alternative Splicing

In eukaryotes, protein-coding genes contain sequences that are found in mature mRNAs (exons) and sequences that are removed during mRNA maturation (introns) through a process called splicing and catalyzed by the spliceosome (143–145). By skipping or retaining specific exonic sequences, pre-mRNA splicing can create various RNA isoforms from a single transcription unit in a process known as alternative splicing. The spliceosome is a megadalton machinery composed of small nuclear RNAs (snRNAs) and proteins that associate to form small nuclear ribonucleoprotein particles (snRNPs termed as U1, U2, U4, U5, and U6) (146). Each intron has specific conserved sequences, the splice donor site that defines the 5’ exon/intron junction, the splice acceptor site that defines the 3’ intron/exon junction and the branch site that is essential during the first step of the splicing reaction. These sequences are recognized by snRNPs that assemble in a chronological order to perform two transesterification reactions that lead to the cleavage and release of the intron, while performing phosphodiester bonds to ligate the exons (146). Selection of the splice donor and acceptor sites can be modulated by surrounding cis-acting sequences, such as exonic and intronic splicing enhancers or silencers, that recruit RBPs including serine/arginine-rich family of nuclear phosphoproteins (SR proteins) and heterogeneous nuclear ribonucleoproteins (hnRNPs) [for a short review, see (147)]. Differential recognition of splice donor and acceptor sites leads to the process of alternative splicing, creating multiple transcript isoforms with different coding sequences or alternative 5’UTRs. Alternative-splicing plays a critical role in numerous cellular processes such as the establishment of cell identity or sex selection (148, 149). In the context of immunity, alternative splicing has been shown to play an important role in the differentiation, homeostasis, and regulation of immune cells (150–153). Importantly, it appears as a major regulator of inflammation in innate immune cells. For instance, in mouse macrophages isolated from the lung at different time-point following intra-tracheal injection of LPS, global changes in alternative splicing were observed at the pic of the inflammatory response. In that context, genes involved in cellular metabolism and chemotactism were shown to be highly regulated by alternative splicing. Moreover, alternative splicing appears to regulate different set of pre-mRNA in recruited macrophages (mostly pro-inflammatory) as compared to resident macrophages, likely explaining their different metabolic requirement (154). Similarly, in monocyte derived macrophages obtained from human blood, bacterial infection is associated with global changes in mRNA isoform usage, with increased cassette exon inclusion (155). Finally, numerous important immune molecules transcripts downstream of PRR signaling (*e.g.* MyD88, IL-1 receptor-associated kinase (IRAK)), and even some TLR mRNA themselves also see their expression regulated by alternative splicing to give rise to protein isoforms with differential biological activities (156–158). However, although alternative splicing is mainly modulated by RBPs such as SR and hnRNP proteins, their role in the context of innate immunity is still poorly understood at the molecular level.

### 4.2 Alternative Splicing of PRRs and Their Downstream Signaling Factors

As the key contact between the noxious molecule and the host cells, PRRs and their downstream factors, represent a central hub of regulation. Alternative splicing has been shown to regulate activation and/or functions of those receptors, in order to control the intensity of immune responses and their shutdown upon clearance of infection or damage, as well as to prevent inappropriate inflammatory responses and autoimmunity.

While PRRs play an essential role during innate immunity by sensing specific patterns and alarming the immune system, their activity needs to be downregulated upon activation, to avoid over-inflammation. In line with this, alternative splicing appears as an important mechanism to limit PRR signaling. For example, LPS recognition by TLR4 requires TLR4 interaction with Myeloid Differentiation Factor 2 (MD-2) and the subsequent signaling cascade will lead to the expression of an MD-2 spliced isoform (MD-2s) that will inhibit TLR4 signaling (158). *In vivo*, delivery of MD-2s in the lung substantially decreases LPS-induced inflammation (159). Such a negative feedback loop has also been observed for TLR3, which recognizes dsRNA. In human astrocytes cell lines, TLR3 activation leads to type I IFN production that, in turn, induces the expression of an alternative spliced TLR3 isoform that acts as a negative regulator of TLR3 downstream signaling pathways (160).

In a different context, alternative splicing has been shown to regulate the relative production of membrane-bound and soluble forms of immune receptors, with opposite effect on inflammation. This is the case for Siglec-14, a glycan recognition protein that can elicit pro-inflammatory responses, in response to bacterial pathogens. Siglec-14 is classically paired with Siglec-5, that acts as an inhibitory receptor for bacterial pathogens, providing a first level of regulation in pro-inflammatory processes (161). Thus, in LPS-stimulated neutrophils, Siglec-14 has been found to be up-regulated and Siglec-5 down-regulated, suggesting a positive feed-back loop that increases myeloid inflammatory responses (162). As an additional layer of regulation, it has been found that a soluble form of Siglec-14 (sSiglec14) is able to interfere with the interaction between TLR2 and the membrane-bound form of Siglec-14 (mSiglec-14), in myeloid cells (163). The soluble form of Siglec14 is due to the retention of intron 5 during pre-mRNA splicing, which contains a C-terminal hexapeptide before the translation termination codon (164). While sSiglec14 is involved in the suppression of pro-inflammatory cytokines production (164), these results suggest a negative feedback mechanism regulating the myeloid pro-inflammatory responses elicited by the engagement of mSiglec-14. Thus, the switching between sSiglec-14 and mSiglec-14 might be used by the innate system to control a potential unwanted inflammatory response that could damage the host tissues.

**Figure 2:**
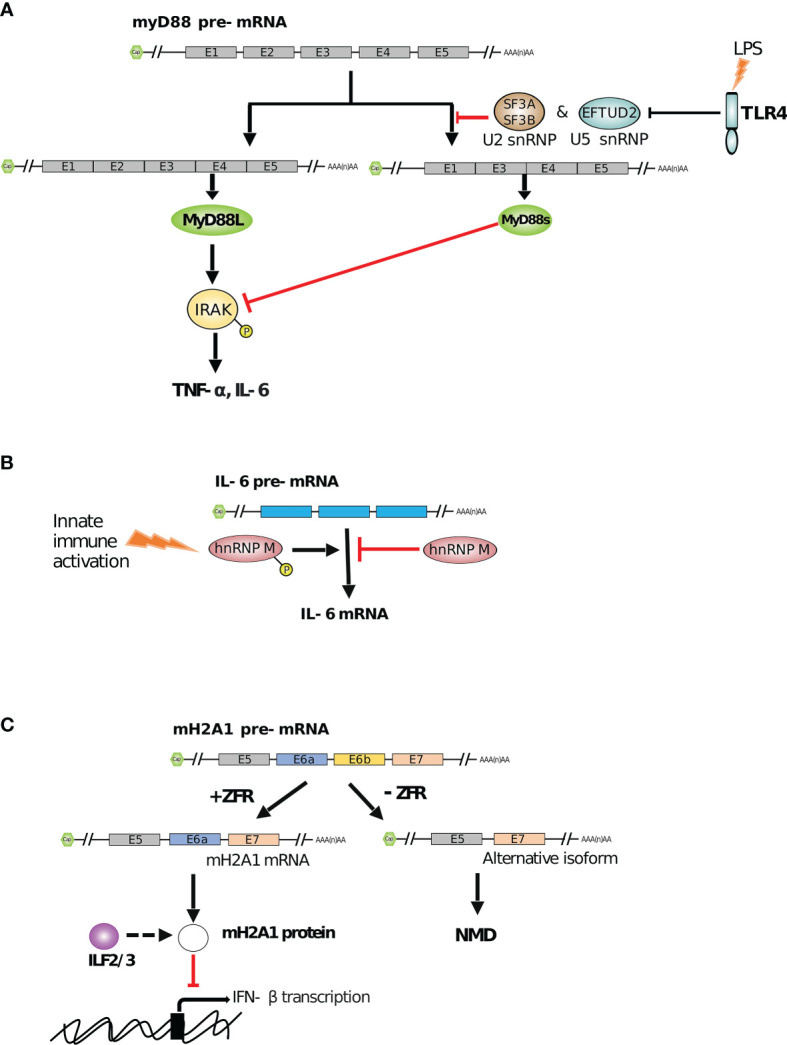
RBP mediated regulation of innate immune functions by alternative splicing. **(A)** The smaller isoform (myD88s) of TLR4 signaling adaptor myD88 inhibits the production of cytokines (such as TNF-α and IL-6) by inhibiting myD88L mediated phosphorylation of IRAK. However, SF3A/3B and EFTUD2, in complexes with other snRNPs, reduce the production of myD88s to maintain cytokine expression (169, 170). **(B)** hnRNP M represses the splicing of IL-6 mRNA. However, innate immune activation in infected macrophages leads to phosphorylation of hnRNP M and relieves this splicing suppression (171). **(C)** ZFR facilitates correct splicing of mH2A1 transcript by exon inclusion which prevents the formation of an aberrantly spliced isoform. mH2A1 protein, which ILF2/3 possibly regulates, further promotes IFN-*β* mRNA expression to enhance type I IFN response (172).

In addition to PRRs, the activity of immune receptor’s adaptors such as MyD88 or TRIF has been shown to be dynamically modulated through alternative splicing (165). The cytoplasmic portion of most of the TLRs shows high similarities with the interleukin-1 (IL-1) receptors family and is thus called the TIR domain, for Toll/IL-1 receptor (165). TIR domain thus serves as a platform to recruit TIR domain-containing adapter, such as MyD88 (166). Upon stimulation, MyD88 recruits IL-1 receptor-associated kinase (IRAK), a serine/threonine kinase, also containing a TIR domain. IRAK is then activated by phosphorylation and interacts with TRAF6. Altogether, this signaling cascade leads to the activation of MAPK and NF*κ*B signaling pathways that are crucial for completion of innate immune response (165). Thus, the regulation of TLR’s adaptors are critical for the control of immune defense. Interestingly, a MyD88 splice variant (MyD88s) induced upon LPS activation in monocytes codes for a protein isoform that lacks the IRAK-interacting domain, thus acting as a dominant negative by preventing IRAK phosphorylation and downstream NF*κ*B activation to inhibit the LPS induced signaling pathway (167, 168). In resting cells, MyD88s alternative splicing is regulated by SF3A, SF3B and EFTUD2 that specifically inhibits the generation of the MyD88s isoform (lacking the IRAK-interacting domain), thus ensuring a robust initial innate response (see **Figure 2A**) (169, 173). Interestingly, in macrophages, SF3A knockdown preferentially affects splicing events related to innate immunity, such as the TLR signaling pathway, highly suggesting a more global role of this splicing factor in controlling the intensity of inflammation (170).

### 4.3 ISG Control by RBP-Mediated Alternative Splicing

In the case of a viral infection, the innate immune system reacts by disrupting the functions and pathways vital for the pathogen’s life cycle. Once PRRs are activated by viral components, antiviral cytokines are produced. Among the earliest cytokines to be produced are type I IFNs (*e.g.*, IFN-*β*, IFN-*α*, *etc.*) that trigger the JAK/STAT signaling pathway in order to induce expression of ISGs (174). These ISGs (*e.g.*, IFIT1-3) generally act as antiviral effectors that control viral replication and spread (175, 176). Modulating ISG expression is critical to manage an efficient defense against pathogens while preventing detrimental adverse immune effects. RNA splicing is one way among others to ensure the proper timing and intensity of ISG expression. Splicing regulation involves one or more splicing factors acting like a regulatory node, as illustrated by heterogeneous nuclear RiboNucleoProteins (hnRNPs) (177). hnRNPs are complexes comprising typical RNA-binding and modular proteins mostly present in the nucleus (178). Using UV cross-linking followed by oligo(dT) purification of RNAs, about 20 species of hnRNPs have been identified (hnRNP A-U) (179). These ‘RNA scaffolds’ play various roles associated with the fate of the RNA such as the regulation of splicing or the transcriptional responses to DNA damage (180–183). It has been recently shown that a loss of hnRNP M results in overproduction of several innate immune transcripts such as antimicrobial factors as well as ISGs (171). In early stages of macrophage activation, the splicing factor hnRNP M associates with nascent IL-6 mRNA and slows down its initial ramping, acting probably as a safeguard of the inflammatory response. Once the macrophage is fully activated, hnRNP M is phosphorylated (downstream of the TLR pathway) and is then released from mRNA transcripts, promoting their splicing and full maturation (see **Figure 2B**) (171). Zinc finger RNA-binding protein, ZFR, is another example of alternative splicing regulation that has been shown to suppress the IFN response by regulating ISGs splicing (172). ZFR contains double-stranded RNA binding motifs as well as several Cys-Cys-His-His (CCHH) zinc finger domains (184). This zinc finger protein has been shown to prevent aberrant splicing of the histone variant macroH2A1 (mH2A1), which in turn binds and represses IFN-*β* activation and ISG expression (see **Figure 2C**) (172). Although the precise mechanisms of both ZFR impact on mH2A1 splicing and mH2A1 negative effect on type I IFN responses still remain to be determined, it again highlights the high level of regulation underlying type I IFN-responses.

### 4.4 Specialized Splicing in Non-Canonical Nuclear Bodies During Inflammation

**Figure 3:**
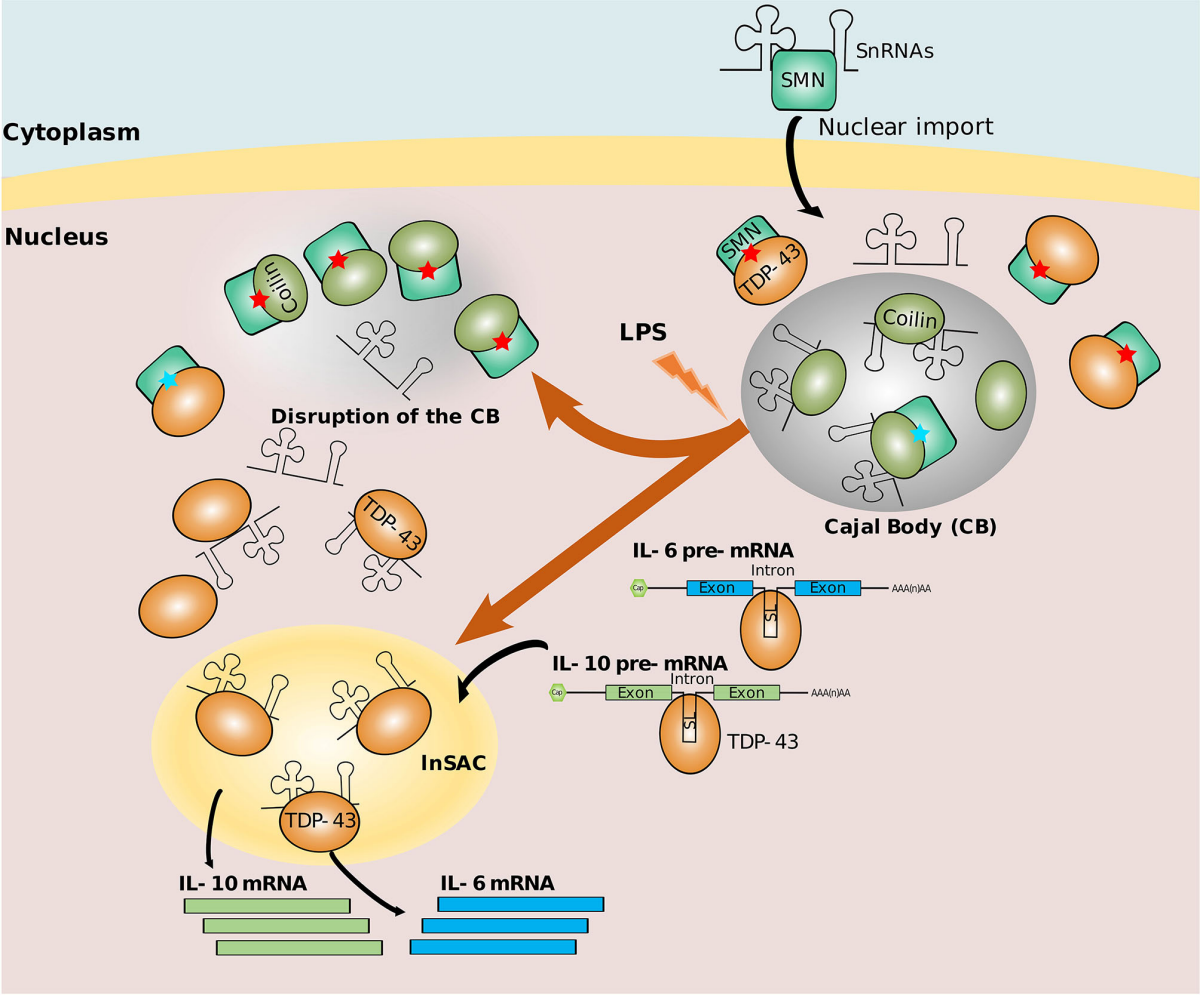
Nuclear bodies for splicing. In a physiological context, Cajal Body (CB) recruits snRNAs and snRNPs for maturation. Coilin interacts with SMN with low affinity (represented by blue stars). In infection context, Coilin strongly interacts with SMN (represented by red stars), and TDP-43 hijacks snRNAs and stabilizes InSAC (a sub-nuclear body favoring the splicing of IL-6 and IL-10). Simultaneously, TDP-43 recruits cytokine transcripts through their putative stem loop (SL) into InSAC and promotes their splicing (189, 190).

The nucleus is highly organized into membrane-less structures called nuclear bodies. Different nuclear bodies have been described, endowed with specific proteins and RNA composition, and functional specificity. One example of such sub-nuclear compartments are Cajal Bodies (CB), that act as an organizer of spliceosomal small nuclear ribonucleoproteins (snRNP) biogenesis (185). On a mechanistic point of view, Coilin, a multivalent scaffold protein, has been shown to interact with small nuclear RNAs (snRNAs), targeting them to CB to achieve snRNP assembly (186, 187). Coilin interacts with U snRNPs and with a CB component called survival of motoneurons (SMN) in order to participate in the formation and integrity of CB themselves (188). In an inflammation context such as LPS-activated macrophages, Coilin interacts with higher affinity to SMN, leading to snRNA release and CB destabilization (**Figure 3**). Simultaneously, Tat-activating regulatory DNA-binding protein-43 (TDP-43), a highly conserved hnRNP involved in RNA processing (*e.g.* splicing, trafficking, *etc*…), becomes ubiquitinylated, decreases its binding to SMN and competitively recruits snRNAs and other components from spliceosomal snRNPs creating a novel sub-nuclear body different from CBs (189). TDP-43 also binds cytokine IL-6 and IL-10 pre-mRNAs in a sequence-specific manner (through short GC-rich palindromic repeats separated by a short spacer with a conserved ‘ACU’ sequence located in intron 2 of IL-6 and intron 1 of IL-10), thereby favoring their splicing within the sub-nuclear body dubbed InSAC (which stands for Interleukin-6 and -10 Splicing Activating Compartment) (see in **Figure 3**) (189). By hijacking components from CBs and controlling the distribution of subnuclear compartments, TDP-43 functions as a scaffold protein within InSACs, which become a specific cytokine pre-mRNA splicing compartment and an important effector of the immune response during inflammation (190).

### 4.5 Polyadenylation

Most mRNAs, with the exception of those coding for canonical histones, bear a 3’ poly(A) tail added during the maturation of mRNA, in a process called polyadenylation. Polyadenylation is a co-transcriptional process involving the recognition of a cleavage/polyadenylation site (typically “AAUAAA” in a GU or U-rich context) in the nascent pre-mRNA, followed by endonucleolytic cleavage of the pre-mRNA (35 nt downstream of the cleavage/polyadenylation signal) and addition of a poly(A) sequence (50-250 nt long) at the 3’ end of the cleavage site (191). An estimated 80% of mammalian genes contain multiple cleavage/polyadenylation sites generally leading to transcript isoforms with alternative 3’ untranslated regions (3’UTRs) (192). Use of alternative polyadenylation (APA) sites is a dynamic process regulated by the abundance of the RNA-binding proteins involved in the polyadenylation process itself or by other RBPs, such as HuR, that can bind close to the cleavage/polyadenylation site and block its recognition (193). Ultimately, 3’UTR is the target of several RBP and/or antisense RNAs that regulate mRNA destiny. Modifications of mRNA 3’UTRs have thus important consequences on mRNA sub-cellular localization, translation efficiency or stability (194).

APA has been shown to also play an important role in innate immunity. For example, the sequencing of the human genome in 2001 helped to reveal that most TLRs have between two and four predicted APA sites (195). In line with this, several subsequent studies could underline that infected or inflamed macrophages display rapid and extensive changes in APA leading to a global shortening of 3’UTRs (155, 196). In LPS stimulated macrophages (BMDM and RAW 264.7 mouse cell line), this has been explained by the expression of Cstf-64, a 64kDa Cleavage stimulatory factor, that likely contributes to alternative polyadenylation of numerous genes associated with a global change in their expression (197). Similarly, macrophages derived from human blood monocytes infected by *Salmonella typhimurium* or *Listeria monocytogenes* show a near universal shift toward usage of more upstream polyadenylation sites, leading to shorter 3’UTR in genes where longer 3’UTR are targeted by miRNA negative regulation (155). Altogether, those observations argue for a role of 3’UTR shortening in the escape from immune repression, allowing a rapid establishment of innate immune responses. Consistent with this, infection of primary human monocyte derived macrophages and mouse peritoneal macrophages with VSV leads to a gradual shortening of 3’UTRs through the use of proximal cleavage/polyadenylation sites. From 2 to 16 hours post infection, mRNA displaying altered APA are enriched in immune-related Gene Ontology categories and this is accompanied by increased levels of several innate-related proteins such as RIG-1, RIPK1 (a kinase involved in host defense) (198) or DDX3Y (a RNA helicase involved in type I IFN production) (199). In line with this, down-regulation of different RBPs involved in 3’ mRNA processing prior to infection with VSV, promoted virus replication. Although it remains to be determined whether certain RBPs involved in 3’UTR processing are themselves regulated by APA and how viral infection modifies their expression/activity, this further validates the hypothesis about the important role of APA in regulating innate immunity (196). By contrast, Jia et al. study interestingly highlights a picture that is likely more sophisticated, where the impact of 3’ UTR shortening does not necessarily correlate with increased protein output. Indeed, while APA usage leads to stabilization and increased translation of several mRNAs, other immune-related mRNAs were negatively impacted by the viral induction of 3’UTR shortening, including Fos, SOS1 [a negative regulator of TLR signaling (200)], TNFRSF10D [a TRAIL-receptor with a truncated death domain (201)], CASP6, PPSB1, N4BP1 [a suppressor of cytokine response (202)], (196). Although more extensive analysis should be performed in relevant models, the impact of APA and 3’UTR shortening in the context of infection overall appears as an additional regulator of protein output with a putative role in the interplay between positive and negative regulators of innate responses.

## 5 Translational Control During Innate Immunity

### 5.1 mRNA Translation Process

Once matured, mRNAs are transported from the nucleus to the cytoplasm, where they can recruit specific initiation factors and ribosomes to undergo translation. The control of this step plays a critical role in most cellular processes as it provides a rapid response to endogenous and exogenous cues without requiring *de novo* transcription. Furthermore, translational control is versatile as it can be exerted on a global scale or restricted to specific mRNA species. The translation process itself can be split into four phases: initiation, elongation, termination and ribosome recycling. Translation initiation is commonly assumed to be the rate limiting and the most regulated step of the process. However, the advent of high-throughput sequencing and protocols such as ribosome profiling (203), which allows to map the position of individual ribosomes across all expressed transcripts at a single-nucleotide resolution, has uncovered many additional layers of regulation taking place during elongation and termination of translation. Altogether, mRNA translation can thus be regulated by RBPs through multiple mechanisms involving binding to either specific RNA sequences or structures found in the 5’UTR, coding sequence, 3’UTR or poly(A) tail of cellular and viral RNAs, or as a consequence of the detection of non-self RNAs.

### 5.2 Individual RBP-Mediated Translation Silencing

The nucleolysin TIA-1 (TIA1 Cytotoxic Granule Associated RNA Binding Protein) and its closely related homologue TIAR are both RBPs containing three RNA Recognition Motif (RRM) domains in their N-terminal (204–206). In response to stress-induced phosphorylation of the translation initiation factor eIF2*α*, these proteins participate in the assembly of membrane-less cytosolic structures called stress granules (SGs). In cooperation with other RBPs, TIA-1 binds and sequesters untranslated mRNAs into the SGs, away from ribosomes (207). This occurs in five steps: (i) phosphorylation of eIF2*α* results in abortive initiation complexes preventing ribosome elongation and resulting in the formation of 48S messenger RiboNucleoParticle (mRNPs); (ii) free 48S mRNP are aggregated by factors such as TIAR or TIA-1, initiating SG nucleation; (iii) secondary aggregation where mRNA transcripts bind to multiple proteins forming microscopically visible SGs; (iv) integration and signaling in which proteins that lack RNA-binding domains (RBDs), such as TIA-1 binding proteins (*e.g.* SRC3, FAST or PMR1), bind in a ‘piggyback’ manner proteins involved in SGs assembly; (v) mRNA triage: SGs are organized into compartments. In each compartment, transcripts are specifically selected for decay or stabilized for further export and integration into polysomes, or stored (208). In LPS-activated macrophages, it has been shown that TIA-1 and TIAR bind U-rich motifs of mRNAs and selectively induce the silencing of TNF-*α* translation, while other cytokines such as IL-1*β* or IL-6, are largely unaffected (209, 210). Similarly, activated macrophages from TIA-1–/– mice were shown to produce significantly more TNF-*α* as compared to macrophages from wild type mice (210). Although the direct link between TIA-dependent silencing of TNF-*α* and stress granules has not been formally shown in that cellular context, several lines of evidence suggest that TNF-*α* silencing is linked to a stress response. By contrast, stimulation of the integrated stress pathway, a cytoprotective response that regulates cellular homeostasis, can prevent the production of IL-1*β* in LPS-activated macrophages. Indeed, incubation of murine macrophages with Arsenic, a known inducer of eIF2*α* phosphorylation and stress granule formation, after LPS activation or bacterial infection, results in a decreased production of IL-1*β*. Mechanistically, this decrease is explained by the formation of stress granules, through the interaction of IL-1*β* mRNA with TIA-1/TIAR, that eventually leads to IL-1*β* mRNA degradation (211).

Altogether, these studies suggest that TIA-1 and TIAR constitute specific translational silencers regulating the cellular response to environmental stress. The fact that, depending on the environmental context, such a stress-response targets specific cytokine-encoding mRNAs, further suggests the existence of additional elements of specificity, as exampled in non-immune cellular context (212).

### 5.3 GAIT Complex-Mediated Translational Regulation

RBP-containing protein complexes such as the IFN-*γ*-activated inhibitor of translation (GAIT) complex play an important role in regulating transcript-specific translation during innate immunity (213).

**Figure 4:**
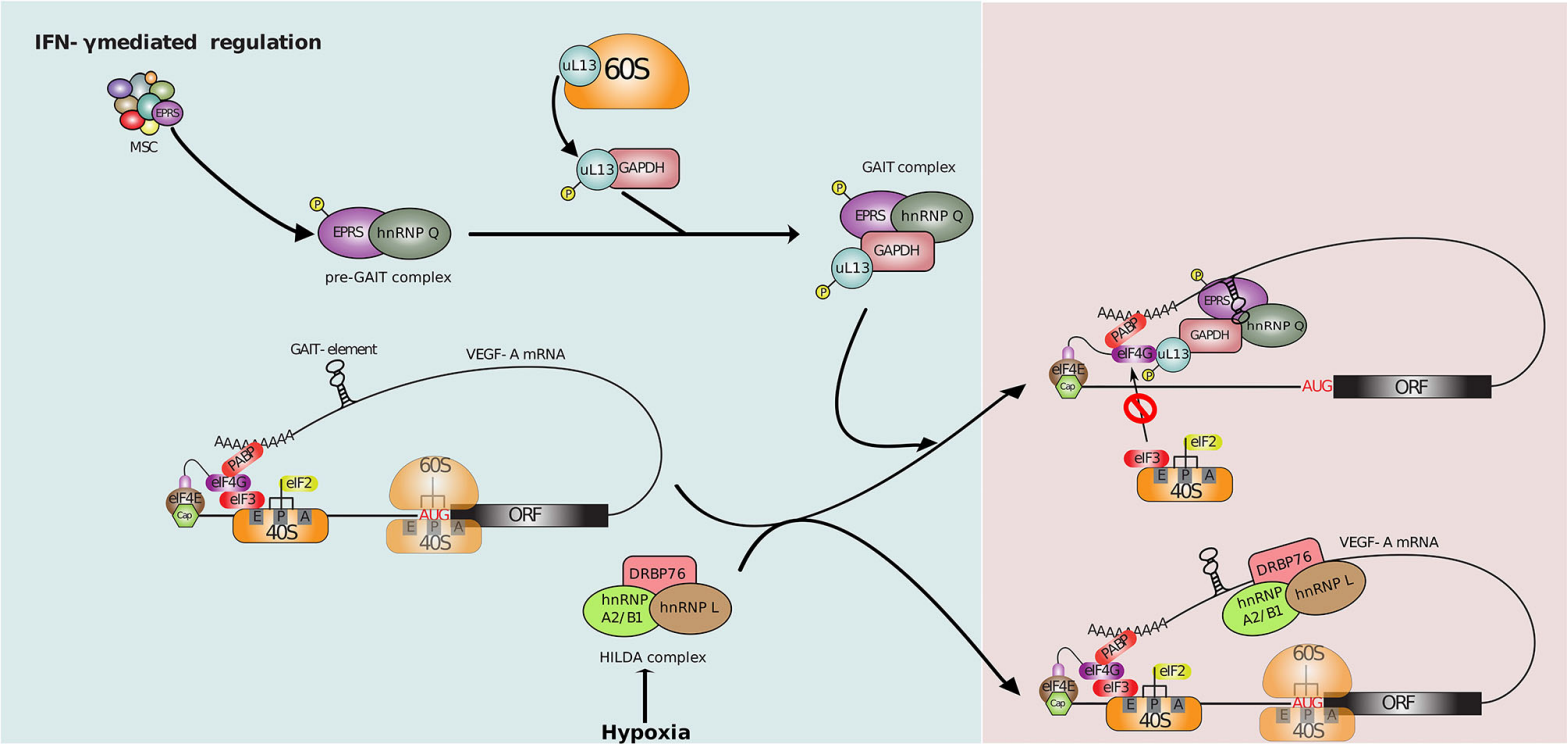
Translational control of VEGF-A mRNA. 1-8 hr post-IFN-*γ* induction, phosphorylation mediated release of EPRS from MSC followed by its association with hnRNP Q forms an inactive pre-GAIT (IFN-*γ* activated inhibitor of translation) complex. This complex joins uL13, released from the 60S ribosomal subunit, and GAPDH to form the heterotetrameric GAIT complex at 12-24hr. This functional GAIT complex suppresses the translation of many cellular transcripts including VEGF-A mRNA by blocking the interaction between eIF4G and eIF3 upon binding to a stem-loop RNA structure at 3’UTR called as GAIT element (213, 215). The hypoxic stimulus-based regulation: the binding of the HILDA (hypoxia-induced hnRNP L - DRBP76 - hnRNP A2/B1) complex adjacent to the GAIT element under hypoxia prevents the binding of the GAIT complex and restores VEGF-A translation (213, 215, 216).

GAIT is a heterotetrameric complex formed by the glutamyl-prolyl tRNA synthetase (known as EPRS), heterogeneous nuclear ribonucleoprotein Q (known as hnRNP Q or NSAP1), glyceraldehyde-3-phosphate dehydrogenase (GAPDH) and the ribosomal protein L13a [also known as uL13 in the new ribosomal protein naming system (214)]. Assembly of the GAIT complex in response to IFN-*γ* exposure occurs in two distinct stages that are temporally regulated (see **Figure 4**). The first stage, which occurs within 8 hours from IFN-*γ* exposure, is triggered by the phosphorylation of EPRS mediated by several kinases (*e.g.* CDK5, p35, mTORC1) (217, 218). This induces its release from the tRNA synthetase complex (MSC) and its interaction with hnRNP Q to form a ‘pre-GAIT complex’ that is not functional. The second stage occurs after 12-24h of IFN-*γ* exposure, when ribosomal protein uL13 is phosphorylated, and triggers its release from the 60S ribosomal subunit. uL13 then binds GAPDH and the ‘pre-GAIT complex’ formed by EPRS and hnRNP Q to generate a functional heterotetrameric GAIT complex (215, 219). Once functional, the GAIT complex becomes competent for binding transcripts containing specific RNA stem-loops in their 3’UTRs sequence (*i.e.* GAIT elements), that are present in numerous pro-inflammatory mRNAs. GAIT complex likewise represses their translation through a direct interaction between uL13 from the GAIT complex and the translation initiation factor eIF4G, which inhibits the association of eIF4G and eIF3 (217, 220). uL13 deficiency, however, does not impair ribosome assembly in general or its global translation capacity, highlighting a non-essential role for uL13 as a regulator of specific mRNA translation (221).

Macrophage specific knockout of uL13 in mice has shown that translational control driven by the GAIT complex is an important player in the resolution of inflammation (219, 222, 223). Indeed, many cellular transcripts involved in the inflammatory response contain GAIT elements in their 3’UTR and are translationally regulated by the GAIT complex following stimulation with IFN-*γ*. These include transcripts coding for chemokines, chemokine receptors (219) and cytokines (222).

Similarly, treatment of human monocytic U937 cell line with IFN-*γ* induces strong expression of vascular endothelial growth factor A (VEGF-A that promotes angiogenesis during inflammation) mRNA after 8 and 24 hours. However, while VEGF-A protein levels are increased 8 hours post IFN-*γ* treatment, its level returns to baseline at 24 hours. Indeed, VEGF-A translation is repressed by the GAIT complex *via* the binding of GAIT to its GAIT element (224). Conversely, VEGF-A mRNA is positively regulated by the HILDA ribonucleoprotein complex composed of the RBPs, hnRNP L, DRBP76 (or ILF3, a dsRBP), and hnRNP A2/B1 that promotes angiogenesis under hypoxia conditions (216, 225). Interestingly, the GAIT element located in the 3’UTR of VEGF-A is in vicinity of an RNA binding site for HILDA complex (**Figure 4**). Binding of the GAIT or HILDA complex is mutually exclusive and results in a conformational switch of the RNA that impedes binding of the other complex (216, 225). This conformational switch, dependent on normoxic or hypoxia cell condition, enables efficient VEGF-A regulation and tissue oxygenation following inflammation, through translational control. Altogether, this process highlight a translation-dependent mechanism by which monocytes/macrophages can handle conflicted clues in complex environment such as inflammation (216).

### 5.4 Viral RNA Translation Control

**Figure 5:**
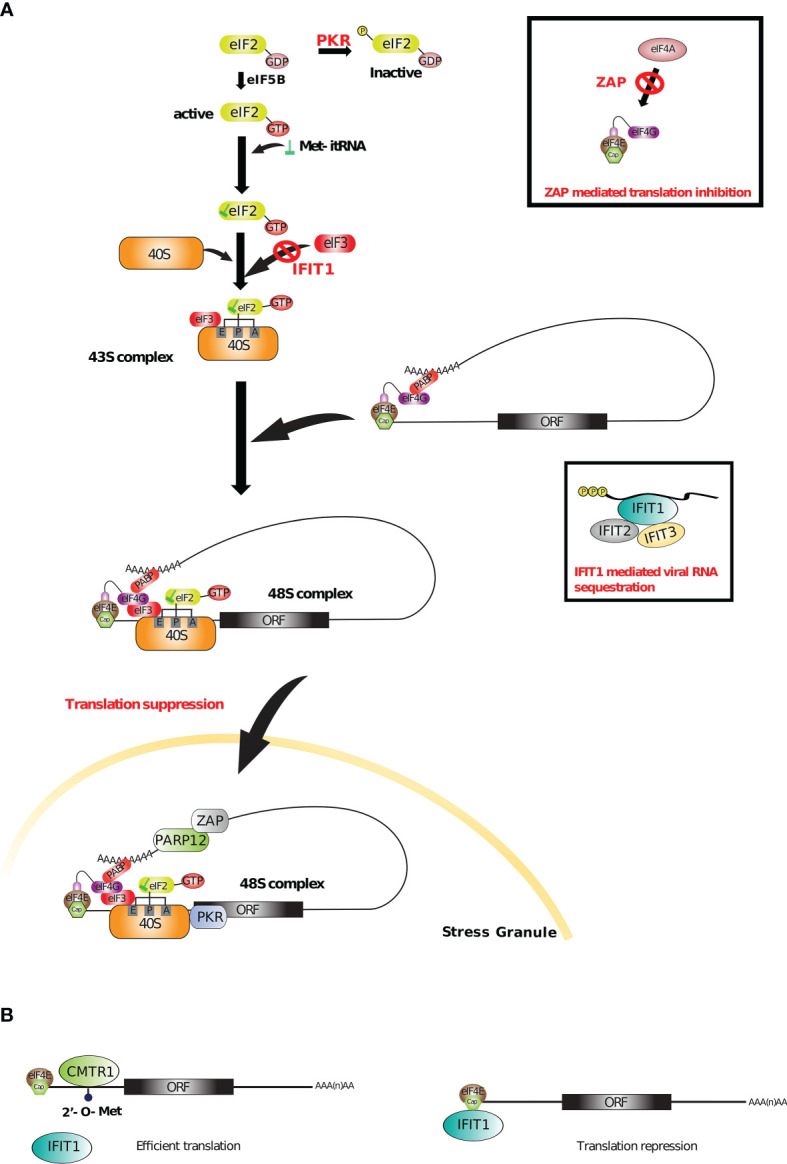
Translational control by ISG-RBPs. **(A)** Upon viral infection, several ISGs (such as PKR, ZAP and IFIT1) suppress viral and/or cellular translation by recognizing different RNA sequences or structures and targeting important initiation and elongation factors. With PARP12, PKR and ZAP also participate in stress granule mediated antiviral response (227, 230–232). **(B)** 2’O methylation of cellular mRNAs recruits CMTR1 that inhibits IFIT1 mediated translation repression (233, 234).

Many ISGs with RNA-binding capacity (*i.e.* ISG-RBPs) boost immune response by restricting viral replication through the regulation of mRNA translation (either self or foreign mRNA), some acting on bulk translation while other targeting specific transcripts (226, 227). Among these, the best characterized is the dsRNA-activated protein kinase (PKR). PKR contains two dsRBDs that recognizes dsRNA structures longer than 30 nucleotides through its N-terminal end, which are an abundant replication intermediate for RNA viruses (228). Binding of dsRNA by PKR induces homodimerization and autophosphorylation of PKR C-terminal kinase domain, leading to its activation. One major target of PKR is the translation initiation factor eIF2*α*, which becomes phosphorylated upon PKR activation. Phosphorylated eIF2*α* cannot be recycled and is no longer able to form a ternary complex with the initiating Met-tRNA and a molecule of GTP. This results in a global inhibition of translation initiation affecting both cap-dependent and most forms of IRES-dependent translation (IRES for internal ribosome entry site, an RNA element often located in 5’ UTR that allows translation initiation in a cap-independent manner). However, certain cellular mRNAs are selectively translated in the presence of high levels of phosphorylated eIF2*α* and many viruses are able to overcome this arrest (229). Additionally, PKR is also present in stress granules containing stalled 48S ribosomes (**Figure 5A**). The activation of the stress granule localized PKR contributes to amplifying the innate immunity without the need for viral dsRNA pattern recognition (231) highlighting another antiviral mechanism of PKR. In line with this, PARP12, an ISG-RBP phosphorylated by PKR, has been shown to localize into SGs and p62/SQSTM1 containing structures (an adaptor protein involved in innate signaling and autophagy). Regulated by type I IFN during LPS stimulation, PARP12 contributes to the cellular antiviral response by increasing the SG-mediated translational silencing of viral and cellular RNAs. PARP12 contains five Cys-Cys-Cys-His (CCCH) zinc finger domains, the N-terminal one being essential for its subcellular location and function (235, 236). ISG20, another ISG-RBP, contains an RNase I domain and displays antiviral activities (237). ISG20 is upregulated by the three types of IFN and appears to perturb both viral mRNA translation and stability either directly, or *via* host factors (238). At the opposite of PARP12, ISG20 has been shown to specifically inhibit translation of a large number of non-self RNAs but not that of host mRNAs, participating in the discrimination between self and non-self substrates, however its mechanism of action still remains elusive (239). This is also the case for ZAP, a zinc-finger antiviral protein, also known as ZC3HAV1 that promotes translational repression of spliced viral mRNAs, by binding specific ZAP responsive element present in target viral RNAs. Once bound to viral RNAs, ZAP disrupts the interaction between the translational initiation factors (eIF4G, eIF4A) and the viral mRNAs, leading to their translational silencing (240, 241). ZAP also participates in maintaining the integrity of stress granules which could potentially be linked to its ability to restrict virus infection (230). More recently, the long isoform of ZAP has been shown to be essential for limiting translation of viral RNAs (242). At the opposite of the short isoform, the long isoform of ZAP contains a PARP domain and a CaaX motif (amino acids “CVIS”) at its C-terminus. Because ZAP is known to lead to degradation or translational inhibition by binding CpG dinucleotides in viral RNAs (243), CpG-enriched viruses have been used to highlight the antiviral activity of ZAP RBP. First, it has been shown that not only the N-terminal RBD, but also the C-terminal PARP domain both contribute to the restriction of CpG-enriched HIV-1. Second, the presence of the well-conserved CVIS sequence of the CaaX box mediates S-farnesylation (addition of a hydrophobic group). This post-transcriptional modification combined with the presence of the PARP domain are required for a full antiviral activity, through the recruitment of important co-factors such as TRIM25 and KHNYN proteins and the localization of ZAP into intracellular membranes. The subcellular distribution of this RBP has been shown to be critical for the antiviral restriction of both CpG-enriched HIV-1 and SARS-CoV-2 viruses (242).

RBPs can also recognize specific RNA patterns carried by some viruses in order to restrict their translation. For example, IFIT1 (interferon-induced protein with tetratricopeptide repeats 1), induced by IFN-*α*/*β* upon viral infection, binds a triphosphate group on the 5’ terminal of viral RNAs (PPP-RNA) (244) in a sequence-independent manner and form a complex with IFIT2 and IFIT3 (and other proteins from IFIT family) to physically sequester the viral RNA and limit the assembly of viral particles (see **Figure 5A**) (245, 246). IFIT1 also interacts with eIF3 thereby blocking its association with the ternary complex (eIF2-GTP-Met-itRNA) to further inhibit translation of viral RNAs (247). In addition, the lack of 2’-O methylation of viral RNAs increases the interaction with IFIT1 and therefore raises the translational silencing (248). However, host mRNAs lacking 2’-O-methylation can also be targeted by IFIT1-mediated silencing. Interestingly, one way to circumvent this issue is mediated by CMTR1, another ISG also known as ISG95. CMTR1 is responsible for the catalysis of 2’O-methylation, which prevents IFIT1-mediated repression, especially for some ISG transcripts (see **Figure 5B**) (233). By doing so, CMTR1 promotes ISG protein expression in response to type I IFN. This example underlines the complex relation between (viral and host) RNA and RBPs and the requirement of several layers of regulation to optimize the antiviral response induced by the infection.

Altogether, a broad panel of RBPs exists, that regulate RNA dynamics through the modulation of translation. Their molecular and functional structure are divergent but they all converge into shaping the intensity and the efficiency of the innate immune response in time and space.

## 6 mRNA Stability

### 6.1 mRNA Decay Process

The number of proteins synthesized from any given mRNA molecule is defined by its translation and degradation rates (249, 250). In eukaryotes, for most cellular transcripts, decay involves deadenylation and/or decapping. Deadenylation of the mRNA 3’ end is mainly dependent on the CCR4-NOT complex among other deadenylases (251, 252), while decapping at the 5’ end is performed by the mRNA decapping complex Dcp2-Dcp1 (253, 254). Each of these events is followed by exonucleolytic degradation from one or the other transcript extremity: from 5’ to 3’ by the exonuclease Xrn1 (255) and from 3’ to 5’ by the RNA exosome complex (256). Degradation of mRNAs by decapping complexes is thought to occur in cytoplasmic processing bodies (P-bodies) that are cytosolic membraneless structures composed of aggregates of proteins involved in RNA metabolism (including the decapping complex and Xrn1 among many others) and untranslated mRNAs (257). However, this model has been challenged in the past few years by studies indicating that P-bodies can be sites of mRNA storage and “triage” before resuming translation or being degraded (258, 259). Furthermore, growing evidence points to a close link between mRNA translation and degradation, which can occur simultaneously, outside of P-bodies (249, 260–263). Finally, endonuclease-associated mRNA decay can also occur, initiated by internal cleavage and followed by bidirectional exonuclease degradation (264). However this mechanism is not involved in bulk mRNA degradation and is usually restricted to a subset of mRNAs with specific features.

RNA-seq technology combined with metabolic labeling of nascent mRNA transcripts allows to measure transcriptome-wide mRNA degradation rates in different conditions (*e.g.* LPS, TNF-*α* in myeloid cells or fibroblast) (265–267). Even in the absence of metabolic labeling, recent mathematical models are able to estimate with accuracy mRNA degradation rates from total RNA-seq datasets (268, 269). These analyses showed that in cells stimulated with LPS or TNF-*α*, the raise of mRNA levels induced by pro-inflammatory stimuli is mainly due to a global increase at the transcriptional level, with a globally constant mRNA degradation rate (265, 266). However, following LPS stimulation of DCs, a small set of mRNA show a rapid increase in their degradation rate, following an initial increase in their translation, thus affecting their cellular level within the first 3 hours after stimulation. Interestingly, most of the concerned mRNA were immediate-early genes (e.g. Fos, Jun, Egr1, Tristetraprolin) suggesting that, in the context of a rapid and transient response, the rate of the mRNA decay is an important parameter controlling mRNA output (266).

Rapid mRNA degradation mechanisms are essential for shaping the innate immune responses and the binding by RBPs to transcripts happens in a sequence or structure dependent manner. The most widely targeted cis-elements are AU-rich elements (AREs), with RBPs stimulating the deadenylation of mRNA (270).

### 6.2 ARE-Mediated Regulation

ARE usually consists in several clusters of the AUUUA pentamer or UUAUUUA(U/A)(U/A) nonamer sequence located in the 3’UTR of protein-coding transcripts (271). Their sequence is specifically recognized by ARE-binding proteins that can compete against each-other for ARE binding and thus, depending on the relative expression of ARE-binding proteins as well as the nucleotide context in proximity of a given ARE, these elements can either lead to transcript destabilization (272), translational control (273) or stabilization (274) [for a review, see (275)]. Historically, AU-rich elements were discovered as cis-acting elements responsible for inducing mRNA degradation of transcripts coding for inflammatory mediators (276). Indeed, it has been shown that early and transient mRNA transcripts induced after LPS or TNF-α stimulation in macrophages are enriched in AREs in their 3’UTRs, which is in line with the essential control of the immune response duration by rapid mRNA decay (277, 278). Consequently, ARE-mediated regulation affects many pro- or anti-inflammatory cytokines such as IL−2, TNF-*α*, IL−1*β* or Granulocyte Macrophages Colony Stimulating Factor (GM−CSF) (279). Their AU-rich sequence are recognized by over 20 different ARE-binding proteins with different roles in regulating mRNA metabolism (24, 279).

**Figure 6:**
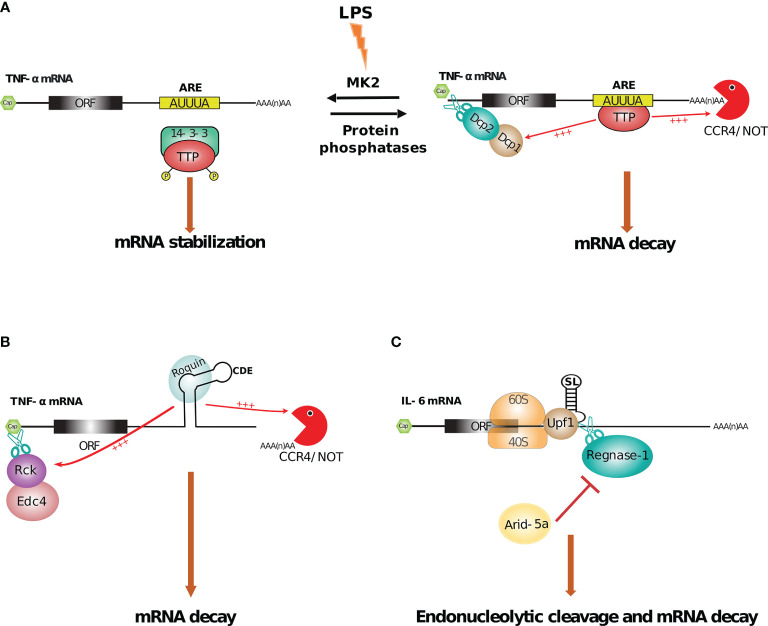
RBPs mediated regulation of mRNA stability. **(A)** TTP facilitates TNF-*α* mRNA decay by binding to an AU-rich element (ARE) followed by recruitment of decapping enzymes DCP1/2 and CCR4-NOT deadenylating complex at 5’ and 3’end respectively. Conversely, in mice BMDM, LPS mediated activation of MK2 kinase suppresses this process by phosphorylating TTP followed by its sequestration by 14-3-3 protein, which results in TNF-*α* mRNA stabilization (287–289). **(B, C)** Roquin and Regnase-1 promote decay of several inflammatory mRNAs by binding to stem-loop structures present near the 3’end. For example, Roquin facilitates deadenylation *via* CCR4-NOT complex and decapping *via* Rck/Edc4 by binding to a constitutive decay element (CDE) located in the 3’UTR of TNF-*α* mRNA. Whereas, Regnase-1, in cooperation with Upf1, leads to endonucleolytic cleavage of IL-6 mRNA by recognizing a specific stem-loop (SL). This process is inhibited by Arid-5a protein (64, 290, 291).

One of the most well-known examples of ARE binding proteins involved in inflammatory process is Tristetraprolin (TTP, a Cys-Cys-Cys-His (CCCH) zinc finger protein). It has been identified in various organisms from human to yeast (280–282) and has been shown to bind to the ARE contained within the 3’UTR of targeted mRNAs *via* its zinc finger domain. Well known targets are mRNAs displaying high turnover rates such as cytokines and growth factors (283–285). Mechanistically, TTP recruits the CNOT1 subunit of the CCR4-NOT complex (286), leading to deadenylation which accelerates degradation of the target mRNA. In addition to inducing deadenylation, TTP has also been described to stimulate mRNA-decay by decapping through the involvement of decapping proteins (Dcp1/2) (see **Figure 6A**) (292). TTP is therefore highly controlled to maintain a proper innate response intensity and duration. In mouse BMDMs (Bone Marrow-Derived macrophages), TTP expression is ubiquitous and low in resting conditions but, during the first hours of inflammation, TTP is phosphorylated by MK2 and further sequestered by 14-3-3 proteins (293, 294), therefore leading to pro-inflammatory cytokines mRNAs stabilization and accumulation (see **Figure 6A**) (295). Following this, TTP expression is empowered both transcriptionally and post-transcriptionally by inflammatory stimuli [as described upon activation of TLR4 (296, 297)] leading to the destabilization of several inflammation-associated mRNAs, such as TNF-*α* (283, 298, 299). By alternating between sequestration and release, TTP is able to regulate the stability of important inflammation-related mRNAs in a temporal manner.

### 6.3 Non ARE-Mediated Regulation

Non ARE-mediated regulation refers to mRNA regulation (decay or stabilization) by RBPs, through the recognition of specific sequences and RNA structures in the 3’UTR of targeted mRNAs, that are not ARE. For example, Constitutive Decay Element (CDE) relates to a decay through deadenylation, that is mediated by a 3’UTR conserved sequence with a stem-loop structure specifically recognized by Roquin and Roquin2 (300–302).

Non ARE-mediated regulation thus further regulates RNA stability of pro- and anti-inflammatory elements. For example, in addition to an ARE element in its 3’UTR, TNF-*α* encoding mRNA contains a non-ARE CDE that tightly regulate its expression, and overall prevent excessive TNF-*α* production in inflamed macrophages (300). Following macrophages (mouse RAW 264.7 cell line and BMDMs) stimulation by LPS, the CDE (which corresponds to a conserved 37 nucleotide long RNA stem-loop structure) is recognized by Roquin and Roquin2 (see **Figure 6B**). Roquin proteins actively recruit the CCR4-Caf1-Not deadenylase complex (a major multi-subunit complex responsible for the deadenylation of a large number of eukaryotic transcripts) through their C-terminal domain, which mediates deadenylation of the TNF-*α* mRNA and its accelerated clearance (301). Within this complex, Caf1a was identified as the factor directly responsible for deadenylation, since expression of a dominant-negative mutant of Caf1a, completely abolished Roquin mediated mRNA decay of CDE-containing mRNAs (301). The binding of a decapping enzyme Edc 4 (enhancer of mRNA decapping protein 4) and the RNA helicase Rckat 5’end also contributes to this mRNA degradation process. In addition to TNF-*α* transcripts, conserved CDEs were identified in more than 50 other cellular transcripts, enriched in T cell differentiation, nucleic acids metabolism and transcription factor functions, suggesting a wider role of Roquin proteins and their CDE target elements in modulating immune responses (301). In addition to AREs and CDEs, embryo deadenylation element (EDEN)-like sequences (rich in uridine–purine dinucleotides) present in immune related transcripts such as TNF-*α* and c-fos where they are scattered throughout the mRNA, are recognized by CUG-BP1 (CUG triplet repeat RNA-binding protein 1, also known as CELF1). In this case, CUG-BP1 is able to directly recruit the PARN deadenylase to induce target mRNA decay (303). Multiple mRNA decay pathways implicating different cis-acting RNA elements, specific adaptor proteins and leading to the recruitment of different effector proteins or complexes are responsible for modulating the stability of hundreds of transcripts during the inflammatory response. Some transcripts, such as those coding for TNF-*α* contain multiple different cis-acting RNA elements responsible for inducing mRNA destabilisation, allowing for complex regulatory networks responding to multiple inputs.

### 6.4 Translation-Dependent mRNA Decay

mRNA degradation can be strongly interconnected to other processes such as translation. For example, mRNA quality control pathways, such as nonsense-mediated decay (NMD), no-go decay (NGD) and non-stop decay (NSD) also rely on translation to induce degradation of aberrant mRNAs [for a review see (263)]. However, accumulating evidence indicates that functional mRNAs can be degraded co-translationally, and therefore the rate of translation can influence their degradation rate (262). Other mRNA degradation pathways such as the one mediated by microRNAs have been shown to occur co-translationally and in some cases to depend on target mRNA translation to trigger degradation (304–306). Finally, some target-specific mRNA degradation pathways driven by RBPs have been shown to require mRNA translation in order to license for degradation.

This is illustrated by Regnase-1 (also known as MCPIP1 or ZC3H12A), a RBP recognizing specific stem loop in 3’UTR and harboring an endoribonucleolytic activity thought to act as a negative regulator of pro-inflammatory processes (**Figure 6C**). Regnase-1-deficient mice develop severe immune disorder and mostly die around 12 weeks old (307) [for an extensive review, see (308)]. In peritoneal macrophages from mice deficient for Regnase-1, TLR-stimulation induces increased levels of IL-6 and IL-12p40 secretion, while TNF-*α* remains unaffected (307). In line with this, stimulation of human monocytes derived macrophages with IL-1*β* induces Regnase-1 expression that will, in turn, shorten IL-1*β* mRNA half life (309). In both studies, the function of Regnase-1 has been linked to its ability to bind RNA stem-loops in the 3’UTR of target mRNAs and induce their rapid degradation through a putative amino-terminal nuclease domain (290). Interestingly, Regnase-1 not only plays its role of anti-inflammatory regulator in myeloid cells, but also in epithelial cells by exerting RNase activity towards the IL-8 mRNA, that stimulates immune cell migration and phagocytosis (310). More recently, Mino et al. could show that Regnase-1 mediated decay occurred in a translation-dependent manner. In this process, the stem-loop RNA structure is first recognized by Regnase-1 prior to the pioneer round of translation. However, this interaction alone is not sufficient for Regnase-1 to induce mRNA cleavage and decay. Instead, during the first round of mRNA translation, translation termination recruits the RNA helicase Upf1 to Regnase-1 which stimulates its RNA-helicase activity leading to unwinding of the stem-loop structure bound by Regnase-1 and allowing target mRNA cleavage (311). Remarkably, Regnase-1 and Roquin share multiple target sites in several transcripts, although not all [as shown for TNF-*α* whose decay depend on Roquin but not Regnase-1 (301, 307)]. This observation led to the conclusion that Regnase-1 and Roquin could, to some extent, act in concert for a spatio-temporal regulation of common immune-related genes. Indeed, Regnase-1 mediated mRNA decay occurs in the ribosome/endoplasmic reticulum and is translation-dependent, likely playing a role during the early acute phase of immune response. By contrast, Roquin is mainly localized within stress granule, targeting translationally inactive mRNA, in line with a role during the late phase of immune response (290).

MicroRNAs (miRNAs or miR) are other important modulators of mRNA decay and translation by interacting with the 3’UTR of the target transcripts. miRNAs are short (22 to 25 nt long) noncoding RNAs that regulate gene expression in numerous cellular processes. By targeting the 3’UTR of protein coding transcripts, they might directly regulate expression of about 60% of all mammalian genes (312, 313). miRNAs act by guiding the RNA-induced silencing complex (RISC) to interact with mRNAs, inducing translational repression followed by mRNA decay (314). miRNAs have been shown to be critical regulators of immune responses (315, 316). For instance, in hepatocytes, miR-122 is able to repress expression of several kinases involved in STAT3 phosphorylation and promote antiviral immunity by repressing STAT3 signaling pathway (317). Similarly, miR-155 is an important regulator of the innate and adaptive immune response. Its expression is induced in response to pathogen infection and several inflammatory stimuli, and repressed in response to anti-inflammatory cytokines (316, 318). Finally, it has been shown that ISGs have more conserved miRNA target sites in their 3’UTRs than all other cellular mRNAs and are therefore more prone to regulation by the RISC complex (319). Interestingly, infection with viruses or synthetic ligands that activate an antiviral response result in a global inhibition of RISC that removes the negative effect of miRNAs on ISG transcripts to improve their expression and potentiate the antiviral response (319). Furthermore, in some contexts such as during Poxvirus infection, the host miRNA activity is ablated by the viral machinery to avoid direct translation silencing of its own transcripts (320). This action probably outweighs the costs of any possible increase of ISG toxicity. In addition to a direct role of miRNAs in the cell in which they were produced, horizontal transfer of miRNAs through specific vesicles such as exosomes might be a key factor in inflammatory response (321). For example, after binge or chronic alcohol consumption, the number of exosomes containing miR-122 drastically increases in circulation. They are transferred from hepatocytes to monocytes, sensitizing them to the inflammatory response (322). Similarly, alcohol-exposed monocytes communicate with naive monocytes *via* the release of extracellular vesicles containing high levels of miR-27a. These miR-27a cargos lead naive monocytes to differentiate into M2 macrophages (an alternative group of macrophages) (323). Furthermore, macrophages are not the only cell-type sensitive to horizontal transfer of miRNAs during innate responses. This process can also occurs between DCs (324) or between T cells and DCs (325). miRNAs, by binding RNAs, regulating their expression, and being the communication support between immune cells, participate in modulating immune response in quantitative and qualitative ways.

### 6.5 Stabilization of RNA

Some RBPs, instead of promoting mRNA degradation, increase the stability of both cellular and viral mRNA, either by direct competition for mRNA binding with RBPs involved in mRNA degradation, or indirectly through the regulation of factors involved in mRNA decay. For example, the RBP Arid-5a is exported from the nucleus to the cytoplasm during LPS-mediated macrophage activation. Cytoplasmic Arid-5a thus promotes mRNA stabilization of cytokines such as IL-6, by suppressing the function of Upf1, which is essential for Regnase-1-mediated mRNA decay function (see **Figure 6C**) (326). Notably, these cytokines (*e.g.* IL-6 mRNA) can also be stabilized through the inactivation of miRNAs. Indeed, IL-6 mRNA can be targeted by miR-26 family members for degradation. However, miR-26 family members can be inactivated by TUT4 (Terminal uridylyltransferase 4, also called zcchc11), a ribonucleotidyltransferase (327) which adds uridine residues to the 3’ ends of miRNAs, thereby inactivating them. Inactivation of miR-26, that act as inhibitors of IL-6 translation and, thus indirectly promoting IL-6 mRNA stability (328).

Another example of RBP-mediated stability relates to HuR, an ubiquitously expressed RNA binding protein, (also known as ELAV-like protein 1), which is known as one of the most important AU-rich element (ARE)-containing mRNA stabilizing proteins (329). This RBP contains three RNA recognition motifs (RRMs) called RRM1-to-3. RRM1 & RRM2 domains bind to U/AU-rich RNA (330), whereas the RRM3 domain is able to interact with poly(A) tails of HuR’s mRNA targets (331). HuR positively regulates antiviral responses by stabilizing diverse mRNAs in response to RLR or cGAS stimulation, including IFN-*β* (332), ISGs and regulators of host defense mechanism (332, 333). For example, following RLR stimulation, Polo-like kinase 2 (PLK2), regulates the nuclear translocation of IRF3. HuR has been found to stabilize the mRNA coding for PLK2 (334), increasing PLK2 levels and therefore assisting IRF3 transit into the nucleus in order to activate the transcription of IFNs and ISGs [for extensive review about IRF, see (335)]. Interestingly, HuR mode of action appears to be closely linked to its subcellular localization, that itself depends on the cell type, and the cellular context (*e.g.* naive versus activated cells). Initial studies performed in HEK293 cells, by combining data from different experimental systems such as PAR-CLIP, and whole-transcript expression profiling, have shown that HuR is involved in coupling pre-mRNA processing and mRNA stability, highlighting an important role of HuR within the nucleus through its binding at intronic sequences (336). However, further analysis using THP-1 cells (that, in their naive state, shared HuR properties observed in HEK293 cells) revealed that HuR dramatically modifies its binding properties upon activation. Indeed, cGAMP stimulation was associated with an accumulation of HuR in the cytoplasm, and a concomitant enrichment in cellular transcript 3’UTR binding (333). Those results underline a dynamic network in which RBP tightly regulate the complex changes that occur during immune activation.

RBP-mediated RNA stabilization is also important in the mechanism of action of specific therapeutic regiments or, by contrast, during viral immune escape. For example, under DNA-methyltransferase inhibitors (DNMTis) cancer therapy, myelodysplastic syndrome (MDS) or acute myeloid leukemia (AML) patients are treated with decitabine or azacitidine. This induces the expression of dsRNAs from endogenous retroviruses (ERVs), that are normally silenced by epigenetic regulation. ERVs are in turn recognized by a specific PRR, MDA5, that stimulates an immune response, leading to the death of the cancer cell. Concomitantly, STAU1, which contains multiple dsRBDs, has been shown to cooperate with a long non coding RNA, TINCR, to binds ERV transcripts. The STAU1-TINCR-ERV complex stabilizes ERV transcripts and is required to promote the expected immune response and cell death (337). Therefore, by mediating the efficiency of the DNMTis treatments, levels of STAU1 and TINCR are important indicators of patient receiving DNMTis treatment. Following the same principle, STAU1 has been found to bind and stabilize infectious bursal disease virus (IBDV) dsRNAs, although with a different outcome. Indeed, as opposed to the previous example, binding of STAU1 allows IBDV to escape its recognition by MDA5 and favoring IBDV replication and escape from host antiviral response (*i.e.* IFN response) (338).

In combination, all these RBPs participate in a regulatory network that finely tune inflammation by controlling the fate of diverse mRNAs. RBPs deploy mRNA decay and/or stability mechanisms to maintain immune homeostasis. This is allowed through a timely regulation of RBP activity during immune responses. Indeed, RBPs participate in the positive regulation of rapid pro-inflammatory processes at the onset of immune response and, conversely, appear to play an even more important role in shutting down or reducing inflammation at later time points, thus preventing detrimental tissue damage. Interestingly, this tight regulation appears often regulated by the subcellular localization of RBPs, with RBP harboring different function depending on their local interacting environment. As the mechanisms behind these interactions are still not fully characterized, it will be important to further investigate the different roles and molecular mechanisms by which RBPs regulate mRNA stability during the course of innate immune responses.

## 7 Epitranscriptomics

**Figure 7:**
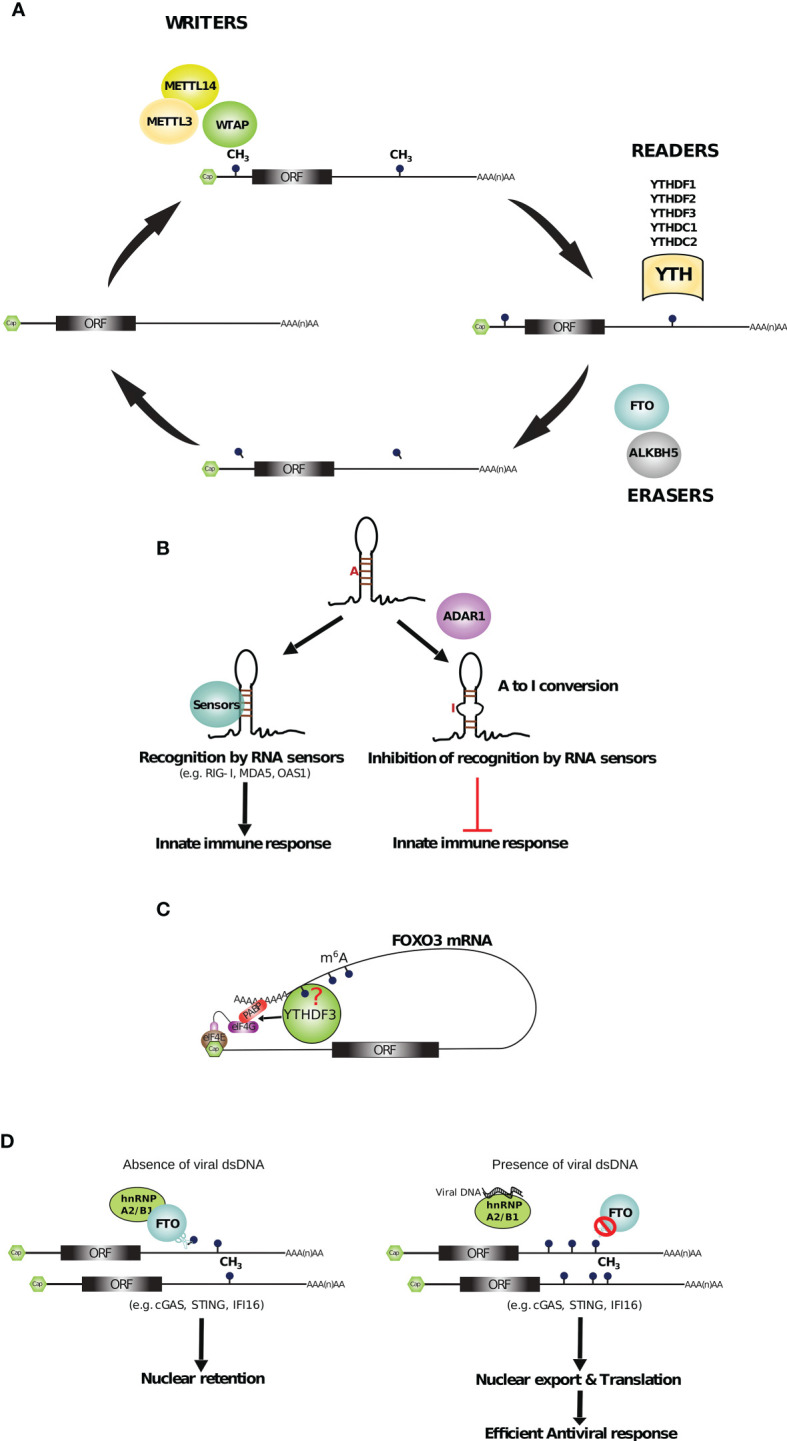
RBPs mediated epitranscriptomic regulation of innate immunity. **(A)** m^6^A modifications, elicited by methyltransferases (METTL3, METTL14 and WTAP), are read by YTH domain-containing proteins (YTHDF1-3, YTHDC 1-2) and reversed by the erasers (FTO and ALKBH5) to dynamically control the gene expression at the post-transcriptional level (349–351). **(B)** Recognition of endogenous dsRNAs by RNA sensors (e.g. RIG-I, MDA5 and OAS1) induces an innate immune response. However, A-to-I mediated base editing mediated by adenosine deaminase ADAR1 inhibits this process (352–354). **(C)** YTHDF3 promotes FOXO3 mRNA translation by binding the 5’UTR and circularizing the mRNA. The requirement of m^6^A for YTHDF3 recruitment has not been elucidated yet (355). **(D)** On the left, hnRNPA2B1 interacts with FTO, a m^6^A demethylase, to remove the m^6^A modifications from hnRNPA2B1 mRNA targets. Thus, these mRNAs, important for the antiviral response, are retained into the nucleus. On the right, when viral DNA is sensed by hnRNPA2B1, the interaction with FTO is disrupted and hnRNPA2B1 targets, containing more m^6^A modifications, are exported to the cytoplasm to be translated and to activate an efficient antiviral response (356, 357).

In analogy to the epigenetics field, epitranscriptomics involves a biochemical modification of the ribonucleotide sequence. This field of RNA modification has been recently recognized as an important layer in post-transcriptional regulation of gene expression. The source of this emergence comes from the technical advances in the detection and mapping of chemically modified bases (339, 340). Transfer RNA (tRNAs) are the most modified RNA species, with up to 25% of their bases being post-transcriptionally modified (341, 342). tRNAs but also rRNA, snRNA, lncRNA, miRNA or mRNA, among others, can bear such modifications (341–343). These modifications can be simple methylations but also complex multistep transformation with incorporation of low-molecular-weight metabolites (341). A large number of modifications have thus been identified in coding and non-coding RNAs. While m^6^A is the most common mRNA reversible post-transcriptional modification (344), alternative reversible methylation can also occur on the carbon of the fifth position on cytosine (5-methylcytidine or m^5^C) or on the nitrogen of the first position on adenine (N^1^-methyladenosine or m^1^A). Other types of RNA modifications such as Cytidine-to-Uracil or Adenine-to-Inosine RNA editing (C-to-U or A-to-I, respectively) can lead to changes in the secondary structure of the edited RNA (345) and in the protein sequence encoded by the mRNAs in case of editing events taking place within the coding sequence (346). Pseudouridine, a C5-glycoside isomer of uridine, is necessary to support proper secondary and tertiary structures of rRNAs and tRNAs, thus affecting mRNA translation (347). Mechanistically, most of these RNA modifications involve writer and reader proteins. Others, such as m^6^A and m^1^A, can involve additional eraser proteins allowing reversible and dynamic modifications (348). The writers are the RNA-modifying enzymes able to catalyze the transfer of the chemical group on the ribonucleotide targets (*e.g.* methyltransferase), while the eraser proteins can reverse the modifications by specifically removing the chemical groups from the RNA targets. Finally, the readers are the RNA-binding proteins able to specifically bind the RNA bases bearing the chemical modification (see **Figure 7A**) (342, 358).

It is now well recognized that epitranscriptomics affects several molecular processes. In the immune system, while epitranscriptomics appears important during hematopoietic stem cell differentiation (359), it is currently viewed as a major mechanism that allows self- and non-self dsRNAs discrimination during innate responses (348, 360). Indeed, while dsRNA is a feature found in numerous viruses, endogenous dsRNAs are also found in healthy cells, originating from transcription of endogenous retroviruses, mitochondrial transcripts, or inverted-Alu repeat sequences (353, 361). This raises an important challenge for innate receptors (*e.g.* RIG-I or MDA5), since they need to discriminate between self and non-self dsRNA molecules. While evolutionary elimination of dsRNA sequences within cellular mRNAs has been observed, which should limit innate immune activation against host RNAs, dsRNAs remain frequent in pre-mRNAs (362). One way to circumvent this issue is through modification of dsRNAs in order to prevent their recognition by PRRs and downstream type I IFN responses. Such a mechanism is well illustrated by the Aicardi Goutieres Syndrom, an inflammatory disorder affecting mainly the brain and the skin and associated with aberrant type I IFN production. Aicardi Goutieres Syndrom is associated with mutations in ADAR1, a dsRNA specific adenosine deaminase responsible for the most common cellular RNA editing through hydrolytic deanylation of Adenosine to Inosine (A-I editing) (363, 364). In line with this, ADAR1 deficient human monocytes derived macrophages leads to increased RLR signaling and pro-inflammatory cytokines (*e.g.* IFNs, IL-1, IL-6) production.

Through A-I editing, ADAR1 appears to modify host dsRNAs, preventing their recognition by dsRNA innate sensors. Accordingly, mutant mice carrying an ADAR1 protein that is editing deficient (ADAR1^*E*861^ *^A/E^* ^861^ *^A^*) are embryonically lethal, and present over-activation of IFNs and dsRNA-sensing pathways. This deregulated innate responses are due to a lack of A-I editing in ADAR1 ADAR1 ^*E*861^ *^A/E^* ^861^ *^A^* embryos, as further shown by a decrease in A-I editing in a vast majority of RNA targets. Moreover, the phenotype of ADAR1 ADAR1^*E*861^ *^A/E^* ^861^ *^A^* can be rescued by a concurrent deletion of MDA5 (352). Similarly ADAR1-/- mice are embryonically lethal and this phenotype can be rescued by crossing ADAR1-/- to MAVS-/- mice (348). In those double knock-out mice, the aberrant type I IFN response induced by ADAR deficiency is prevented by inhibiting the RLR pathway (348, 352). Likewise, ADAR1 deficiency confers A549 human lung epithelial cell lines with a lethal phenotype and MAVS ablation partially restores ADAR1-/- cells’ survival. However, in this cell type, full rescue of ADAR1-/- lethality is obtained by an additional ablation of RNase L, an ISG induced upon dsRNA sensing that leads to translation arrest by cleaving rRNAs and mRNAs (see section *RNA Sensing and Regulation of PRR Activity by Non-PRR RBPs*). ADAR1 appears as an essential protein in protecting host dsRNA from innate recognition, thus preventing aberrant innate responses against self (see **Figure 7B**) (348).

Altogether, these results led to the elegant hypothesis that editing of host RNAs by ADAR1 could prevent their recognition by innate sensors (*e.g.* RLR, RNase L) while viral RNAs, which are not edited, would trigger robust innate responses (348). However, in some contexts of infection, several studies have described ADAR1 as a proviral factor. This is notably the case following infection with Vesicular Stomatitis Virus (VSV) (365), Zika virus (366), HIV (367), HCV (364) or Measles Virus (368). One major mechanism by which ADAR1 promotes viral replication is through inhibition of PKR. As mentioned earlier (section *Viral RNA Translation Control*), PKR is an IFN-inducible protein that, following dsRNA recognition, phosphorylates eIF2*α*, favors the formation of stress granules, and acts as a major inhibitor of global translation (for both cellular and viral mRNAs). ADAR1 mediated inhibition of PKR occurs in a editing-dependent or -independent way, and mostly requires direct interactions between ADAR1 and PKR (353, 369). ADAR1 is thus seen as an important inhibitor of self-recognition in homeostatic conditions, to prevent auto-immune disorder, but paradoxically might, in some context, favor viral replication following infection. Finally, two distinct isoforms have recently been described for ADAR1, a cytoplasmic isoform (p150) and a nuclear one (p110), endowed with pro- and anti-viral properties, respectively (370). This suggest additional levels for ADAR1 regulation, and further studies will be required to fully comprehend the overall impact of ADAR1 activity in homeostatic condition or following infection.

Other RNA modification mechanisms have been described, that allow induction of innate responses specifically towards viral RNAs, while protecting host RNA recognition by innate sensors. For example, PKR is able to recognize short stem loops in a 5’ triphosphate dependent manner, knowing that 5’ triphosphate are mostly present in viral or bacterial transcript (369). Likewise in DCs, RNA from bacteria that is devoid of nucleoside modifications, induces strong activation of TLR3, TLR7 and TLR8, while host RNAs carrying modified nucleosides (m^5^c, m^6^A, m^5^U, s^2^U or pseudo-uridine) induce little or no stimulation (371). Similarly, the presence of m^6^A modifications in host circular RNAs inhibits their recognition by PRR while unmodified circular RNAs, present mostly in viral genomes, are known to activate RIG-I leading to downstream stimulation of IFN gene expression (372). The latter example also emphasize the importance of nucleoside modification readers. Indeed, YTHDF2 reader allows the discrimination between host *versus* foreign circular RNAs through the binding and the sequestration of m^6^A containing host circular RNAs (372). m^6^A modification can have additional impact during innate immune responses, favoring the expression of important immune players. Thus, it has been shown in DCs that methyltransferase-like 3 (METTL3), a well know writer of m^6^A modification (373), catalyzes the m^6^A modification of membrane co-stimulatory molecules, CD40, CD80 and a TLR signaling adaptor Tirap, during DC maturation. These modifications are read by YTHDF1, which promotes their translation by associating with translation initiation factors. m^6^A modifications thus lead to an increase of DC activation and function, promoting T-cell activation (360, 371, 374, 375),. YTHDF3, another reader of m^6^A in RNAs has been shown to bind and promote translation of the transcription factor forkhead box protein O3 (FOXO3) RNA, a negative regulator of ISGs expression (355). By binding to the 5’UTR of FOXO3 transcript, YTHDF3 cooperates with PABP1 and eIF4G2 to stimulate its translation (see **Figure 7C**). Although YTHDF3 recognizes m^6^A modified RNA targets to regulate their translation, its recruitment to FOXO3 mRNAs appears independent of METTL3 mediated m^6^A addition. Nevertheless, the interaction depends on the hydrophobic pocket of YTHDF3 that is essential for m^6^A recognition thus suggesting that other methyltransferases could be involved in m^6^A addition on FOXO3 transcripts. Alternatively, YTHDF3 could interact with FOXO3 transcripts in a m^6^A-independent manner, or recognise other types of methylated bases (such as m^1^A). Thus, YTHDF3 participates indirectly to the negative control of antiviral responses by promoting translation of ISG inhibitors to limit the risk of unnecessary inflammation (355). Similarly, upon viral infections, the nuclear RNA-helicase DDX46 has been shown to recruit the m^6^A demethylase protein ALKBH5 to the MAVS, Trif3 and Trif6 mRNAs inducing their nuclear retention to avoid their translation and prevent prolonged activation of the antiviral response (376). Dynamic m^6^A modification is also involved in the antiviral response against DNA viruses. For instance, the viral DNA sensor hnRNPA2B1, in addition to its role in inducing the IFN signaling pathway, has been shown to interact with mRNAs coding for many innate immunity factors such as cGAS, STING and IFI16 (an ISG) (356, 357). In the absence of infection, hnRNPA2B1 interacts with the m^6^A demethylase FTO to remove m^6^A from hnRNPA2B1 targets and induce their nuclear retention. Upon viral DNA sensing, the interaction between FTO and hnRNPA2B1 is disrupted, leading to increase m^6^A levels in hnRNPA2B1 target transcripts, therefore allowing their efficient nuclear export and translation to improve the antiviral response (**Figure 7D**).

Taken together, these results suggest that epitranscriptomics is a strategy used to prevent autoimmunity and balance the risk between an aberrant induction of innate response and self-tolerance (353, 362). However, more investigations are required to precisely apprehend the complex interplay between self-RNA and viral-RNA modifications, since factors involved in self-RNA disguise are not necessarily detrimental for viral RNA (and *vice versa*), suggesting that each of these factors is involved in several cellular mechanisms.

## 8 Conclusions and Perspectives

Through their capacity to interact with specific RNA sequences, structural features or chemical modifications, RBPs orchestrate all steps of RNA metabolism from its synthesis, maturation and functional role, to its eventual decay. In the context of innate immunity, RBPs play multiple roles, acting at the first line of defense through their capacity to sense non-self RNAs and induce an immune response, being involved in the activation and effector functions of innate immune cells by finely tuning the amplitude and temporal control of gene expression, and finally acting as key effectors of the antiviral response through their capacity to destroy foreign RNAs. The activity of RBPs is tightly regulated by numerous mechanisms that include control of their subcellular localization, competition with other RBPs for their RNA substrate, or post-translational modifications that modulate their activity. Working in a collaborative network with signal transducers, epigenetic modifiers, and canonical trans-acting factors, RBPs play important roles in developing a global and complex gene regulatory network. Illustrating their importance in finely tuning the innate immune response, numerous human pathologies, such as autoimmune diseases, are associated to mutations in genes coding for RBPs [for a review see (377)]. Technical developments such as the RNA interactome capture (RIC) (31) and CLIP-seq have facilitated the functional identification of new RBPs and the characterization of their exact RNA binding sites. To date, more than 1000 human encoded proteins have been shown to display RNA-binding capacity (378) and the precise RNA target sites for several hundreds of these RBPs have been mapped (62). Nevertheless, we are still far from understanding precisely the numerous roles of RBPs and their mechanisms of action in the context of innate immunity. The rapid development of efficient gene editing technologies coupled with new protocols to quantitatively monitor gene expression at multiple levels (transcription and degradation rates, splicing and translation) in bulk and single-cells, as well as methods to quantitatively assess the binding dynamics of RBPs in single-molecule or transcriptome-wide assays opens the door for new and exciting discoveries in the field of RBPs and innate immunity.

## Author Contributions

AG and ER determined the scope and focus of the review with feedback from AK and MW. AG drafted the manuscript with contributions from ER and MW. AK generated most figures with help from AG and ER. All authors contributed to the article and approved the submitted version.

## Funding

Work in our laboratory is supported by the European Research Council (ERC-StG-LS6-805500) under the European Union’s Horizon 2020 research and innovation programs; ATIP-Avenir program; Fondation FINOVI; Agence Nationale des Recherches sur le SIDA et les Hépatites Virales (ANRS-ECTZ3306) and Agence Nationale de la Recherche (ANR-20-CE15-0025).

## Conflict of Interest

The authors declare that the research was conducted in the absence of any commercial or financial relationships that could be construed as a potential conflict of interest.

## Publisher’s Note

All claims expressed in this article are solely those of the authors and do not necessarily represent those of their affiliated organizations, or those of the publisher, the editors and the reviewers. Any product that may be evaluated in this article, or claim that may be made by its manufacturer, is not guaranteed or endorsed by the publisher.

## References

[1] Swamy M Jamora C Havran W Hayday A. Epithelial Decision Makers: In Search of the “Epimmunome”. Nat Immunol (2010) 11:656–65. doi: 10.1038/ni.1905 PMC2950874 20644571

[2] Kaye PM. Stromal Cell Responses in Infection. Stromal Immunol Adv Exp Med Biol (2018) 14:23–36. doi: 10.1007/978-3-319-78127-3_2 30155620

[3] Sato A Iwasaki A. Induction of Antiviral Immunity Requires Toll-Like Receptor Signaling in Both Stromal and Dendritic Cell Compartments. PNAS (2004) 101:16274–9. doi: 10.1073/pnas.0406268101 PMC528964 15534227

[4] Constant DA Nice TJ Rauch I. Innate Immune Sensing by Epithelial Barriers. Curr Opin Immunol (2021) 73:1–8. doi: 10.1016/j.coi.2021.07.014 34392232 PMC8648961

[5] Cabeza-Cabrerizo M Cardoso A Minutti CM Pereira da Costa M Reis e Sousa C. Dendritic Cells Revisited. Annu Rev Immunol (2021) 39:131–66. doi: 10.1146/annurev-immunol-061020-053707 33481643

[6] Durbin JE Fernandez-Sesma A Lee CK Rao TD Frey AB Moran TM. Type I Ifn Modulates Innate and Specific Antiviral Immunity. J Immunol (2000) 164:4220–8. doi: 10.4049/jimmunol.164.8.4220 10754318

[7] Kohlmeier JE Cookenham T Roberts AD Miller SC Woodland DL. Type I Interferons Regulate Cytolytic Activity of Memory Cd8+ T Cells in the Lung Airways During Respiratory Virus Challenge. Immunity (2010) 33:96–105. doi: 10.1016/j.immuni.2010.06.016 20637658 PMC2908370

[8] Bianchi ME. Damps, Pamps and Alarmins: All We Need to Know About Danger. J Leukocyte Biol (2007) 81:1–5. doi: 10.1189/jlb.0306164 17032697

[9] Thompson MR Kaminski JJ Kurt-Jones EA Fitzgerald KA. Pattern Recognition Receptors and the Innate Immune Response to Viral Infection. Viruses (2011) 3:920–40. doi: 10.3390/v3060920 PMC3186011 21994762

[10] Schiller M Parcina M Heyder P Foermer S Ostrop J Leo A. Induction of Type I Ifn Is a Physiological Immune Reaction to Apoptotic Cell-Derived Membrane Microparticles. J Immunol (2012) 189:1747–56. doi: 10.4049/jimmunol.1100631 22786771

[11] Gokhale NS Smith JR Van Gelder RD Savan R. RNA Regulatory Mechanisms That Control Antiviral Innate Immunity. Immunol Rev (2021) 304:imr.13019. doi: 10.1111/imr.13019 PMC8616819 34405416

[12] Takeuchi O Akira S. Pattern Recognition Receptors and Inflammation. Cell (2010) 140:805–20. doi: 10.1016/j.cell.2010.01.022 20303872

[13] Chovatiya R Medzhitov R. Stress, Inflammation, and Defense of Homeostasis. Mol Cell (2014) 54:281–8. doi: 10.1016/j.molcel.2014.03.030 PMC4048989 24766892

[14] Fan J Ishmael FT Fang X Myers A Cheadle C Huang SK. Chemokine Transcripts as Targets of the RNA-Binding Protein HuR in Human Airway Epithelium. J Immunol (2011) 186:2482–94. doi: 10.4049/jimmunol.0903634 PMC3872785 21220697

[15] Lin JX Leonard WJ. Fine-Tuning Cytokine Signals. Annu Rev Immunol (2019) 37:295–324. doi: 10.1146/annurev-immunol-042718-041447 30649989 PMC10822674

[16] Cavaillon JM. Pro- Versus Anti-Inflammatory Cytokines: Myth or Reality. Cell Mol Biol (Noisy-Le-Grand France) (2001) 47:695–702. 11502077

[17] Guo H Callaway JB Ting JPY. Inflammasomes: Mechanism of Action, Role in Disease, and Therapeutics. Nat Med (2015) 21:677–87. doi: 10.1038/nm.3893 PMC4519035 26121197

[18] Pietras EM. Inflammation: A Key Regulator of Hematopoietic Stem Cell Fate in Health and Disease. Blood (2017) 130:1693–8. doi: 10.1182/blood-2017-06-780882 PMC5639485 28874349

[19] Smale ST. Transcriptional Regulation in the Innate Immune System. Curr Opin Immunol (2012) 24:51–7. doi: 10.1016/j.coi.2011.12.008 PMC3288296 22230561

[20] Medzhitov R Horng T. Transcriptional Control of the Inflammatory Response. Nat Rev Immunol (2009) 9:692–703. doi: 10.1038/nri2634 19859064

[21] Carpenter S Ricci EP Mercier BC Moore MJ Fitzgerald KA. Post-Transcriptional Regulation of Gene Expression in Innate Immunity. Nat Rev Immunol (2014) 14:361–76. doi: 10.1038/nri3682 24854588

[22] Anderson P. Post-Transcriptional Regulons Coordinate the Initiation and Resolution of Inflammation. Nat Rev Immunol (2010) 12:24–35. doi: 10.1038/nri2685 20029446

[23] Rauscher R Ignatova Z. Tuning Innate Immunity by Translation. Biochem Soc Trans (2015) 43:1247–52. doi: 10.1042/BST20150166 26614668

[24] García-Mauriño SM Rivero-Rodríguez F Velázquez-Cruz A Hernández-Vellisca M Díaz-Quintana A de la Rosa MA. RNA Binding Protein Regulation and Cross-Talk in the Control of AU-Rich mRNA Fate. Front Mol Biosci (2017) 4:71. doi: 10.3389/fmolb.2017.00071 29109951 PMC5660096

[25] Turner M Díaz-Muñoz MD. RNA-Binding Proteins Control Gene Expression and Cell Fate in the Immune System. Nat Immunol (2018) 19:120–9. doi: 10.1038/s41590-017-0028-4 29348497

[26] Kafasla P Skliris A Kontoyiannis DL. Post-Transcriptional Coordination of Immunological Responses by RNA-Binding Proteins. Nat Immunol (2014) 15:492–502. doi: 10.1038/ni.2884 24840980

[27] Gerstberger S Hafner M Tuschl T. A Census of Human RNA-Binding Proteins. Nat Rev Genet (2014) 15:829–45. doi: 10.1038/nrg3813 PMC11148870 25365966

[28] Hudson WH Ortlund EA. The Structure, Function and Evolution of Proteins That Bind DNA and RNA. Nat Rev Mol Cell Biol (2014) 15:749–60. doi: 10.1038/nrm3884 PMC4280011 25269475

[29] Re A Joshi T Kulberkyte E Morris Q Workman CT. RNA–Protein Interactions: An Overview. In: Gorodkin J Ruzzo WL, editors. RNA Sequence, Structure, and Function: Computational and Bioinformatic Methods, vol. 1097. Totowa, NJ: Humana Press (2014). p. 491–521. doi: 10.1007/978-1-62703-709-9 24639174

[30] Hentze MW Castello A Schwarzl T Preiss T. A Brave New World of RNA-Binding Proteins. Nat Rev Mol Cell Biol (2018) 19:327–41. doi: 10.1038/nrm.2017.130 29339797

[31] Garcia-Moreno M Noerenberg M Ni S Järvelin AI González-Almela E Lenz CE. System-Wide Profiling of RNA-Binding Proteins Uncovers Key Regulators of Virus Infection. Mol Cell (2019) 74:196–211.e11. doi: 10.1016/j.molcel.2019.01.017 30799147 PMC6458987

[32] Liepelt A Naarmann-de Vries IS Simons N Eichelbaum K Föhr S Archer SK. Identification of RNA-Binding Proteins in Macrophages by Interactome Capture. Mol Cell Proteomics (2016) 15:2699–714. doi: 10.1074/mcp.M115.056564 PMC4974345 27281784

[33] Schmidt N Lareau CA Keshishian H Ganskih S Schneider C Hennig T. The Sars-Cov-2 RNA–Protein Interactome in Infected Human Cells. Nat Microbiol (2021) 6:339–53. doi: 10.1038/s41564-020-00846-z PMC7906908 33349665

[34] Kamel W Noerenberg M Cerikan B Chen H Järvelin AI Kammoun M. Global Analysis of Protein-RNA Interactions in Sars-Cov-2-Infected Cells Reveals Key Regulators of Infection. Mol Cell (2021) 81:2851–67.e7. doi: 10.1016/j.molcel.2021.05.023 PMC8142890 34118193

[35] Ramanathan M Porter DF Khavari PA. Methods to Study RNA–protein Interactions. Nat Methods (2019) 16:225–34. doi: 10.1038/s41592-019-0330-1 PMC6692137 30804549

[36] Zhang C Darnell RB. Mapping *In Vivo* Protein-RNA Interactions at Single-Nucleotide Resolution From Hits-Clip Data. Nat Biotechnol (2011) 29:607–14. doi: 10.1038/nbt.1873 PMC3400429 21633356

[37] Wheeler EC Van Nostrand EL Yeo GW. Advances and Challenges in the Detection of Transcriptome-Wide Protein– RNA Interactions. WIREs RNA (2018) 9:e1436. doi: 10.1002/wrna.1436 PMC5739989 28853213

[38] Liu ZR Wilkie AM Clemens MJ Smith CW. Detection of Double-Stranded RNA-Protein Interactions by Methylene Blue-Mediated Photo-Crosslinking. RNA (1996) 2:611–21. PMC1369400 8718690

[39] Ricci EP Kucukural A Cenik C Mercier BC Singh G Heyer EE. Staufen1 Senses Overall Transcript Secondary Structure to Regulate Translation. Nat Struct Mol Biol (2014) 21:26–35. doi: 10.1038/nsmb.2739 24336223 PMC4605437

[40] Urdaneta EC Beckmann BM. Fast and Unbiased Purification of RNA-Protein Complexes After UV Cross-Linking. Methods (2020) 178:72–82. doi: 10.1016/j.ymeth.2019.09.013 31586594

[41] Jensen KB Darnell RB. Clip: Crosslinking and Immunoprecipitation of *In Vivo* RNA Targets of RNA-Binding Proteins. Methods Mol Biol (Clifton NJ) (2008) 488:85–98. doi: 10.1007/978-1-60327-475-3_6 PMC4492529 18982285

[42] Beckmann BM. RNA Interactome Capture in Yeast. Methods (2017) 118-119:82–92. doi: 10.1016/j.ymeth.2016.12.008 27993706 PMC5421583

[43] Hafner M Landthaler M Burger L Khorshid M Hausser J Berninger P. Transcriptome-Wide Identification of RNA-Binding Protein and MicroRNA Target Sites by PAR-CLIP. Cell (2010) 141:129–41. doi: 10.1016/j.cell.2010.03.009 PMC2861495 20371350

[44] Liu ZR Sargueil B Smith CW. Methylene Blue-Mediated Cross-Linking of Proteins to Double-Stranded RNA. Methods Enzymol (2000) 318:22–33. doi: 10.1016/S0076-6879(00)18041-3 10889977

[45] Singh G Ricci EP Moore MJ. Ripit-Seq: A High-Throughput Approach for Footprinting RNA:Protein Complexes. Methods (2014) 65:320–32. doi: 10.1016/j.ymeth.2013.09.013 PMC3943816 24096052

[46] Woodward L Gangras P Singh G. Identification of Footprints of RNA:Protein Complexes *via* RNA Immunoprecipitation in Tandem Followed by Sequencing (Ripit-Seq). J Visual Experiments (2019) 59913:e59913. doi: 10.3791/59913 PMC6697088 31355789

[47] Patton RD Sanjeev M Woodward LA Mabin JW Bundschuh R Singh G. Chemical Crosslinking Enhances RNA Immunoprecipitation for Efficient Identification of Binding Sites of Proteins That Photo-Crosslink Poorly With RNA. RNA (2020) 26:1216–33. doi: 10.1261/rna.074856.120 PMC7430673 32467309

[48] Yi Z Singh G. Ripit-Seq: A Tandem Immunoprecipitation Approach to Reveal Global Binding Landscape of Multisubunit Ribonucleoproteins. Methods Enzymol (2021) 655:401–25. doi: 10.1016/bs.mie.2021.03.019 PMC8358897 34183131

[49] Singh G Kucukural A Cenik C Leszyk J Shaffer S Weng Z. The Cellular Ejc Interactome Reveals Higher-Order Mrnp Structure and an Ejc-Sr Protein Nexus. Cell (2012) 151:750–64. doi: 10.1016/j.cell.2012.10.007 PMC3522173 23084401

[50] Castello A Horos R Strein C Fischer B Eichelbaum K Steinmetz LM. System-Wide Identification of RNA-Binding Proteins by Interactome Capture. Nat Protoc (2013) 8:491–500. doi: 10.1038/nprot.2013.020 23411631

[51] Perez-Perri JI Rogell B Schwarzl T Stein F Zhou Y Rettel M. Discovery of RNA-Binding Proteins and Characterization of Their Dynamic Responses by Enhanced RNA Interactome Capture. Nat Commun (2018) 9:4408. doi: 10.1038/s41467-018-06557-8 30352994 PMC6199288

[52] McHugh CA Guttman M. RAP-MS: A Method to Identify Proteins That Interact Directly With a Specific RNA Molecule in Cells. Methods Mol Biol (2018) 1649:473–88. doi: 10.1007/978-1-4939-7213-5_31 29130217

[53] Castello A Fischer B Eichelbaum K Horos R Beckmann B Strein C. Insights Into RNA Biology From an Atlas of Mammalian mRNA-Binding Proteins. Cell (2012) 149:1393–406. doi: 10.1016/j.cell.2012.04.031 22658674

[54] Castello A Horos R Strein C Fischer B Eichelbaum K Steinmetz LM. Comprehensive Identification of RNA-Binding Proteins by RNA Interactome Capture. Methods Mol Biol (2016) 1358:131–9. doi: 10.1007/978-1-4939-3067-8_8 26463381

[55] Bantscheff M Lemeer S Savitski MM Kuster B. Quantitative Mass Spectrometry in Proteomics: Critical Review Update From 2007 to the Present. Anal Bioanal Chem (2012) 404:939–65. doi: 10.1007/s00216-012-6203-4 22772140

[56] Grandi P Bantscheff M. Advanced Proteomics Approaches to Unravel Protein Homeostasis. Drug Discov Today: Technol (2019) 31:99–108. doi: 10.1016/j.ddtec.2019.02.001 31200865

[57] de Vries S Benes V Naarmann-de Vries IS Rücklé C Zarnack K Marx G. P23 Acts as Functional Rbp in the Macrophage Inflammation Response. Front Mol Biosci (2021) 8:625608. doi: 10.3389/fmolb.2021.625608 34179071 PMC8226254

[58] Ule J. CLIP Identifies Nova-Regulated RNA Networks in the Brain. Science (2003) 302:1212–5. doi: 10.1126/science.1090095 14615540

[59] Huppertz I Attig J D’Ambrogio A Easton LE Sibley CR Sugimoto Y. Iclip: Protein–RNA Interactions at Nucleotide Resolution. Methods (2014) 65:274–87. doi: 10.1016/j.ymeth.2013.10.011 PMC3988997 24184352

[60] Van Nostrand EL Pratt GA Shishkin AA Gelboin-Burkhart C Fang MY Sundararaman B. Robust Transcriptome-Wide Discovery of RNA-Binding Protein Binding Sites With Enhanced Clip (Eclip). Nat Methods (2016) 13:508–14. doi: 10.1038/nmeth.3810 PMC4887338 27018577

[61] Heyl F Maticzka D Uhl M Backofen R. Galaxy Clip-Explorer: A Web Server for Clip-Seq Data Analysis. GigaScience (2020) 9:giaa108. doi: 10.1093/gigascience/giaa108 33179042 PMC7657819

[62] Van Nostrand EL Freese P Pratt GA Wang X Wei X Xiao R. A Large-Scale Binding and Functional Map of Human RNA-Binding Proteins. Nature (2020) 583:711–9. doi: 10.1038/s41586-020-2077-3 PMC7410833 32728246

[63] Sharma D Zagore LL Brister MM Ye X Crespo-Hernández CE Licatalosi DD. The Kinetic Landscape of an RNA-Binding Protein in Cells. Nature (2021) 591:152–6. doi: 10.1038/s41586-021-03222-x PMC8299502 33568810

[64] Uehata T Takeuchi O. RNA Recognition and Immunity—Innate Immune Sensing and Its Posttranscriptional Regulation Mechanisms. Cells (2020) 9:1701. doi: 10.3390/cells9071701 PMC7407594 32708595

[65] Hardy MP Audemard Ã Migneault F Feghaly A Brochu S Gendron P. Apoptotic Endothelial Cells Release Small Extracellular Vesicles Loaded With Immunostimulatory Viral-Like RNAs. Sci Rep (2019) 9:7203. doi: 10.1038/s41598-019-43591-y 31076589 PMC6510763

[66] Nabet BY Qiu Y Shabason JE Wu TJ Yoon T Kim BC. Exosome RNA Unshielding Couples Stromal Activation to Pattern Recognition Receptor Signaling in Cancer. Cell (2017) 170:352–66.e13. doi: 10.1016/j.cell.2017.06.031 PMC6611169 28709002

[67] Alexopoulou L Czopik Holt A Medzhitov R Flavell RA. Recognition of double-strandedRNA and Activation of NF-Kbby Toll-Like Receptor 3. Nature (2001) 413:732–8. doi: 10.1038/35099560 11607032

[68] Leonard JN Ghirlando R Askins J Bell JK Margulies DH Davies DR. The TLR3 Signaling Complex Forms by Cooperative Receptor Dimerization. Proc Natl Acad Sci (2008) 105:258–63. doi: 10.1073/pnas.0710779105 PMC2224197 18172197

[69] Tatematsu M Nishikawa F Seya T Matsumoto M. Toll-Like Receptor 3 Recognizes Incomplete Stem Structures in Single-Stranded Viral RNA. Nat Commun (2013) 4:1833. doi: 10.1038/ncomms2857 23673618

[70] Heil F. Species-Specific Recognition of Single-Stranded RNA *via* Toll-Like Receptor 7 and 8. Science (2004) 303:1526–9. doi: 10.1126/science.1093620 14976262

[71] Greulich W Wagner M Gaidt MM Stafford C Cheng Y Linder A. TLR8 Is a Sensor of RNAse T2 Degradation Products. Cell (2019) 179:1264–75.e13. doi: 10.1016/j.cell.2019.11.001 PMC7116005 31778653

[72] Diebold SS. Innate Antiviral Responses by Means of TLR7-Mediated Recognition of Single-Stranded RNA. Science (2004) 303:1529–31. doi: 10.1126/science.1093616 14976261

[73] Tanji H Ohto U Shibata T Taoka M Yamauchi Y Isobe T. Toll-Like Receptor 8 Senses Degradation Products of Single-Stranded RNA. Nat Struct Mol Biol (2015) 22:109–15. doi: 10.1038/nsmb.2943 25599397

[74] Cavassani KA Ishii M Wen H Schaller MA Lincoln PM Lukacs NW. TLR3 Is an Endogenous Sensor of Tissue Necrosis During Acute Inflammatory Events. J Exp Med (2008) 205:2609–21. doi: 10.1084/jem.20081370 PMC2571935 18838547

[75] Rehwinkel J Gack MU. RIG-I-Like Receptors: Their Regulation and Roles in RNA Sensing. Nat Rev Immunol (2020) 20:537–51. doi: 10.1038/s41577-020-0288-3 PMC7094958 32203325

[76] He WT Wan H Hu L Chen P Wang X Huang Z. Gasdermin D Is an Executor of Pyroptosis and Required for Interleukin-1β Secretion. Cell Res (2015) 25:1285–98. doi: 10.1038/cr.2015.139 PMC4670995 26611636

[77] Stogerer T Stager S. Innate Immune Sensing by Cells of the Adaptive Immune System. Front Immunol (2020) 11:1081. doi: 10.3389/fimmu.2020.01081 32547564 PMC7274159

[78] Wang Y Yuan S Jia X Ge Y Ling T Nie M. Mitochondria-Localised ZNFX1 Functions as a dsRNA Sensor to Initiate Antiviral Responses Through MAVS. Nat Cell Biol (2019) 21:1346–56. doi: 10.1038/s41556-019-0416-0 31685995

[79] Mahony R Broadbent L Maier-Moore JS Power UF Jefferies CA. The RNA Binding Protein La/SS-B Promotes RIG-I-Mediated Type I and Type III IFN Responses Following Sendai Viral Infection. Sci Rep (2017) 7:14537. doi: 10.1038/s41598-017-15197-9 29109527 PMC5673980

[80] Wickenhagen A Sugrue E Lytras S Kuchi S Noerenberg M Turnbull ML. A Prenylated Dsrna Sensor Protects Against Severe Covid-19. Science (2021) 374:eabj3624. doi: 10.1126/science.abj3624 34581622 PMC7612834

[81] Hung T Pratt GA Sundararaman B Townsend MJ Chaivorapol C Bhangale T. The Ro60 Autoantigen Binds Endogenous Retroelements and Regulates Inflammatory Gene Expression. Science (2015) 350:455–9. doi: 10.1126/science.aac7442 PMC4691329 26382853

[82] Lee S Karki R Wang Y Nguyen LN Kalathur RC Kanneganti TD. Aim2 Forms a Complex With Pyrin and Zbp1 to Drive Panoptosis and Host Defence. Nature (2021) 597:415–9. doi: 10.1038/s41586-021-03875-8 PMC8603942 34471287

[83] Jiao H Wachsmuth L Kumari S Schwarzer R Lin J Eren RO. Z-Nucleic-Acid Sensing Triggers ZBP1-Dependent Necroptosis and Inflammation. Nature (2020) 580:391–5. doi: 10.1038/s41586-020-2129-8 PMC7279955 32296175

[84] Zhang T Yin C Boyd DF Quarato G Ingram JP Shubina M. Influenza Virus Z-Rnas Induce Zbp1-Mediated Necroptosis. Cell (2020) 180:1115–29.e13. doi: 10.1016/j.cell.2020.02.050 PMC7153753 32200799

[85] Thomas PG Shubina M Balachandran S. ZBP1/DAI-Dependent Cell Death Pathways in Influenza A Virus Immunity and Pathogenesis (Springer Berlin Heidelberg). Curr Topics Microbiol Immunol (2019) 1–23. doi: 10.1007/82_2019_90 31970498

[86] Zhang Z Ohto U Shibata T Krayukhina E Taoka M Yamauchi Y. Structural Analysis Reveals That Toll-Like Receptor 7 Is a Dual Receptor for Guanosine and Single-Stranded RNA. Immunity (2016) 45:737–48. doi: 10.1016/j.immuni.2016.09.011 27742543

[87] Ostendorf T Zillinger T Andryka K Schlee-Guimaraes TM Schmitz S Marx S. Immune Sensing of Synthetic, Bacterial, and Protozoan RNA by Toll-Like Receptor 8 Requires Coordinated Processing by RNase T2 and RNase 2. Immunity (2020) 52:591–605.e6. doi: 10.1016/j.immuni.2020.03.009 32294405

[88] Liu K Sato R Shibata T Hiranuma R Reuter T Fukui R. Skewed Endosomal RNA Responses From Tlr7 to Tlr3 in RNAse T2-Deficient Macrophages. Int Immunol (2021) 33:479–90. doi: 10.1093/intimm/dxab033 34161582

[89] Zhang Z Ohto U Shibata T Taoka M Yamauchi Y Sato R. Structural Analyses of Toll-Like Receptor 7 Reveal Detailed RNA Sequence Specificity and Recognition Mechanism of Agonistic Ligands. Cell Rep (2018) 25:3371–81.e5. doi: 10.1016/j.celrep.2018.11.081 30566863

[90] Shibata T Taoka M Saitoh SI Yamauchi Y Motoi Y Komine M. Nucleosides Drive Histiocytosis in SLC29A3 Disorders by Activating TLR7. Immunology (2019). doi: 10.1101/2019.12.16.877357

[91] Haroche J Cohen-Aubart F Rollins BJ Donadieu J Charlotte F Idbaih A. Histiocytoses: Emerging Neoplasia Behind Inflammation. Lancet Oncol (2017) 18:e113–25. doi: 10.1016/S1470-2045(17)30031-1 28214412

[92] Pichlmair A Schulz O Tan CP Naslund TI Liljestrom P Weber F. RIG-I-Mediated Antiviral Responses to Single-Stranded RNA Bearing 5’-Phosphates. Science (2006) 314:997–1001. doi: 10.1126/science.1132998 17038589

[93] Goubau D Schlee M Deddouche S Pruijssers AJ Zillinger T Goldeck M. Antiviral Immunity *via* RIG-I-Mediated Recognition of RNA Bearing 5’-Diphosphates. Nature (2014) 514:372–5. doi: 10.1038/nature13590 PMC4201573 25119032

[94] Hornung V Ellegast J Kim S Brzózka K Jung A Kato H. 5’-Triphosphate RNA Is the Ligand for RIG-I. Science (2006) 314:994–7. doi: 10.1126/science.1132505 17038590

[95] Schuberth-Wagner C Ludwig J Bruder AK Herzner AM Zillinger T Goldeck M. A Conserved Histidine in the RNA Sensor RIG-I Controls Immune Tolerance to N1-2’o-Methylated Self Rna. Immunity (2015) 43:41–51. doi: 10.1016/j.immuni.2015.06.015 26187414 PMC7128463

[96] Kato H Takeuchi O Sato S Yoneyama M Yamamoto M Matsui K. Differential Roles of MDA5 and RIG-I Helicases in the Recognition of RNA Viruses. Nature (2006) 441:101–5. doi: 10.1038/nature04734 16625202

[97] Pichlmair A Schulz O Tan CP Rehwinkel J Kato H Takeuchi O. Activation of MDA5 Requires Higher-Order RNA Structures Generated During Virus Infection. JVI (2009) 83:10761–9. doi: 10.1128/JVI.00770-09 PMC2753146 19656871

[98] Kato H Takeuchi O Mikamo-Satoh E Hirai R Kawai T Matsushita K. Length-Dependent Recognition of Double-Stranded Ribonucleic Acids by Retinoic Acid–Inducible Gene-I and Melanoma Differentiation–Associated Gene 5. J Exp Med (2008) 205:1601–10. doi: 10.1084/jem.20080091 PMC2442638 18591409

[99] Runge S Sparrer KMJ Lässig C Hembach K Baum A García-Sastre A. *In Vivo* Ligands of MDA5 and RIG-I in Measles Virus-Infected Cells. PloS Pathog (2014) 10:e1004081. doi: 10.1371/journal.ppat.1004081 24743923 PMC3990713

[100] Berke IC Modis Y. MDA5 Cooperatively Forms Dimers and ATP-Sensitive Filaments Upon Binding Double-Stranded RNA: MDA5 Forms Dimers and Filaments on Binding dsRNA. EMBO J (2012) 31:1714–26. doi: 10.1038/emboj.2012.19 PMC3321199 22314235

[101] Peisley A Lin C Wu B Orme-Johnson M Liu M Walz T. Cooperative Assembly and Dynamic Disassembly of MDA5 Filaments for Viral dsRNA Recognition. Proc Natl Acad Sci (2011) 108:21010–5. doi: 10.1073/pnas.1113651108 PMC3248507 22160685

[102] Venkataraman T Valdes M Elsby R Kakuta S Caceres G Saijo S. Loss of DExD/H Box RNA Helicase LGP2 Manifests Disparate Antiviral Responses. J Immunol (2007) 178:6444–55. doi: 10.4049/jimmunol.178.10.6444 17475874

[103] Broz P Dixit VM. Inflammasomes: Mechanism of Assembly, Regulation and Signalling. Nat Rev Immunol (2016) 16:407–20. doi: 10.1038/nri.2016.58 27291964

[104] Bauernfried S Scherr MJ Pichlmair A Duderstadt KE Hornung V. Human NLRP1 Is a Sensor for Double-Stranded RNA. Science (2021) 371:eabd0811. doi: 10.1126/science.abd0811 33243852

[105] Vavassori S Chou J Faletti LE Haunerdinger V Opitz L Joset P. Multisystem Inflammation and Susceptibility to Viral Infections in Human ZNFX1 Deficiency. J Allergy Clin Immunol (2021) 148:381–93. doi: 10.1016/j.jaci.2021.03.045. S0091674921006138. PMC8569286 33872655

[106] Miao Z Tidu A Eriani G Martin F. Secondary Structure of the Sars-Cov-2 5’-Utr. RNA Biol (2021) 18:447–56. doi: 10.1080/15476286.2020.1814556 PMC7544965 32965173

[107] Skrivergaard S Jensen MS Rolander TB Nguyen TBN Bundgaard A Nejsum LN. The Cellular Localization of the P42 and P46 Oligoadenylate Synthetase 1 Isoforms and Their Impact on Mitochondrial Respiration. Viruses (2019) 11:1122. doi: 10.3390/v11121122 PMC6950736 31817188

[108] Bisbal C Silverman RH. Diverse Functions of Rnase L and Implications in Pathology. Biochimie (2007) 89:789–98. doi: 10.1016/j.biochi.2007.02.006 PMC2706398 17400356

[109] Cao L Liu S Li Y Yang G Luo Y Li S. The Nuclear Matrix Protein Safa Surveils Viral RNA and Facilitates Immunity by Activating Antiviral Enhancers and Super-Enhancers. Cell Host Microbe (2019) 26:369–84.e8. doi: 10.1016/j.chom.2019.08.010 31513772

[110] Liu G Gack MU. Distinct and Orchestrated Functions of RNA Sensors in Innate Immunity. Immunity (2020) 53:26–42. doi: 10.1016/j.immuni.2020.03.017 32668226 PMC7367493

[111] Zhang Z Kim T Bao M Facchinetti V Jung SY Ghaffari AA. Ddx1, Ddx21, and Dhx36 Helicases Form a Complex With the Adaptor Molecule Trif to Sense DsRNA in Dendritic Cells. Immunity (2011) 34:866–78. doi: 10.1016/j.immuni.2011.03.027 PMC3652560 21703541

[112] Zhang Z Yuan B Lu N Facchinetti V Liu YJ. Dhx9 Pairs With Ips-1 to Sense Double-Stranded RNA in Myeloid Dendritic Cells. J Immunol (Baltimore Md: 1950) (2011) 187:4501–8. doi: 10.4049/jimmunol.1101307 PMC3656476 21957149

[113] Mitoma H Hanabuchi S Kim T Bao M Zhang Z Sugimoto N. The Dhx33 RNA Helicase Senses Cytosolic RNA and Activates the Nlrp3 Inflammasome. Immunity (2013) 39:123–35. doi: 10.1016/j.immuni.2013.07.001 PMC3756931 23871209

[114] Zhu S Ding S Wang P Wei Z Pan W Palm NW. Nlrp9b Inflammasome Restricts Rotavirus Infection in Intestinal Epithelial Cells. Nature (2017) 546:667–70. doi: 10.1038/nature22967 PMC5787375 28636595

[115] Oshiumi H Sakai K Matsumoto M Seya T. Dead/h Box 3 (Ddx3) Helicase Binds the Rig-I Adaptor Ips-1 to Up-Regulate Ifn-Beta-Inducing Potential. Eur J Immunol (2010) 40:940–8. doi: 10.1002/eji.200940203 20127681

[116] Miyashita M Oshiumi H Matsumoto M Seya T. Ddx60, a Dexd/H Box Helicase, Is a Novel Antiviral Factor Promoting Rig-I-Like Receptor-Mediated Signaling. Mol Cell Biol (2011) 31:3802–19. doi: 10.1128/MCB.01368-10 PMC3165724 21791617

[117] Kumar H Kawai T Kato H Sato S Takahashi K Coban C. Essential Role of IPS-1 in Innate Immune Responses Against RNA Viruses. J Exp Med (2006) 203:1795–803. doi: 10.1084/jem.20060792 PMC2118350 16785313

[118] Poeck H Bscheider M Gross O Finger K Roth S Rebsamen M. Recognition of RNA Virus by RIG-I Results in Activation of CARD9 and Inflammasome Signaling for Interleukin 1β Production. Nat Immunol (2010) 11:63–9. doi: 10.1038/ni.1824 19915568

[119] Rudin CM Thompson CB. Transcriptional Activation of Short Interspersed Elements by Dna-Damaging Agents. Genes Chromosomes Cancer (2001) 30:64–71. doi: 10.1002/1098-2264(2000)9999:9999<::AID-GCC1066>3.0.CO;2-F 11107177

[120] Liu WM Chu WM Choudary PV Schmid CW. Cell Stress and Translational Inhibitors Transiently Increase the Abundance of Mammalian Sine Transcripts. Nucleic Acids Res (1995) 23:1758–65. doi: 10.1093/nar/23.10.1758 PMC306933 7784180

[121] Russanova VR Driscoll CT Howard BH. Adenovirus Type 2 Preferentially Stimulates Polymerase Iii Transcription of Alu Elements by Relieving Repression: A Potential Role for Chromatin. Mol Cell Biol (1995) 15:4282–90. doi: 10.1128/MCB.15.8.4282 PMC230667 7623822

[122] Panning B Smiley JR. Activation of RNA Polymerase Iii Transcription of Human Alu Repetitive Elements by Adenovirus Type 5: Requirement for the Elb 58-Kilodalton Protein and the Products of E4 Open Reading Frames 3 and 6. Mol Cell Biol (1993) 13:14. doi: 10.1128/mcb.13.6.3231-3244.1993 PMC359768 7684492

[123] Lander ES Linton LM Birren B Nusbaum C Zody MC Baldwin J. Initial Sequencing and Analysis of the Human Genome. Nature (2001) 409:860–921. doi: 10.1038/35057062 11237011

[124] Venter JC Adams MD Myers EW Li PW Mural RJ Sutton GG. The Sequence of the Human Genome. THE Hum Genome (2001) 291:50. doi: 10.1126/science.1058040

[125] Deininger P. Alu Elements: Know the Sines. Genome Biol (2011) 12:236. doi: 10.1186/gb-2011-12-12-236 22204421 PMC3334610

[126] Panning B Smiley JR. Regulation of Cellular Genes Transduced by Herpes Simplex Virus. J Virol (1989) 63:1929–37. doi: 10.1128/jvi.63.5.1929-1937.1989 PMC250605 2539495

[127] Chattopadhyay P Srinivasa Vasudevan J Pandey R. Noncoding RNAs: Modulators and Modulatable Players During Infection-Induced Stress Response. Briefings Funct Genomics (2021) 20:28–41. doi: 10.1093/bfgp/elaa026 PMC7929421 33491070

[128] Clancy RM Alvarez D Komissarova E Barrat FJ Swartz J Buyon JP. Ro60-Associated Single-Stranded RNA Links Inflammation With Fetal Cardiac Fibrosis *via* Ligation of Tlrs: A Novel Pathway to Autoimmune-Associated Heart Block. J Immunol (2010) 184:2148–55. doi: 10.4049/jimmunol.0902248 PMC3551297 20089705

[129] Yulug IG Yulug A Fisher EM. The Frequency and Position of Alu Repeats in Cdnas, as Determined by Database Searching. Genomics (1995) 27:544–8. doi: 10.1006/geno.1995.1090 7558040

[130] Herbert A. Z-Dna and Z-RNA in Human Disease. Commun Biol (2019) 2:10. doi: 10.1038/s42003-018-0237-x 30729177 PMC6323056

[131] Nichols PJ Bevers S Henen M Kieft JS Vicens Q Vögeli B. Recognition of Non-Cpg Repeats in Alu and Ribosomal Rnas by the Z-RNA Binding Domain of Adar1 Induces a-Z Junctions. Nat Commun (2021) 12:793. doi: 10.1038/s41467-021-21039-0 33542240 PMC7862695

[132] Chung H Calis JJ Wu X Sun T Yu Y Sarbanes SL. Human Adar1 Prevents Endogenous RNA From Triggering Translational Shutdown. Cell (2018) 172:811–24.e14. doi: 10.1016/j.cell.2017.12.038 PMC5831367 29395325

[133] Ahmad S Mu X Yang F Greenwald E Park JW Jacob E. Breaching Self-Tolerance to Alu Duplex RNA Underlies Mda5-Mediated Inflammation. Cell (2018) 172:797–810.e13. doi: 10.1016/j.cell.2017.12.016 29395326 PMC5807104

[134] Karki R Sundaram B Sharma BR Lee S Malireddi RS Nguyen LN. Adar1 Restricts Zbp1-Mediated Immune Response and Panoptosis to Promote Tumorigenesis. Cell Rep (2021) 37:109858. doi: 10.1016/j.celrep.2021.109858 34686350 PMC8853634

[135] Chiang JJ Sparrer KMJ van Gent M Lässig C Huang T Osterrieder N. Viral Unmasking of Cellular 5s Rrna Pseudogene Transcripts Induces Rig-I-Mediated Immunity. Nat Immunol (2018) 19:53–62. doi: 10.1038/s41590-017-0005-y 29180807 PMC5815369

[136] Rigby RE Webb LM Mackenzie KJ Li Y Leitch A Reijns MAM. RNA:DNA Hybrids Are a Novel Molecular Pattern Sensed by TLR9. EMBO J (2014) 33:542–58. doi: 10.1002/embj.201386117 PMC3989650 24514026

[137] Sun L Wu J Du F Chen X Chen ZJ. Cyclic GMP-AMP Synthase Is a Cytosolic DNA Sensor That Activates the Type I Interferon Pathway. Science (2013) 339:786–91. doi: 10.1126/science.1232458 PMC3863629 23258413

[138] Mankan AK Schmidt T Chauhan D Goldeck M Höning K Gaidt M. Cytosolic RNA:DNA Hybrids Activate the Cgas–Sting Axis. EMBO J (2014) 33:2937–46. doi: 10.15252/embj.201488726 PMC4282641 25425575

[139] Lee YN Nechushtan H Figov N Razin E. The Function of Lysyl-tRNA Synthetase and Ap4A as Signaling Regulators of MITF Activity in Fc-RI-Activated Mast Cells. Immunity (2018) 20:145–51. doi: 10.1016/S1074-7613(04)00020-2 14975237

[140] Park SG Kim HJ Min YH Choi EC Shin YK Park BJ. Human Lysyl-Trna Synthetase Is Secreted to Trigger Proinflammatory Response. PNAS (2005) 102:6356–61. doi: 10.1073/pnas.0500226102 PMC1088368 15851690

[141] Yannay-Cohen N Carmi-Levy I Kay G Yang CM Han JM Kemeny DM. LysRS Serves as a Key Signaling Molecule in the Immune Response by Regulating Gene Expression. Mol Cell (2009) 34:603–11. doi: 10.1016/j.molcel.2009.05.019 19524539

[142] Guerra J Valadao AL Vlachakis D Polak K Vila IK Taffoni C. Lysyl-tRNA Synthetase Produces Diadenosine Tetraphosphate to Curb STING-Dependent Inflammation. Sci Adv (2020) 6:eaax3333. doi: 10.1126/sciadv.aax3333 32494729 PMC7244319

[143] Wang ET Sandberg R Luo S Khrebtukova I Zhang L Mayr C. Alternative Isoform Regulation in Human Tissue Transcriptomes. Nature (2008) 456:470–6. doi: 10.1038/nature07509 PMC2593745 18978772

[144] Kim MS Pinto SM Getnet D Nirujogi RS Manda SS Chaerkady R. A Draft Map of the Human Proteome. Nature (2014) 509:575–81. doi: 10.1038/nature13302 PMC4403737 24870542

[145] Mele M Ferreira PG Reverter F DeLuca DS Monlong J Sammeth M. The Human Transcriptome Across Tissues and Individuals. Science (2015) 348:660–5. doi: 10.1126/science.aaa0355 PMC4547472 25954002

[146] Ule J Blencowe BJ. Alternative Splicing Regulatory Networks: Functions, Mechanisms, and Evolution. Mol Cell (2019) 76:329–45. doi: 10.1016/j.molcel.2019.09.017 31626751

[147] Wang Y Liu J Huang B Xu YM Li J Huang LF. Mechanism of Alternative Splicing and Its Regulation. Biomed Rep (2015) 3:152–8. doi: 10.3892/br.2014.407 PMC4360811 25798239

[148] Rogers TF Palmer DH Wright AE. Sex-Specific Selection Drives the Evolution of Alternative Splicing in Birds. Mol Biol Evol (2020) 12:519–30. doi: 10.1093/molbev/msaa242 PMC7826194 32977339

[149] Baralle FE Giudice J. Alternative Splicing as a Regulator of Development and Tissue Identity. Nat Rev Mol Cell Biol (2017) 18:437–51. doi: 10.1038/nrm.2017.27 PMC6839889 28488700

[150] Yabas M Elliott H Hoyne G. The Role of Alternative Splicing in the Control of Immune Homeostasis and Cellular Differentiation. IJMS (2015) 17:3. doi: 10.3390/ijms17010003 PMC4730250 26703587

[151] Ergun A Doran G Costello JC Paik HH Collins JJ Mathis D. Differential Splicing Across Immune System Lineages. Proc Natl Acad Sci (2013) 110:14324–9. doi: 10.1073/pnas.1311839110 PMC3761616 23934048

[152] Martinez NM Lynch KW. Control of Alternative Splicing in Immune Responses: Many Regulators, Many Predictions, Much Still to Learn. Immunol Rev (2013) 253:216–36. doi: 10.1111/imr.12047 PMC3621013 23550649

[153] Robinson EK Jagannatha P Covarrubias S Cattle M Smaliy V Safavi R. Inflammation Drives Alternative First Exon Usage to Regulate Immune Genes Including a Novel Iron-Regulated Isoform of Aim2. eLife (2021) 10:e69431. doi: 10.7554/eLife.69431 34047695 PMC8260223

[154] Lee FFY Davidson K Harris C McClendon J Janssen WJ Alper S. Nf-κb Mediates Lipopolysaccharide-Induced Alternative Pre-Mrna Splicing of Myd88 in Mouse Macrophages. J Biol Chem (2020) 295:6236–48. doi: 10.1074/jbc.RA119.011495 PMC7196638 32179652

[155] Pai AA Baharian G Pagé Sabourin A Brinkworth JF Nédélec Y Foley JW. Widespread Shortening of 3’ Untranslated Regions and Increased Exon Inclusion Are Evolutionarily Conserved Features of Innate Immune Responses to Infection. PloS Genet (2016) 12:e1006338. doi: 10.1371/journal.pgen.1006338 27690314 PMC5045211

[156] Janssens S Burns K Vercammen E Tschopp J Beyaert R. MyD88_s_, a Splice Variant of MyD88, Differentially Modulates NF-κb- and AP-1-Dependent Gene Expression. FEBS Lett (2003) 548:103–7. doi: 10.1016/S0014-5793(03)00747-6 12885415

[157] Rao N Nguyen S Ngo K Fung-Leung WP. A Novel Splice Variant of Interleukin-1 Receptor (IL-1r)-Associated Kinase 1 Plays a Negative Regulatory Role in Toll/IL-1r-Induced Inflammatory Signaling. MCB (2005) 25:6521–32. doi: 10.1128/MCB.25.15.6521-6532.2005 PMC1190355 16024789

[158] Gray P Michelsen KS Sirois CM Lowe E Shimada K Crother TR. Identification of a Novel Human MD-2 Splice Variant That Negatively Regulates Lipopolysaccharide-Induced TLR4 Signaling. J Immunol (2010) 184:6359–66. doi: 10.4049/jimmunol.0903543 PMC3057206 20435923

[159] Tumurkhuu G Dagvadorj J Jones HD Chen S Shimada K Crother TR. Alternatively Spliced Myeloid Differentiation Protein-2 Inhibits Tlr4-Mediated Lung Inflammation. J Immunol (2015) 194:1686–94. doi: 10.4049/jimmunol.1402123 PMC4323992 25576596

[160] Seo JW Yang EJ Kim SH Choi IH. An Inhibitory Alternative Splice Isoform of Toll-Like Receptor 3 Is Induced by Type I Interferons in Human Astrocyte Cell Lines. BMB Rep (2015) 48:696–701. doi: 10.5483/BMBRep.2015.48.12.106 26077030 PMC4791326

[161] Crocker PR Paulson JC Varki A. Siglecs and Their Roles in the Immune System. Nat Rev Immunol (2007) 7:255–66. doi: 10.1038/nri2056 17380156

[162] Ali SR Fong JJ Carlin AF Busch TD Linden R Angata T. Siglec-5 and Siglec-14 Are Polymorphic Paired Receptors That Modulate Neutrophil and Amnion Signaling Responses to Group B Streptococcus. J Exp Med (2014) 211:1231–42. doi: 10.1084/jem.20131853 PMC4042635 24799499

[163] Chen GY Brown NK Wu W Khedri Z Yu H Chen X. Broad and Direct Interaction Between Tlr and Siglec Families of Pattern Recognition Receptors and Its Regulation by Neu1. eLife (2014) 3:e04066. doi: 10.7554/eLife.04066 25187624 PMC4168287

[164] Huang PCJ Low PY Wang I Hsu STD Angata T. Soluble Siglec-14 Glycan-Recognition Protein Is Generated by Alternative Splicing and Suppresses Myeloid Inflammatory Responses. J Biol Chem (2018) 293:19645–58. doi: 10.1074/jbc.RA118.005676 PMC6314137 30377253

[165] Takeda K Akira S. TLR Signaling Pathways. Semin Immunol (2004) 16:3–9. doi: 10.1016/j.smim.2003.10.003 14751757

[166] O’Neill LAJ Bowie AG. The Family of Five: Tir-Domain-Containing Adaptors in Toll-Like Receptor Signalling. Nat Rev Immunol (2007) 7:353–64. doi: 10.1038/nri2079 17457343

[167] Janssens S Burns K Tschopp J Beyaert R. Regulation of Interleukin-1- and Lipopolysaccharide-Induced Nf-*κ* B Activation by Alternative Splicing of Myd88. Curr Biol (2002) 12:467–71. doi: 10.1016/S0960-9822(02)00712-1 11909531

[168] Burns K Janssens S Brissoni B Olivos N Beyaert R Tschopp J. Inhibition of Interleukin 1 Receptor/Toll-Like Receptor Signaling Through the Alternatively Spliced, Short Form of Myd88 Is Due to Its Failure to Recruit Irak-4. J Exp Med (2003) 197:263–8. doi: 10.1084/jem.20021790 PMC2193806 12538665

[169] De Arras L Alper S. Limiting of the Innate Immune Response by SF3A-Dependent Control of MyD88 Alternative mRNA Splicing. PloS Genet (2013) 9:e1003855. doi: 10.1371/journal.pgen.1003855 24204290 PMC3812059

[170] O’Connor BP Danhorn T De Arras L Flatley BR Marcus RA Farias-Hesson E. Regulation of Toll-Like Receptor Signaling by the SF3a mRNA Splicing Complex. PloS Genet (2015) 11:e1004932. doi: 10.1371/journal.pgen.1004932 25658809 PMC4450051

[171] West KO Scott HM Torres-Odio S West AP Patrick KL Watson RO. The Splicing Factor hnRNP M Is a Critical Regulator of Innate Immune Gene Expression in Macrophages. Cell Rep (2019) 29:1594–1609.e5. doi: 10.1016/j.celrep.2019.09.078 31693898 PMC6981299

[172] Haque N Ouda R Chen C Ozato K Hogg JR. ZFR Coordinates Crosstalk Between RNA Decay and Transcription in Innate Immunity. Nat Commun (2018) 9:1145. doi: 10.1038/s41467-018-03326-5 29559679 PMC5861047

[173] De Arras L Laws R Leach SM Pontis K Freedman JH Schwartz DA. Comparative Genomics RNAi Screen Identifies Eftud2 as a Novel Regulator of Innate Immunity. Genetics (2014) 197:485–96. doi: 10.1534/genetics.113.160499 PMC4063909 24361939

[174] Iwasaki A. A Virological View of Innate Immune Recognition. Annu Rev Microbiol (2012) 66:177–96. doi: 10.1146/annurev-micro-092611-150203 PMC3549330 22994491

[175] Yang E Li MMH. All About the RNA: Interferon-Stimulated Genes That Interfere With Viral RNA Processes. Front Immunol (2020) 11:605024. doi: 10.3389/fimmu.2020.605024 33362792 PMC7756014

[176] Crosse KM Monson EA Beard MR Helbig KJ. Interferon-Stimulated Genes as Enhancers of Antiviral Innate Immune Signaling. J Innate Immun (2018) 10:85–93. doi: 10.1159/000484258 29186718 PMC5969054

[177] Wagner AR Scott HM West KO Vail KJ Fitzsimons TC Coleman AK. Global Transcriptomics Uncovers Distinct Contributions From Splicing Regulatory Proteins to the Macrophage Innate Immune Response. Front Immunol (2021) 12:656885. doi: 10.3389/fimmu.2021.656885 34305890 PMC8299563

[178] Beyer AL Christensen ME Walker BW LeStourgeon WM. Identification and Characterization of the Packaging Proteins of Core 40S hnRNP Particles. Cell (1977) 11:127–38. doi: 10.1016/0092-8674(77)90323-3 872217

[179] Eekelen CA Riemen T Venrooij WJ. Specificity in the Interaction of Hnrna and Mrna With Proteins as Revealed by *In Vivo* Cross Linking. FEBS Lett (1981) 130:223–6. doi: 10.1016/0014-5793(81)81125-8 6116619

[180] Ford LP Suh JM Wright WE Shay JW. Heterogeneous Nuclear Ribonucleoproteins C1 and C2 Associate With the RNA Component of Human Telomerase. Mol Cell Biol (2000) 20:9084–91. doi: 10.1128/MCB.20.23.9084-9091.2000 PMC86561 11074006

[181] Moumen A Masterson P O’Connor MJ Jackson SP. hnRNP K: An HDM2 Target and Transcriptional Coactivator of P53 in Response to DNA Damage. Cell (2005) 123:1065–78. doi: 10.1016/j.cell.2005.09.032 16360036

[182] Gautrey H Jackson C Dittrich AL Browell D Lennard T Tyson-Capper A. SRSF3 and hnRNP H1 Regulate a Splicing Hotspot of *HER2* in Breast Cancer Cells. RNA Biol (2015) 12:1139–51. doi: 10.1080/15476286.2015.1076610 PMC4829299 26367347

[183] Loh TJ Moon H Cho S Jang H Liu YC Tai H. CD44 Alternative Splicing and hnRNP A1 Expression Are Associated With the Metastasis of Breast Cancer. Oncol Rep (2015) 34:1231–8. doi: 10.3892/or.2015.4110 26151392

[184] Meagher MJ Schumacher JM Lee K Holdcraft RW Edelhoff S Disteche C. Identification of ZFR, an Ancient and Highly Conserved Murine Chromosome-Associated Zinc Finger Protein. Gene (1999) 228:197–211. doi: 10.1016/S0378-1119(98)00615-5 10072773

[185] Stanek D Fox AH. Nuclear Bodies: News Insights Into Structure and Function. Curr Opin Cell Biol (2017) 46:94–101. doi: 10.1016/j.ceb.2017.05.001 28577509

[186] Machyna M Kehr S Straube K Kappei D Buchholz F Butter F. The Coilin Interactome Identifies Hundreds of Small Noncoding Rnas That Traffic Through Cajal Bodies. Mol Cell (2014) 56:389–99. doi: 10.1016/j.molcel.2014.10.004 25514182

[187] Xu H Pillai RS Azzouz TN Shpargel KB Kambach C Hebert MD. The C-Terminal Domain of Coilin Interacts With Sm Proteins and U Snrnps. Chromosoma (2005) 114:155–66. doi: 10.1007/s00412-005-0003-y PMC1389727 16003501

[188] Lemm I Girard C Kuhn AN Watkins NJ Schneider M Bordonné R. Ongoing U Snrnp Biogenesis Is Required for the Integrity of Cajal Bodies. Mol Biol Cell (2006) 17:3221–31. doi: 10.1091/mbc.e06-03-0247 PMC1483051 16687569

[189] Lee S Lee TA Lee E Kang S Park A Kim SW. Identification of a Subnuclear Body Involved in Sequence-Specific Cytokine RNA Processing. Nat Commun (2015) 6:5791. doi: 10.1038/ncomms6791 25557830

[190] Lee S Park B. InSAC: A Novel Sub-Nuclear Body Essential for Interleukin-6 and -10 RNA Processing and Stability. BMB Rep (2015) 48:239–40. doi: 10.5483/BMBRep.2015.48.5.060 PMC4578560 25845943

[191] Gruber AJ Zavolan M. Alternative Cleavage and Polyadenylation in Health and Disease. Nat Rev Genet (2019) 20:599–614. doi: 10.1038/s41576-019-0145-z 31267064

[192] Hoque M Ji Z Zheng D Luo W Li W You B. Analysis of Alternative Cleavage and Polyadenylation by 3’ Region Extraction and Deep Sequencing. Nat Methods (2013) 10:133–9. doi: 10.1038/nmeth.2288 PMC3560312 23241633

[193] Zhu H. Hu Proteins Regulate Polyadenylation by Blocking Sites Containing U-Rich Sequences*. J Biol Chem (2007) 282:8. doi: 10.1074/jbc.M609349200 17127772

[194] Miura P Shenker S Andreu-Agullo C Westholm JO Lai EC. Widespread and Extensive Lengthening of 39 Utrs in the Mammalian Brain. Genome Res (2013) 23:812–25. doi: 10.1101/gr.146886.112 PMC3638137 23520388

[195] Stevenson B Iseli C Beutler B Jongeneel C. Use of Transcriptome Data to Unravel the Fine Structure of Genes Involved in Sepsis. J Infect Dis (2003) 187:S308–14. doi: 10.1086/374755 12792844

[196] Jia X Yuan S Wang Y Fu Y Ge Y Ge Y. The Role of Alternative Polyadenylation in the Antiviral Innate Immune Response. Nat Commun (2017) 8:14605. doi: 10.1038/ncomms14605 28233779 PMC5333124

[197] Shell SA Hesse C Morris SM Milcarek C. Elevated Levels of the 64-Kda Cleavage Stimulatory Factor (Cstf-64) in Lipopolysaccharide-Stimulated Macrophages Influence Gene Expression and Induce Alternative Poly(a) Site Selection. J Biol Chem (2005) 280:39950–61. doi: 10.1074/jbc.M508848200 16207706

[198] Chen KW Demarco B Ramos S Heilig R Goris M Grayczyk JP. Ripk1 Activates Distinct Gasdermins in Macrophages and Neutrophils Upon Pathogen Blockade of Innate Immune Signaling. Proc Natl Acad Sci (2021) 118:e2101189118. doi: 10.1073/pnas.2101189118 34260403 PMC8285957

[199] Szappanos D Tschismarov R Perlot T Westermayer S Fischer K Platanitis E. The RNA Helicase Ddx3x Is an Essential Mediator of Innate Antimicrobial Immunity. PloS Pathog (2018) 14:e1007397. doi: 10.1371/journal.ppat.1007397 30475900 PMC6283616

[200] Peng Q O’Loughlin JL Humphrey MB. Dok3 Negatively Regulates Lps Responses and Endotoxin Tolerance. PloS One (2012) 7:e39967. doi: 10.1371/journal.pone.0039967 22761938 PMC3384629

[201] Marsters S Sheridan J Pitti R Huang A Skubatch M Baldwin D. A Novel Receptor for Apo2l/Trail Contains a Truncated Death Domain. Curr Biol (1997) 7:1003–6. doi: 10.1016/S0960-9822(06)00422-2 9382840

[202] Gitlin AD Heger K Schubert AF Reja R Yan D Pham VC. Integration of Innate Immune Signalling by Caspase-8 Cleavage of N4bp1. Nature (2020) 587:275–80. doi: 10.1038/s41586-020-2796-5 32971525

[203] Ingolia NT Ghaemmaghami S Newman JRS Weissman JS. Genome-Wide Analysis in Vivo of Translation With Nucleotide Resolution Using Ribosome Profiling. Science (2009) 324:218–23. doi: 10.1126/science.1168978 PMC2746483 19213877

[204] Beck A. Structure, Tissue Distribution and Genomic Organization of the Murine RRM-Type RNA Binding Proteins TIA-1 and TIAR. Nucleic Acids Res (1996) 24:3829–35. doi: 10.1093/nar/24.19.3829 PMC146163 8871565

[205] Tian Q Streuli M Saito H Schlossman SF Anderson P. A Polyadenylate Binding Protein Localized to the Granules of Cytolytic Lymphocytes Induces DNA Fragmentation in Target Cells. Cell (1991) 11:629–39. doi: 10.1016/0092-8674(91)90536-8 1934064

[206] Kawakami A Tian Q Duan X Streuli M Schlossman SF Anderson P. Identification and Functional Characterization of a TIA-1-Related Nucleolysin. Proc Natl Acad Sci (1992) 89:8681–5. doi: 10.1073/pnas.89.18.8681 PMC49984 1326761

[207] Kedersha NL Gupta M Li W Miller I Anderson P. RNA-Binding Proteins TIA-1 and TIAR Link the Phosphorylation of eIF-2 to the Assembly of Mammalian Stress Granules. J Cell Biol (1999) 147:11. doi: 10.1083/jcb.147.7.1431 PMC2174242 10613902

[208] Anderson P Kedersha N. Stress Granules: The Tao of RNA Triage. Trends Biochem Sci (2008) 33:141–50. doi: 10.1016/j.tibs.2007.12.003 18291657

[209] Gueydan C Droogmans L Chalon P Huez G Caput D Kruys V. Identification of TIAR as a Protein Binding to the Translational Regulatory AU-Rich Element of Tumor Necrosis Factor α mRNA. J Biol Chem (1999) 274:2322–6. doi: 10.1074/jbc.274.4.2322 9890998

[210] Piecyk M Wax S Beck AR Kedersha N Gupta M Maritim B. TIA-1 Is a Translational Silencer That Selectively Regulates the Expression of TNF-α. EMBO J (2000) 19:4154–63. doi: 10.1093/emboj/19.15.4154 PMC306595 10921895

[211] Naz S Battu S Khan RA Afroz S Giddaluru J Vishwakarma SK. Activation of Integrated Stress Response Pathway Regulates Il-1β Production Through Posttranscriptional and Translational Reprogramming in Macrophages. Eur J Immunol (2019) 49:277–89. doi: 10.1002/eji.201847513 30578631

[212] Loflin P Chen CYA Shyu AB. Unraveling a Cytoplasmic Role for hnRNP D in the *In Vivo* mRNA Destabilization Directed by the AU-Rich Element. Genes Dev (1999) 13:1884–97. doi: 10.1101/gad.13.14.1884 PMC316883 10421639

[213] Mukhopadhyay R Jia J Arif A Ray PS Fox PL. The GAIT System: A Gatekeeper of Inflammatory Gene Expression. Trends Biochem Sci (2009) 8:324–31. doi: 10.1016/j.tibs.2009.03.004 PMC3637685 19535251

[214] Ban N Beckmann R Cate JHD Dinman JD Dragon F Ellis SR. A New System for Naming Ribosomal Proteins. Curr Opin Struct Biol (2014) 24:165–9. doi: 10.1016/j.sbi.2014.01.002 PMC4358319 24524803

[215] Mazumder B Sampath P Seshadri V Maitra RK DiCorleto PE Fox PL. Regulated Release of L13a From the 60S Ribosomal Subunit as A Mechanism of Transcript-Specific Translational Control. Cell (2003) 12:187–98. doi: 10.1016/S0092-8674(03)00773-6 PMC13188775 14567916

[216] Yao P Potdar AA Ray PS Eswarappa SM Flagg AC Willard B. The HILDA Complex Coordinates a Conditional Switch in the 3’-Untranslated Region of the VEGFA mRNA. PloS Biol (2013) 11:e1001635. doi: 10.1371/journal.pbio.1001635 23976881 PMC3747992

[217] Arif A Yao P Terenzi F Jia J Ray PS Fox PL. The GAIT Translational Control System: GAIT System. WIREs RNA (2018) 9:e1441. doi: 10.1002/wrna.1441 PMC5815886 29152905

[218] Arif A Jia J Willard B Li X Fox PL. Multisite Phosphorylation of S6K1 Directs a Kinase Phospho-Code That Determines Substrate Selection. Mol Cell (2019) 73:446–57.e6. doi: 10.1016/j.molcel.2018.11.017 PMC6415305 30612880

[219] Poddar D Basu A Baldwin WM Kondratov RV Barik S Mazumder B. An Extraribosomal Function of Ribosomal Protein L13a in Macrophages Resolves Inflammation. J Immunol (2013) 14:3600–12. doi: 10.4049/jimmunol.1201933 PMC3608820 23460747

[220] Kapasi P Chaudhuri S Vyas K Baus D Komar AA Fox PL. L13a Blocks 48s Assembly: Role of a General Initiation Factor in mRNA-Specific Translational Control. Mol Cell (2007) 14:113–26. doi: 10.1016/j.molcel.2006.11.028 PMC1810376 17218275

[221] Chaudhuri S Vyas K Kapasi P Komar AA Dinman JD Barik S. Human Ribosomal Protein L13a Is Dispensable for Canonical Ribosome Function But Indispensable for Efficient rRNA Methylation. RNA (2007) 15:2224–37. doi: 10.1261/rna.694007 PMC2080596 17921318

[222] Poddar D Kaur R Baldwin WM Mazumder B. L13a-Dependent Translational Control in Macrophages Limits the Pathogenesis of Colitis. Cell Mol Immunol (2016) 13:816–27. doi: 10.1038/cmi.2015.53 PMC5101439 26166763

[223] Basu A Poddar D Robinet P Smith JD Febbraio M Iii WMB. Ribosomal Protein L13a Deficiency in Macrophages Promotes Atherosclerosis by Limiting Translation Control-Dependent Retardation of Inflammation. ATVB (2014) 10:533–42. doi: 10.1161/ATVBAHA.113.302573 PMC3954853 24436370

[224] Ray PS Fox PL. A Post-Transcriptional Pathway Represses Monocyte VEGF-A Expression and Angiogenic Activity. EMBO J (2007) 26:3360–72. doi: 10.1038/sj.emboj.7601774 PMC1933405 17611605

[225] Ray PS Jia J Yao P Majumder M Hatzoglou M Fox PL. A Stress-Responsive RNA Switch Regulates VEGFA Expression. Nature (2009) 457:915–9. doi: 10.1038/nature07598 PMC2858559 19098893

[226] Schoggins JW Wilson SJ Panis M Murphy MY Jones CT Bieniasz P. A Diverse Range of Gene Products Are Effectors of the Type I Interferon Antiviral Response. Nature (2011) 472:481–5. doi: 10.1038/nature09907 PMC3409588 21478870

[227] Li MM MacDonald MR Rice CM. To Translate, or Not to Translate: Viral and Host mRNA Regulation by Interferon-Stimulated Genes. Trends Cell Biol (2015) 25:320–9. doi: 10.1016/j.tcb.2015.02.001 PMC4441850 25748385

[228] Manche L Green SR Schmedt C Mathews MB. Interactions Between Double-Stranded RNA Regulators and the Protein Kinase DAI. Mol Cell Biol (1992) 12:5238–48. doi: 10.1128/MCB.12.11.5238 PMC360457 1357546

[229] McCormick C Khaperskyy DA. Translation Inhibition and Stress Granules in the Antiviral Immune Response. Nat Rev Immunol (2017) 17:647–60. doi: 10.1038/nri.2017.63 28669985

[230] Eiermann N Haneke K Sun Z Stoecklin G Ruggieri A. Dance With the Devil: Stress Granules and Signaling in Antiviral Responses. Viruses (2020) 12:E984. doi: 10.3390/v12090984 32899736 PMC7552005

[231] Reineke LC Kedersha N Langereis MA van Kuppeveld FJM Lloyd RE. Stress Granules Regulate Double-Stranded RNA-Dependent Protein Kinase Activation Through a Complex Containing G3bp1 and Caprin1. mBio (2015) 6:e02486. doi: 10.1128/mBio.02486-14 25784705 PMC4453520

[232] Law LMJ Razooky BS Li MMH You S Jurado A Rice CM. Zap’s Stress Granule Localization Is Correlated With Its Antiviral Activity and Induced by Virus Replication. PloS Pathog (2019) 15:e1007798. doi: 10.1371/journal.ppat.1007798 31116799 PMC6548403

[233] Williams GD Gokhale NS Snider DL Horner SM. The mRNA Cap 2’- *O* -Methyltransferase CMTR1 Regulates the Expression of Certain Interferon-Stimulated Genes. mSphere (2020) 5:e00202–20. doi: 10.1128/mSphere.00202-20 PMC7227766 32404510

[234] Diamond MS. Ifit1: A Dual Sensor and Effector Molecule That Detects Non-2’-O Methylated Viral RNA and Inhibits Its Translation. Cytokine Growth Factor Rev (2014) 25:543–50. doi: 10.1016/j.cytogfr.2014.05.002 PMC4234691 24909568

[235] Welsby I Hutin D Gueydan C Kruys V Rongvaux A Leo O. PARP12, an Interferon-Stimulated Gene Involved in the Control of Protein Translation and Inflammation. J Biol Chem (2014) 289:26642–57. doi: 10.1074/jbc.M114.589515 PMC4176246 25086041

[236] Leung A Vyas S Rood J Bhutkar A Sharp P Chang P. Poly(ADP-Ribose) Regulates Stress Responses and MicroRNA Activity in the Cytoplasm. Mol Cell (2011) 42:489–99. doi: 10.1016/j.molcel.2011.04.015 PMC3898460 21596313

[237] Espert L Degols G Gongora C Blondel D Williams BR Silverman RH. ISG20, a New Interferon-Induced RNase Specific for Single-Stranded RNA, Defines an Alternative Antiviral Pathway Against RNA Genomic Viruses. J Biol Chem (2003) 278:16151–8. doi: 10.1074/jbc.M209628200 12594219

[238] Liu Y Nie H Mao R Mitra B Cai D Yan R. Interferon-Inducible Ribonuclease ISG20 Inhibits Hepatitis B Virus Replication Through Directly Binding to the Epsilon Stem-Loop Structure of Viral RNA. PloS Pathog (2017) 13:e1006296. doi: 10.1371/journal.ppat.1006296 28399146 PMC5388505

[239] Wu N Nguyen XN Wang L Appourchaux R Zhang C Panthu B. The Interferon Stimulated Gene 20 Protein (ISG20) Is an Innate Defense Antiviral Factor That Discriminates Self Versus Non-Self Translation. PloS Pathog (2019) 15:e1008093. doi: 10.1371/journal.ppat.1008093 31600344 PMC6805002

[240] Zhu Y Wang X Goff SP Gao G. Translational Repression Precedes and Is Required for ZAP-Mediated mRNA Decay: ZAP-Mediated Translational Repression Versus mRNA Decay. EMBO J (2012) 31:4236–46. doi: 10.1038/emboj.2012.271 PMC3492732 23023399

[241] Zhu Y Chen G Lv F Wang X Ji X Xu Y. Zinc-Finger Antiviral Protein Inhibits HIV-1 Infection by Selectively Targeting Multiply Spliced Viral mRNAs for Degradation. Proc Natl Acad Sci (2011) 108:15834–9. doi: 10.1073/pnas.1101676108 PMC3179061 21876179

[242] Kmiec D Lista MJ Ficarelli M Swanson CM. Neil SJD. S-Farnesylation Is Essential for Antiviral Activity of the Long Zap Isoform Against RNA Viruses With Diverse Replication Strategies. PloS Pathog (2021) 17:e1009726. doi: 10.1371/journal.ppat.1009726 34695163 PMC8568172

[243] Kmiec D Nchioua R Sherrill-Mix S Stürzel CM Heusinger E Braun E. Cpg Frequency in the 5′ Third of the Env Gene Determines Sensitivity of Primary Hiv-1 Strains to the Zinc-Finger Antiviral Protein. mBio (2020) 11:e02903–19. doi: 10.1128/mBio.02903-19 PMC6960287 31937644

[244] Yoneyama M Onomoto K Jogi M Akaboshi T Fujita T. Viral RNA Detection by RIG-I-Like Receptors. Curr Opin Immunol (2015) 32:48–53. doi: 10.1016/j.coi.2014.12.012 25594890

[245] Pichlmair A Lassnig C Eberle CA Górna MW Baumann CL Burkard TR. IFIT1 Is an Antiviral Protein That Recognizes 5’-Triphosphate RNA. Nat Immunol (2011) 12:624–30. doi: 10.1038/ni.2048 21642987

[246] Abbas YM Pichlmair A Górna MW Superti-Furga G Nagar B. Structural Basis for Viral 5’-PPP-RNA Recognition by Human IFIT Proteins. Nature (2013) 494:60–4. doi: 10.1038/nature11783 PMC4931921 23334420

[247] Wang C Pflugheber J Sumpter R Sodora DL Hui D Sen GC. Alpha Interferon Induces Distinct Translational Control Programs To Suppress Hepatitis C Virus RNA Replication. JVI (2003) 77:3898–912. doi: 10.1128/JVI.77.7.3898-3912.2003 PMC150642 12634350

[248] Kimura T Katoh H Kayama H Saiga H Okuyama M Okamoto T. Ifit1 Inhibits Japanese Encephalitis Virus Replication Through Binding to 5’ Capped 2’-O Unmethylated RNA. J Virol (2013) 87:9997–10003. doi: 10.1128/JVI.00883-13 23824812 PMC3754022

[249] Roy B Jacobson A. The Intimate Relationships of mRNA Decay and Translation. Trends Genet (2013) 29:691–9. doi: 10.1016/j.tig.2013.09.002 PMC3854950 24091060

[250] Radhakrishnan A Green R. Connections Underlying Translation and mRNA Stability. J Mol Biol (2016) 428:3558–64. doi: 10.1016/j.jmb.2016.05.025 27261255

[251] Schwede A Ellis L Luther J Carrington M Stoecklin G Clayton C. A Role for Caf1 in mRNA Deadenylation and Decay in Trypanosomes and Human Cells. Nucleic Acids Res (2008) 36:3374–88. doi: 10.1093/nar/gkn108 PMC2425496 18442996

[252] Chen J Chiang YC Denis CL. CCR4, A 3’-5’ Poly(A) RNA and ssDNA Exonuclease, Is the Catalytic Component of the Cytoplasmic Deadenylase. EMBO J (2002) 21:1414–26. doi: 10.1093/emboj/21.6.1414 PMC125924 11889047

[253] She M Decker CJ Svergun DI Round A Chen N Muhlrad D. Structural Basis of Dcp2 Recognition and Activation by Dcp1. Mol Cell (2008) 29:337–49. doi: 10.1016/j.molcel.2008.01.002 PMC2323275 18280239

[254] Deshmukh MV Jones BN Quang-Dang DU Flinders J Floor SN Kim C. mRNA Decapping Is Promoted by an RNA-Binding Channel in Dcp2. Mol Cell (2008) 29:324–36. doi: 10.1016/j.molcel.2007.11.027 18280238

[255] Muhlrad D Decker CJ Parker R. Deadenylation of the Unstable mRNA Encoded by the Yeast MFA2 Gene Leads to Decapping Followed by 5'-->3' Digestion of the Transcript. Genes Dev (1994) 13:855–66. doi: 10.1101/gad.8.7.855 7926773

[256] Anderson JSJ Parker R. The 3' to 5' Degradation of Yeast mRNAs Is a General Mechanism for mRNA Turnover that Requires the SKI2 DEVH Box Protein and 3' to 5' Exonucleases of the Exosome Complex. EMBO J (1998) 10:1497–506. doi: 10.1093/emboj/17.5.1497 PMC1170497 9482746

[257] Sheth U. Decapping and Decay of Messenger RNA Occur in Cytoplasmic Processing Bodies. Science (2003) 300:805–8. doi: 10.1126/science.1082320 PMC1876714 12730603

[258] Hubstenberger A Courel M Bénard M Souquere S Ernoult-Lange M Chouaib R. P-Body Purification Reveals the Condensation of Repressed mRNA Regulons. Mol Cell (2017) 68:144–57.e5. doi: 10.1016/j.molcel.2017.09.003 28965817

[259] Courel M Clément Y Bossevain C Foretek D Vidal Cruchez O Yi Z. GC Content Shapes mRNA Storage and Decay in Human Cells. eLife (2019) 8:e49708. doi: 10.7554/eLife.49708 31855182 PMC6944446

[260] Tuck AC Rankova A Arpat AB Liechti LA Hess D Iesmantavicius V. Mammalian RNA Decay Pathways Are Highly Specialized and Widely Linked to Translation. Mol Cell (2020) 77:1222–36.e13. doi: 10.1016/j.molcel.2020.01.007 PMC7083229 32048998

[261] Wu Q Medina SG Kushawah G DeVore ML Castellano LA Hand JM. Translation Affects mRNA Stability in a Codon-Dependent Manner in Human Cells. eLife (2019) 8:e45396. doi: 10.7554/eLife.45396 31012849 PMC6529216

[262] Bicknell AA Ricci EP. When mRNA Translation Meets Decay. Biochem Soc Trans (2017) 45:339–51. doi: 10.1042/BST20160243 28408474

[263] Morris C Cluet D Ricci EP. Ribosome Dynamics and mRNA Turnover, a Complex Relationship Under Constant Cellular Scrutiny. WIREs RNA (2021) 12:e1658. doi: 10.1002/wrna.1658 33949788 PMC8519046

[264] Schoenberg DR. Mechanisms of Endonuclease-Mediated mRNA Decay: Endonuclease-Mediated mRNA Decay. WIREs RNA (2011) 2:582–600. doi: 10.1002/wrna.78 21957046 PMC3347869

[265] Bhatt D Pandya-Jones A Tong AJ Barozzi I Lissner M Natoli G. Transcript Dynamics of Proinflammatory Genes Revealed by Sequence Analysis of Subcellular RNA Fractions. Cell (2012) 150:279–90. doi: 10.1016/j.cell.2012.05.043 PMC3405548 22817891

[266] Rabani M Levin JZ Fan L Adiconis X Raychowdhury R Garber M. Metabolic Labeling of RNA Uncovers Principles of RNA Production and Degradation Dynamics in Mammalian Cells. Nat Biotechnol (2011) 29:436–42. doi: 10.1038/nbt.1861 PMC3114636 21516085

[267] Paulsen MT Veloso A Prasad J Bedi K Ljungman EA Tsan YC. Coordinated Regulation of Synthesis and Stability of RNA During the Acute Tnf-Induced Proinflammatory Response. Proc Natl Acad Sci (2013) 110:2240–5. doi: 10.1073/pnas.1219192110 PMC3568384 23345452

[268] Bergen V Lange M Peidli S Wolf FA Theis FJ. Generalizing RNA Velocity to Transient Cell States Through Dynamical Modeling. Nat Biotechnol (2020) 38:1408–14. doi: 10.1038/s41587-020-0591-3 32747759

[269] Furlan M Galeota E Gaudio ND Dassi E Caselle M de Pretis S. Genome-Wide Dynamics of RNA Synthesis, Processing, and Degradation Without RNA Metabolic Labeling. Genome Res (2020) 30:1492–507. doi: 10.1101/gr.260984.120 PMC7605262 32978246

[270] Chen CYA Shyu AB. AU-Rich Elements: Characterization and Importance in mRNA Degradation. Trends Biochem Sci (1995) 20:465–70. doi: 10.1016/S0968-0004(00)89102-1 8578590

[271] Zubiaga AM Belasco JG Greenberg ME. The Nonamer Uuauuuauu Is the Key Au-Rich Sequence Motif That Mediates Mrna Degradation. Mol Cell Biol (1995) 15:2219–30. doi: 10.1128/MCB.15.4.2219 PMC230450 7891716

[272] Lal A Mazan-Mamczarz K Kawai T Yang X Martindale JL Gorospe M. Concurrent Versus Individual Binding of Hur and Auf1 to Common Labile Target Mrnas. EMBO J (2004) 23:3092–102. doi: 10.1038/sj.emboj.7600305 PMC514922 15257295

[273] Hopkins TG Mura M Al-Ashtal HA Lahr RM Abd-Latip N Sweeney K. The RNA-Binding Protein Larp1 Is a Post-Transcriptional Regulator of Survival and Tumorigenesis in Ovarian Cancer. Nucleic Acids Res (2016) 44:1227–46. doi: 10.1093/nar/gkv1515 PMC4756840 26717985

[274] Wigington CP Jung J Rye EA Belauret SL Philpot AM Feng Y. Post-Transcriptional Regulation of Programmed Cell Death 4 (Pdcd4) Mrna by the RNA-Binding Proteins Human Antigen R (Hur) and T-Cell Intracellular Antigen 1 (Tia1). J Biol Chem (2015) 290:3468–87. doi: 10.1074/jbc.M114.631937 PMC4319015 25519906

[275] Dassi E. Handshakes and Fights: The Regulatory Interplay of RNA-Binding Proteins. Front Mol Biosci (2017) 4:67. doi: 10.3389/fmolb.2017.00067 29034245 PMC5626838

[276] Shaw G Kamen R. A Conserved Au Sequence From the 3’ Untranslated Region of Gm-Csf Mrna Mediates Selective Mrna Degradation. Cell (1986) 46:659–67. doi: 10.1016/0092-8674(86)90341-7 3488815

[277] Caput D Beutler B Hartog K Thayer R Brown-Shimer S Cerami A. Identification of a Common Nucleotide Sequence in the 3’-Untranslated Region of Mrna Molecules Specifying Inflammatory Mediators. Proc Natl Acad Sci (1986) 83:1670–4. doi: 10.1073/pnas.83.6.1670 PMC323145 2419912

[278] Hao S Baltimore D. The Stability of mRNA Influences the Temporal Order of the Induction of Genes Encoding Inflammatory Molecules. Nat Immunol (2009) 10:281–8. doi: 10.1038/ni.1699 PMC2775040 19198593

[279] Ivanov P Anderson P. Post-Transcriptional Regulatory Networks in Immunity. Immunol Rev (2013) 253:253–72. doi: 10.1111/imr.12051 PMC6989036 23550651

[280] Lai WS Stumpo DJ Blackshear PJ. Rapid Insulin-Stimulated Accumulation of an mRNA Encoding a Proline-Rich Protein. J Biol Chem (1990) 265:16556–63. doi: 10.1016/S0021-9258(17)46259-4 2204625

[281] Taylor GA Lai WS Oakey RJ Seldin MF Shows TB Eddy RL. The Human TTP Protein: Sequence, Alignment With Related Proteins, and Chromosomal Localization of the Mouse and Human Genes. Nucl Acids Res (1991) 19:3454–4. doi: 10.1093/nar/19.12.3454 PMC328350 2062660

[282] Thompson MJ Lai WS Taylor GA Blackshear PJ. Cloning and Characterization of Two Yeast Genes Encoding Members of the CCCH Class of Zinc Finger Proteins: Zinc Finger-Mediated Impairment of Cell Growth. Gene (1996) 174:225–33. doi: 10.1016/0378-1119(96)00084-4 8890739

[283] Carballo E. Feedback Inhibition of Macrophage Tumor Necrosis Factor- Production by Tristetraprolin. Science (1998) 281:1001–5. doi: 10.1126/science.281.5379.1001 9703499

[284] Lai WS Carballo E Thorn JM Kennington EA Blackshear PJ. Interactions of CCCH Zinc Finger Proteins With mRNA. J Biol Chem (2000) 275:17827–37. doi: 10.1074/jbc.M001696200 10751406

[285] Carballo E Lai WS Blackshear PJ. Evidence That Tristetraprolin Is a Physiological Regulator of Granulocyte-Macrophage Colony-Stimulating Factor Messenger RNA Deadenylation and Stability. Blood (2000) 95:1891–9. doi: 10.1182/blood.V95.6.1891 10706852

[286] Fabian MR Frank F Rouya C Siddiqui N Lai WS Karetnikov A. Structural Basis for the Recruitment of the Human CCR4–NOT Deadenylase Complex by Tristetraprolin. Nat Struct Mol Biol (2013) 20:735–9. doi: 10.1038/nsmb.2572 PMC4811204 23644599

[287] Sun L Stoecklin G Van Way S Hinkovska-Galcheva V Guo RF Anderson P. Tristetraprolin (Ttp)-14-3-3 Complex Formation Protects Ttp From Dephosphorylation by Protein Phosphatase 2a and Stabilizes Tumor Necrosis Factor-Alpha mRNA. J Biol Chem (2007) 282:3766–77. doi: 10.1074/jbc.M607347200 17170118

[288] Lykke-Andersen J Wagner E. Recruitment and Activation of Mrna Decay Enzymes by Two Are-Mediated Decay Activation Domains in the Proteins Ttp and Brf-1. Genes Dev (2005) 19:351–61. doi: 10.1101/gad.1282305 PMC546513 15687258

[289] Bulbrook D Brazier H Mahajan P Kliszczak M Fedorov O Marchese F. Tryptophan-Mediated Interactions Between Tristetraprolin and the Cnot9 Subunit Are Required for Ccr4-Not Deadenylase Complex Recruitment. J Mol Biol (2018) 430:722–36. doi: 10.1016/j.jmb.2017.12.018 29291391

[290] Mino T Murakawa Y Fukao A Vandenbon A Wessels HH Ori D. Regnase-1 and Roquin Regulate a Common Element in Inflammatory mRNAs by Spatiotemporally Distinct Mechanisms. Cell (2015) 161:1058–73. doi: 10.1016/j.cell.2015.04.029 26000482

[291] Maeda K Akira S. Regulation of Mrna Stability by Ccch-Type Zinc-Finger Proteins in Immune Cells. Int Immunol (2017) 29:149–55. doi: 10.1093/intimm/dxx015 PMC5890888 28369485

[292] Fenger-Grøn M Fillman C Norrild B Lykke-Andersen J. Multiple Processing Body Factors and the ARE Binding Protein TTP Activate mRNA Decapping. Mol Cell (2005) 20:905–15. doi: 10.1016/j.molcel.2005.10.031 16364915

[293] Stoecklin G Stubbs T Kedersha N Wax S Rigby WF Blackwell TK. MK2-Induced Tristetraprolin:14-3-3 Complexes Prevent Stress Granule Association and ARE-mRNA Decay. EMBO J (2004) 23:1313–24. doi: 10.1038/sj.emboj.7600163 PMC381421 15014438

[294] Tiedje C Diaz-Muñoz MD Trulley P Ahlfors H Laaß K Blackshear PJ. The RNA-Binding Protein TTP Is a Global Post-Transcriptional Regulator of Feedback Control in Inflammation. Nucleic Acids Res (2016) 44:gkw474. doi: 10.1093/nar/gkw474 PMC5009735 27220464

[295] Tiedje C Ronkina N Tehrani M Dhamija S Laass K Holtmann H. The P38/MK2-Driven Exchange Between Tristetraprolin and HuR Regulates AU–Rich Element–Dependent Translation. PloS Genet (2012) 8:e1002977. doi: 10.1371/journal.pgen.1002977 23028373 PMC3459988

[296] Schott J Reitter S Philipp J Haneke K Schäfer H Stoecklin G. Translational Regulation of Specific mRNAs Controls Feedback Inhibition and Survival During Macrophage Activation. PloS Genet (2014) 10:e1004368. doi: 10.1371/journal.pgen.1004368 24945926 PMC4063670

[297] Sedlyarov V Fallmann J Ebner F Huemer J Sneezum L Ivin M. Tristetraprolin Binding Site Atlas in the Macrophage Transcriptome Reveals a Switch for Inflammation Resolution. Mol Syst Biol (2016) 12:868. doi: 10.15252/msb.20156628 27178967 PMC4988506

[298] Taylor GA Carballo E Lee DM Lai WS Thompson MJ Patel DD. A Pathogenetic Role for Tnfα in the Syndrome of Cachexia, Arthritis, and Autoimmunity Resulting From Tristetraprolin (TTP) Deficiency. Tumor Necrosis Factor (1996) 4:10. doi: 10.1016/S1074-7613(00)80411-2 8630730

[299] Stoecklin G Ming XF Looser R Moroni C. Somatic mRNA Turnover Mutants Implicate Tristetraprolin in the Interleukin-3 mRNA Degradation Pathway. Mol Cell Biol (2000) 20:3753–63. doi: 10.1128/MCB.20.11.3753-3763.2000 PMC85689 10805719

[300] Stoecklin G Lu M Rattenbacher B Moroni C. A Constitutive Decay Element Promotes Tumor Necrosis Factor Alpha Mrna Degradation *via* an Au-Rich Element-Independent Pathway. Mol Cell Biol (2003) 23:3506–15. doi: 10.1128/MCB.23.10.3506-3515.2003 PMC164766 12724409

[301] Leppek K Schott J Reitter S Poetz F Hammond MC Stoecklin G. Roquin Promotes Constitutive mRNA Decay *via* a Conserved Class of Stem-Loop Recognition Motifs. Cell (2013) 153:869–81. doi: 10.1016/j.cell.2013.04.016 23663784

[302] Schaefer JS Klein JR. Roquin—A Multifunctional Regulator of Immune Homeostasis. Genes Immun (2016) 17:79–84. doi: 10.1038/gene.2015.58 26673963 PMC4777649

[303] Moraes KC. Cug-Bp Binds to RNA Substrates and Recruits Parn Deadenylase. RNA (2006) 12:1084–91. doi: 10.1261/rna.59606 PMC1464848 16601207

[304] Antic S Wolfinger MT Skucha A Hosiner S Dorner S. General and MicroRNA-Mediated mRNA Degradation Occurs on Ribosome Complexes in Drosophila Cells. Mol Cell Biol (2015) 35:2309–20. doi: 10.1128/MCB.01346-14 PMC4456442 25918245

[305] Tat TT Maroney PA Chamnongpol S Coller J Nilsen TW. Cotranslational microRNA Mediated Messenger RNA Destabilization. eLife (2016) 5:e12880. doi: 10.7554/eLife.12880 27058298 PMC4859803

[306] Biasini A Abdulkarim B Pretis S Tan JY Arora R Wischnewski H. Translation Is Required for miRNA-Dependent Decay of Endogenous Transcripts. EMBO J (2021) 40:e104569. doi: 10.15252/embj.2020104569 33300180 PMC7849302

[307] Matsushita K Takeuchi O Standley DM Kumagai Y Kawagoe T Miyake T. Zc3h12a Is an RNase Essential for Controlling Immune Responses by Regulating mRNA Decay. Nature (2009) 458:1185–90. doi: 10.1038/nature07924 19322177

[308] Uehata T Takeuchi O. Post-Transcriptional Regulation of Immunological Responses by Regnase-1-Related Rnases. Int Immunol (2021) 33:dxab048. doi: 10.1093/intimm/dxab048 34320195

[309] Mizgalska D Węgrzyn P Murzyn K Kasza A Koj A Jura J. Interleukin-1-Inducible MCPIP Protein Has Structural and Functional Properties of RNase and Participates in Degradation of IL-1β mRNA: MCPIP Protein as an RNase. FEBS J (2009) 276:7386–99. doi: 10.1111/j.1742-4658.2009.07452.x 19909337

[310] Dobosz E Wilamowski M Lech M Bugara B Jura J Potempa J. MCPIP-1, Alias Regnase-1, Controls Epithelial Inflammation by Posttranscriptional Regulation of IL-8 Production. J Innate Immun (2016) 8:564–78. doi: 10.1159/000448038 PMC5089914 27513529

[311] Mino T Iwai N Endo M Inoue K Akaki K Hia F. Translation-Dependent Unwinding of Stem–Loops by UPF1 Licenses Regnase-1 to Degrade Inflammatory mRNAs. Nucleic Acids Res (2019) 47:8838–59. doi: 10.1093/nar/gkz628 PMC7145602 31329944

[312] Bartel DP. MicroRNAs: Target Recognition and Regulatory Functions. Cell (2009) 136:215–33. doi: 10.1016/j.cell.2009.01.002 PMC3794896 19167326

[313] Friedman RC Farh KKH Burge CB Bartel DP. Most Mammalian mRNAs Are Conserved Targets of microRNAs. Genome Res (2008) 19:92–105. doi: 10.1101/gr.082701.108 18955434 PMC2612969

[314] Djuranovic S Nahvi A Green R. miRNA-Mediated Gene Silencing by Translational Repression Followed by mRNA Deadenylation and Decay. Science (2012) 336:237–40. doi: 10.1126/science.1215691 PMC3971879 22499947

[315] Alivernini S Gremese E McSharry C Tolusso B Ferraccioli G McInnes IB. MicroRNA-155—at the Critical Interface of Innate and Adaptive Immunity in Arthritis. Front Immunol (2018) 8:1932. doi: 10.3389/fimmu.2017.01932 29354135 PMC5760508

[316] O’Connell RM Taganov KD Boldin MP Cheng G Baltimore D. MicroRNA-155 Is Induced During the Macrophage Inflammatory Response. Proc Natl Acad Sci (2007) 104:1604–9. doi: 10.1073/pnas.0610731104 PMC1780072 17242365

[317] Xu H Xu SJ Xie SJ Zhang Y Yang JH Zhang WQ. MicroRNA-122 Supports Robust Innate Immunity in Hepatocytes by Targeting the RTKs/STAT3 Signaling Pathway. eLife (2019) 8:e41159. doi: 10.7554/eLife.41159 30735121 PMC6389286

[318] Quinn SR Mangan NE Caffrey BE Gantier MP Williams BR Hertzog PJ. The Role of Ets2 Transcription Factor in the Induction of Microrna-155 (Mir-155) by Lipopolysaccharide and Its Targeting by Interleukin-10. J Biol Chem (2014) 289:4316–25. doi: 10.1074/jbc.M113.522730 PMC3924294 24362029

[319] Seo G Kincaid R Phanaksri T Burke J Pare J Cox J. Reciprocal Inhibition Between Intracellular Antiviral Signaling and the RNAi Machinery in Mammalian Cells. Cell Host Microbe (2013) 14:435–45. doi: 10.1016/j.chom.2013.09.002 PMC3837626 24075860

[320] Backes S Shapiro J Sabin L Pham A Reyes I Moss B. Degradation of Host MicroRNAs by Poxvirus Poly(A) Polymerase Reveals Terminal RNA Methylation as a Protective Antiviral Mechanism. Cell Host Microbe (2012) 12:200–10. doi: 10.1016/j.chom.2012.05.019 PMC3782087 22901540

[321] Pegtel DM Cosmopoulos K Thorley-Lawson DA van Eijndhoven MAJ Hopmans ES Lindenberg JL. Functional Delivery of Viral miRNAs *via* Exosomes. Proc Natl Acad Sci (2010) 107:6328–33. doi: 10.1073/pnas.0914843107 PMC2851954 20304794

[322] Momen-Heravi F Bala S Kodys K Szabo G. Exosomes Derived From Alcohol-Treated Hepatocytes Horizontally Transfer Liver Specific miRNA-122 and Sensitize Monocytes to LPS. Sci Rep (2015) 5:9991. doi: 10.1038/srep09991 25973575 PMC4650752

[323] Saha B Momen-Heravi F Kodys K Szabo G. MicroRNA Cargo of Extracellular Vesicles From Alcohol-Exposed Monocytes Signals Naive Monocytes to Differentiate Into M2 Macrophages. J Biol Chem (2016) 291:149–59. doi: 10.1074/jbc.M115.694133 PMC4697152 26527689

[324] Montecalvo A Larregina AT Shufesky WJ Beer Stolz D Sullivan MLG Karlsson JM. Mechanism of Transfer of Functional microRNAs Between Mouse Dendritic Cells *via* Exosomes. Blood (2012) 119:756–66. doi: 10.1182/blood-2011-02-338004 PMC3265200 22031862

[325] Mittelbrunn M Gutiérrez-Vázquez C Villarroya-Beltri C González S Sánchez-Cabo F González MÃ. Unidirectional Transfer of microRNA-Loaded Exosomes From T Cells to Antigen-Presenting Cells. Nat Commun (2011) 2:282. doi: 10.1038/ncomms1285 21505438 PMC3104548

[326] Higa M Oka M Fujihara Y Masuda K Yoneda Y Kishimoto T. Regulation of Inflammatory Responses by Dynamic Subcellular Localization of RNA-Binding Protein Arid5a. Proc Natl Acad Sci (2018) 115:E1214–20. doi: 10.1073/pnas.1719921115 PMC5819453 29358370

[327] Minoda Y Saeki K Aki D Takaki H Sanada T Koga K. A Novel Zinc finger Protein, ZCCHC11, Interacts With TIFA and Modulates TLR Signaling. Biochem Biophys Res Commun (2006) 10:1023–30. doi: 10.1016/j.bbrc.2006.04.006 16643855

[328] Jones MR Quinton LJ Blahna MT Neilson JR Fu S Ivanov AR. Zcchc11-Dependent Uridylation of microRNA Directs Cytokine Expression. Nat Cell Biol (2009) 11:1157–63. doi: 10.1038/ncb1931 PMC2759306 19701194

[329] Li F Hu DY Liu S Mahavadi S Yen W Murthy KS. RNA-Binding Protein HuR Regulates RGS4 mRNA Stability in Rabbit Colonic Smooth Muscle Cells. Am J Physiology-Cell Physiol (2010) 299:C1418–29. doi: 10.1152/ajpcell.00093.2010 PMC3006324 20881234

[330] Fan XC Steitz JA. HNS, a Nuclear-Cytoplasmic Shuttling Sequence in HuR. Proc Natl Acad Sci (1998) 95:15293–8. doi: 10.1073/pnas.95.26.15293 PMC28036 9860962

[331] Scheiba RM de Opakua AI Díaz-Quintana A Martínez-Cruz LA Martínez-Chantar ML Díaz-Moreno I. The C-Terminal RNA Binding Motif of HuR Is a Multi-Functional Domain Leading to HuR Oligomerization. RNA Biol (2014) 11:13. doi: 10.1080/15476286.2014.996069 PMC4615805 25584704

[332] Herdy B Karonitsch T Vladimer GI Tan CS Stukalov A Trefzer C. The RNA-Binding Protein Hur/Elavl1 Regulates Ifn-β Mrna Abundance and the Type I Ifn Response: Innate Immunity. Eur J Immunol (2015) 45:1500–11. doi: 10.1002/eji.201444979 25678110

[333] Rothamel K Arcos S Kim B Reasoner C Lisy S Mukherjee N. Elavl1 Primarily Couples Mrna Stability With the 3′ Utrs of Interferon-Stimulated Genes. Cell Rep (2021) 35:109178. doi: 10.1016/j.celrep.2021.109178 34038724 PMC8225249

[334] Sueyoshi T Kawasaki T Kitai Y Ori D Akira S Kawai T. Hu Antigen R Regulates Antiviral Innate Immune Responses Through the Stabilization of Mrna for Polo-Like Kinase 2. J Immunol (2018) 200:3814–24. doi: 10.4049/jimmunol.1701282 29678949

[335] Mancino A Natoli G. Specificity and Function of Irf Family Transcription Factors: Insights From Genomics. J Interferon Cytokine Res (2016) 36:462–9. doi: 10.1089/jir.2016.0004 27379868

[336] Mukherjee N. Integrative Regulatory Mapping Indicates That the RNA-Binding Protein HuR Couples Pre-mRNA Processing and mRNA Stability. Mol Cell (2011) 13:327–39. doi: 10.1016/j.molcel.2011.06.007 PMC3220597 21723170

[337] Ku Y Park JH Cho R Lee Y Park HM Kim M. Noncanonical Immune Response to the Inhibition of DNA Methylation by Staufen1 *via* Stabilization of Endogenous Retrovirus RNAs. Proc Natl Acad Sci USA (2021) 118:e2016289118. doi: 10.1073/pnas.2016289118 33762305 PMC8020767

[338] Ye C Yu Z Xiong Y Wang Y Ruan Y Guo Y. STAU1 Binds to IBDV Genomic Double-Stranded RNA and Promotes Viral Replication *via* Attenuation of MDA5-Dependent β Interferon Induction. FASEB J (2019) 33:286–300. doi: 10.1096/fj.201800062RR 29979632

[339] Minervini CF Parciante E Impera L Anelli L Zagaria A Specchia G. Epitranscriptomics in Normal and Malignant Hematopoiesis. IJMS (2020) 21:6578. doi: 10.3390/ijms21186578 PMC7555315 32916783

[340] Tajaddod M Jantsch MF Licht K. The Dynamic Epitranscriptome: A to I Editing Modulates Genetic Information. Chromosoma (2016) 125:51–63. doi: 10.1007/s00412-015-0526-9 26148686 PMC4761006

[341] Helm M Motorin Y. Detecting RNA Modifications in the Epitranscriptome: Predict and Validate. Nat Rev Genet (2017) 18:275–91. doi: 10.1038/nrg.2016.169 28216634

[342] Kumar S Mohapatra T. Deciphering Epitranscriptome: Modification of Mrna Bases Provides a New Perspective for Post-Transcriptional Regulation of Gene Expression. Front Cell Dev Biol (2021) 9:628415. doi: 10.3389/fcell.2021.628415 33816473 PMC8010680

[343] Nachtergaele S He C. Chemical Modifications in the Life of an mRNA Transcript. Annu Rev Genet (2018) 52:349–72. doi: 10.1146/annurev-genet-120417-031522 PMC6436393 30230927

[344] Liu N Pan T. N6-Methyladenosine–Encoded Epitranscriptomics. Nat Struct Mol Biol (2016) 23:98–102. doi: 10.1038/nsmb.3162 26840897

[345] Solomon O Di Segni A Cesarkas K Porath HT Marcu-Malina V Mizrahi O. RNA Editing by Adar1 Leads to Context-Dependent Transcriptome-Wide Changes in RNA Secondary Structure. Nat Commun (2017) 8:1440. doi: 10.1038/s41467-017-01458-8 29129909 PMC5682290

[346] Nishikura K. A-To-I Editing of Coding and Non-Coding Rnas by Adars. Nat Rev Mol Cell Biol (2016) 17:83–96. doi: 10.1038/nrm.2015.4 26648264 PMC4824625

[347] Gray MW Charette M. Pseudouridine in RNA: What, Where, How, and Why. IUBMB Life (International Union Biochem Mol Biol: Life) (2000) 49:341–51. doi: 10.1080/152165400410182 10902565

[348] Mannion N Greenwood SM Young R Cox S Brindle J Read D. The RNA-Editing Enzyme ADAR1 Controls Innate Immune Responses to RNA. Cell Rep (2014) 9:1482–94. doi: 10.1016/j.celrep.2014.10.041 PMC4542304 25456137

[349] Yoshinaga M Takeuchi O. Post-Transcriptional Control of Immune Responses and Its Potential Application. Clin Trans Immunol (2019) 8:e1063. doi: 10.1002/cti2.1063 PMC6580065 31236273

[350] Jing FY Zhou LM Ning YJ Wang XJ Zhu YM. The Biological Function, Mechanism, and Clinical Significance of M6a RNA Modifications in Head and Neck Carcinoma: A Systematic Review. Front Cell Dev Biol (2021) 9:683254. doi: 10.3389/fcell.2021.683254 34136491 PMC8201395

[351] Baquero-Perez B Geers D Díez J. From a to M6a: The Emerging Viral Epitranscriptome. Viruses (2021) 13:1049. doi: 10.3390/v13061049 34205979 PMC8227502

[352] Liddicoat BJ Piskol R Chalk AM Ramaswami G Higuchi M Hartner JC. RNA Editing by Adar1 Prevents Mda5 Sensing of Endogenous Dsrna as Nonself. Science (2015) 349:1115–20. doi: 10.1126/science.aac7049 PMC5444807 26275108

[353] Lamers MM van den Hoogen BG Haagmans BL. ADAR1: “Editor-In-Chief” of Cytoplasmic Innate Immunity. Front Immunol (2019) 10:1763. doi: 10.3389/fimmu.2019.01763 31404141 PMC6669771

[354] Schlee M Hartmann G. Discriminating Self From Non-Self in Nucleic Acid Sensing. Nat Rev Immunol (2016) 16:566–80. doi: 10.1038/nri.2016.78 PMC7097691 27455396

[355] Zhang Y Wang X Zhang X Wang J Ma Y Zhang L. RNA-Binding Protein YTHDF3 Suppresses Interferon-Dependent Antiviral Responses by Promoting FOXO3 Translation. Proc Natl Acad Sci USA (2019) 116:976–81. doi: 10.1073/pnas.1812536116 PMC6338863 30591559

[356] Zhang X Flavell RA Li HB. Hnrnpa2b1: A Nuclear Dna Sensor in Antiviral Immunity. Cell Res (2019) 29:879–80. doi: 10.1038/s41422-019-0226-8 PMC6889404 31471560

[357] Wang L Wen M Cao X. Nuclear Hnrnpa2b1 Initiates and Amplifies the Innate Immune Response to Dna Viruses. Sci (New York NY) (2019) 365:eaav0758. doi: 10.1126/science.aav0758 31320558

[358] Yang Y Hsu PJ Chen YS Yang YG. Dynamic Transcriptomic M6a Decoration: Writers, Erasers, Readers and Functions in RNA Metabolism. Cell Res (2018) 28:616–24. doi: 10.1038/s41422-018-0040-8 PMC5993786 29789545

[359] Lee H Bao S Qian Y Geula S Leslie J Zhang C. Stage-Specific Requirement for Mettl3 -Dependent M 6 A mRNA Methylation During Haematopoietic Stem Cell Differentiation. Nat Cell Biol (2019) 1:700–9. doi: 10.1038/s41556-019-0318-1 PMC6556891 31061465

[360] Eisenberg E Levanon EY. A-To-I RNA Editing — Immune Protector and Transcriptome Diversifier. Nat Rev Genet (2018) 19:473–90. doi: 10.1038/s41576-018-0006-1 29692414

[361] Sadeq S Al-Hashimi S Cusack CM Werner A. Endogenous Double-Stranded RNA. Non-Coding RNA (2021) 7:15. doi: 10.3390/ncrna7010015 33669629 PMC7930956

[362] Barak M Porath HT Finkelstein G Knisbacher BA Buchumenski I Roth SH. Purifying Selection of Long dsRNA Is the First Line of Defense Against False Activation of Innate Immunity. Genome Biol (2020) 21:26. doi: 10.1186/s13059-020-1937-3 32028986 PMC7006430

[363] Rice GI Kasher PR Forte GMA Mannion NM Greenwood SM Szynkiewicz M. Mutations in Adar1 Cause Aicardi-Goutières Syndrome Associated With a Type I Interferon Signature. Nat Genet (2012) 44:1243–8. doi: 10.1038/ng.2414 PMC4154508 23001123

[364] Pujantell M Franco S Galván-Femenía I Badia R Castellví M Garcia-Vidal E. ADAR1 Affects HCV Infection by Modulating Innate Immune Response. Antiviral Res (2018) 156:116–27. doi: 10.1016/j.antiviral.2018.05.012 29906476

[365] Nie Y Hammond GL Yang JH. Double-Stranded RNA Deaminase Adar1 Increases Host Susceptibility to Virus Infection. J Virol (2007) 81:917–23. doi: 10.1128/JVI.01527-06 PMC1797455 17079286

[366] Zhou S Yang C Zhao F Huang Y Lin Y Huang C. Double-Stranded RNA Deaminase Adar1 Promotes the Zika Virus Replication by Inhibiting the Activation of Protein Kinase Pkr. J Biol Chem (2019) 294:18168–80. doi: 10.1074/jbc.RA119.009113 PMC6885634 31636123

[367] Clerzius G Gélinas JF Daher A Bonnet M Meurs EF Gatignol A. Adar1 Interacts With Pkr During Human Immunodeficiency Virus Infection of Lymphocytes and Contributes to Viral Replication. J Virol (2009) 83:10119–28. doi: 10.1128/JVI.02457-08 PMC2747994 19605474

[368] Toth AM Li Z Cattaneo R Samuel CE. RNA-Specific Adenosine Deaminase Adar1 Suppresses Measles Virus-Induced Apoptosis and Activation of Protein Kinase Pkr. J Biol Chem (2009) 284:29350–6. doi: 10.1074/jbc.M109.045146 PMC2785566 19710021

[369] Nallagatla SR Toroney R Bevilacqua PC. A Brilliant Disguise for Self RNA: 5’-End and Internal Modifications of Primary Transcripts Suppress Elements of Innate Immunity. RNA Biol (2008) 5:140–4. doi: 10.4161/rna.5.3.6839 PMC2809118 18769134

[370] Vogel OA Han J Liang CY Manicassamy S Perez JT Manicassamy B. The P150 Isoform of Adar1 Blocks Sustained Rlr Signaling and Apoptosis During Influenza Virus Infection. PloS Pathog (2020) 16:e1008842. doi: 10.1371/journal.ppat.1008842 32898178 PMC7500621

[371] Karikó K Buckstein M Ni H Weissman D. Suppression of RNA Recognition by Toll-Like Receptors: The Impact of Nucleoside Modification and the Evolutionary Origin of RNA. Immunity (2005) 23:165–75. doi: 10.1016/j.immuni.2005.06.008 16111635

[372] Chen YG Chen R Ahmad S Verma R Kasturi SP Amaya L. N6-Methyladenosine Modification Controls Circular RNA Immunity. Mol Cell (2019) 76:96–109.e9. doi: 10.1016/j.molcel.2019.07.016 31474572 PMC6778039

[373] Bokar JA Shambaugh ME Polayes D Matera AG Rottman FM. Purification and cDNA Cloning of the AdoMet-Binding Subunit of the Human mRNA (N6-Adenosine)-Methyltransferase. RNA (1997) 3:1233–47. PMC1369564 9409616

[374] Wang H Hu X Huang M Liu J Gu Y Ma L. Mettl3-Mediated mRNA M6a Methylation Promotes Dendritic Cell Activation. Nat Commun (2019) 10:1898. doi: 10.1038/s41467-019-09903-6 31015515 PMC6478715

[375] Wang X. N6-Methyladenosine Modulates Messenger RNA Translation Efficiency. Cell (2015) 13:1388–99. doi: 10.1016/j.cell.2015.05.014 PMC4825696 26046440

[376] Zheng Q Hou J Zhou Y Li Z Cao X. The RNA Helicase Ddx46 Inhibits Innate Immunity by Entrapping M6a-Demethylated Antiviral Transcripts in the Nucleus. Nat Immunol (2017) 18:1094–103. doi: 10.1038/ni.3830 28846086

[377] Yoshinaga M Takeuchi O. RNA Binding Proteins in the Control of Autoimmune Diseases. Immunol Med (2019) 42:53–64. doi: 10.1080/25785826.2019.1655192 31449478

[378] Quattrone A Dassi E. The Architecture of the Human RNA-Binding Protein Regulatory Network. iScience (2019) 21:706–19. doi: 10.1016/j.isci.2019.10.058 PMC6864347 31733516

